# In silico characterization and homology modeling of cytosolic *APX* gene predicts novel glycine residue modulating waterlogging stress response in pigeon pea

**DOI:** 10.7717/peerj.10888

**Published:** 2021-05-12

**Authors:** Anshika Tyagi, Sandhya Sharma, Harsha Srivastava, Nagendra Kumar Singh, Kishor Gaikwad

**Affiliations:** ICAR- National Institute for Plant Biotechnology, New Delhi, India

**Keywords:** Ascorbate peroxidase, Molecular modeling, Docking, Simulation, Pigeon pea, Waterlogging tolerance

## Abstract

Ascorbate peroxidase (*APX*) is a member of the family of heme-containing peroxidases having a similar structure with Cytochrome c peroxidase (CCP) that effectively scavenge cytosolic and chloroplastic hydrogen peroxide (H_2_O_2_) under various stresses. In this study, computational characterization and homology analysis of *APX* protein from waterlogging tolerant (ICPL 84023) and sensitive (ICP 7035) pigeon pea genotypes were carried out resulting in 100% homology with *Glycine max* in case of former and 99% in later genotypes respectively with 97.39% alignment coverage among each other. The model structure was further refined by various tools like PROCHECK, ProSA, and Verify3D. The planned model of the *APX* enzyme was then tested to dock with H_2_O_2_along with molecular dynamics (MD) simulation analysis. The docked complex of ICPL 84023 showed the best G-score (23.39 kcal/mol) in comparison to ICP 7035 (16.74 kcal/mol) depicting the higher production of *APX* for scavenging reactive oxygen species (ROS) production making this genotype more tolerant. The important binding residues in the ICPL 84023-H_2_O_2_complex (SER1, THR4, GLU23, and GLY13) have shown less fluctuation than the ICP 7035-H_2_O_2_ complex (SER1, THR4, and GLU23). Overall, our results showed that amino acid residue glycine in ICPL 84023 *APX* gene has a high binding affinity with H_2_O_2_ which could be a key factor associated with waterlogging stress tolerance in pigeon pea.

## Introduction

Pigeon pea (*Cajanus cajan*) is a drought-tolerant legume crop, belonging to the family ”Fabaceae”. Among various abiotic stresses, waterlogging is the main constraint to pigeon pea productivity worldwide (Sultana et al., 2013). The main cause of plant damage due to waterlogging is the low supply of oxygen to the tissues which hampers nutrient and water uptake, slows diffusion of gasses and ultimately leads to wilting of plants. Under such conditions, the plant switches its metabolism from aerobic to anaerobic respiration. To cope up with this oxidative stress induced by waterlogging, plants use diverse defense mechanisms such as higher accumulation of compatible solutes or sugars, the formation of aerenchyma, and accelerate its metabolic pathways including glycolysis and other components involving anaerobic energy production (Steffens et al., 2012; Steffens, Steffen-Heins & Sauter, 2013). Besides, plants also fight against waterlogging stress by increasing the activity of antioxidant enzymes. Survival of plants under any abiotic/biotic condition is determined by the physiological balance between antioxidant defense enzyme and oxidative stress induced by an elevated level of reactive oxygen species (ROS) and reactive nitrogen species (RNS). ROS production during waterlogging stress is an essential component of hypoxia signaling and also promotes the healthy growth and development of plants. It is formed either enzymatically or non-enzymatically by stepwise molecular oxygen loss including superoxide (O_2_^.−^), hydrogen peroxide (H_2_O_2_), singlet oxygen, and the hydroxyl radical. The effective scavenging system includes a series of antioxidant defense enzymes such as superoxide dismutase (*SOD*), catalase (*CAT*), ascorbate peroxidase (*APX*), and some low molecular weight antioxidant molecules like glutathione peroxidase *(GPX),* glutathione, ascorbate, and α-tocopherol. These scavenging systems in plants play an important role in combating excess ROS and nitric oxide synthase (NOS) production (Mittler et al., 2004; Yamauchi et al., 2013; Yamauchi et al., 2017; Gonzali et al., 2015).

**Figure 1:**
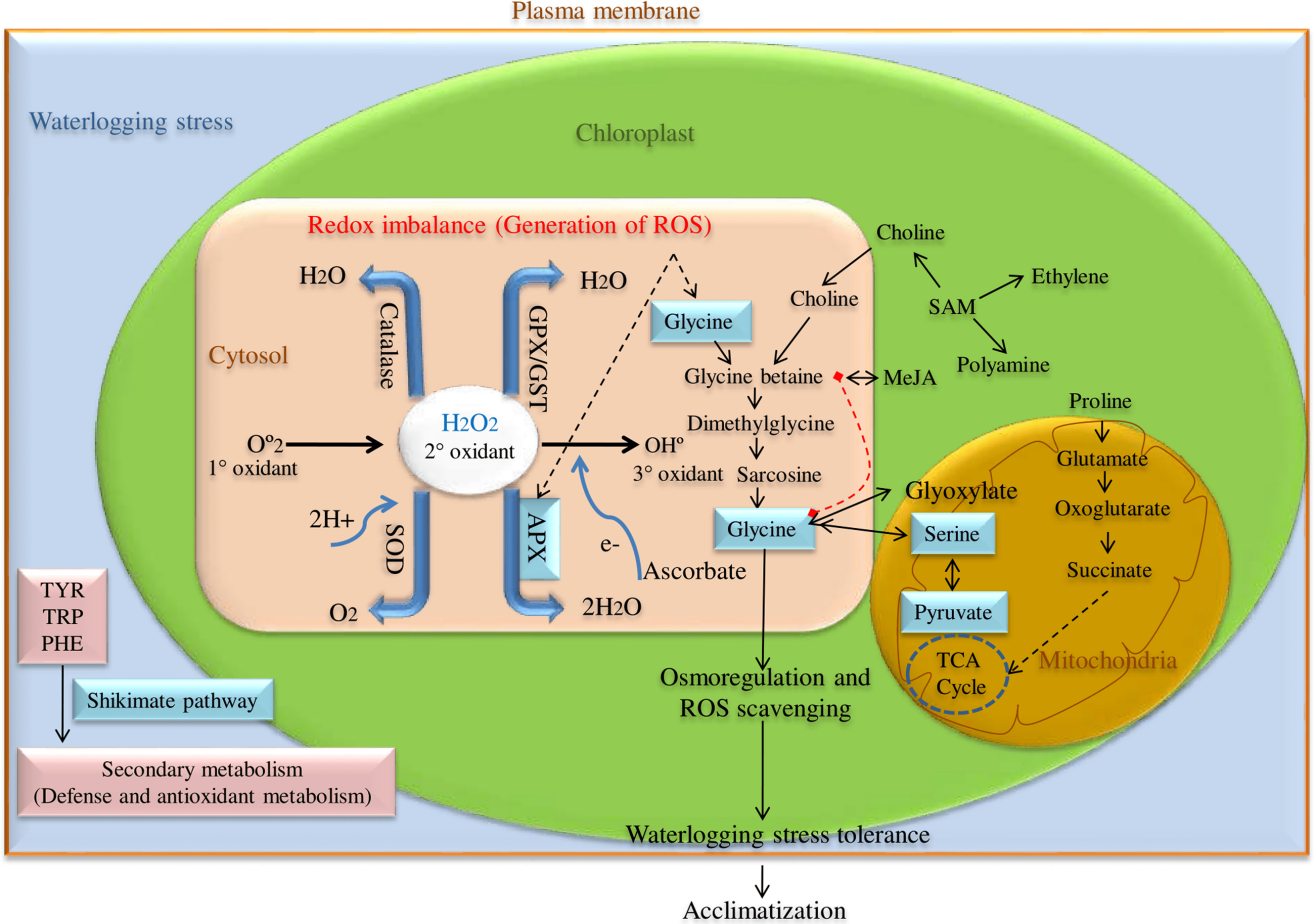
ROS scavenging pathway for waterlogging stress tolerance in pigeon pea.

ROS scavenging enzyme, particularly Ascorbate peroxidase (*APX*) is a key enzyme having a higher affinity for H_2_O_2_ and catalyzes H_2_O_2_ reduction to H_2_O using ascorbate as an electron donor in chloroplast, cytosol, and glyoxisomes as shown in Fig. 1. Among these, cytosolic *APX* has been well characterized in several plant species viz. *Nicotiana tabacum* (Hoque et al., 2012), *Arabidopsis thaliana* (Mittler & Zilinskas, 1993), *Spinacia oleracea* (Webb & Allen, 1995), and *Glycine max* (Dalton et al., 1996). Further, cytosolic *APX* activity has been shown to enhance in response to various abiotic stress like low and high water, salt, and temperature conditions (Park et al., 2004).

Besides, cytosolic *APX* has also been reported to regulate redox homeostasis in several plant species such as *A. thaliana* and *O. sativa*. Here, the cytosolic *APXs* play a very crucial role in detoxifying ROS via H_2_O_2_-scavenging reaction (Davletova et al., 2005; Pucciariello et al., 2012) and the absence or reduction of cytosolic *APX* promotes slow growth and retardation (Miller et al., 2007). Previous studies have revealed that any mutation in the cytosolic *APX* gene not only alters the normal growth and development, but also causes phenotypic abnormalities like semi-dwarf seedlings, leaf senescence, and yellowing of leaves (Bonifacio et al., 2011; Zhang et al., 2013). In Arabidopsis, the mutation of the cytosolic *APX1* gene makes it more sensitive to heat stress even in the presence of complete chloroplastic thylakoid *APX* gene, highlighting the potential role of cytosolic *APX1* during stress conditions. *APX1* enzyme has been observed to take on a critical function in executing defense role during various abiotic stresses (Koussevitzky et al., 2008).

This hypothesis has also been confirmed in this study by in silico studies in pigeon pea. Computational characterization of cytosolic isoform has been carried out by analyzing soybean *CytAPX* as template protein to the cloned *APX* gene (NCBI accession no. FJ914865.1 and FJ914864.1) from the waterlogging tolerant and sensitive pigeon pea genotypes. To identify the functional association of *CytccAPX* with H_2_O_2_, we performed a comprehensive and comparative homology modeling and molecular docking in contrasting pigeon pea genotypes. Since pigeon pea *CytccAPX* model structure is missing in the protein database (PDB), homology searches were performed, and the best top most homologous structure (PDB: 1OAF) was used as a reference structure for the production of *CytccAPX* 3D model structure via different bioinformatics servers. The predicted structure was further optimized using various simulation servers such as PROCHECK, ProSA, and Verify3D to replace missing atom loop optimization, structure validation, and energy minimization. The ligand (H_2_O_2_) binding affinity with the predicted model *CytccAPX* protein was interpreted by molecular docking analysis. This protein-ligand complex structure (*CytccAPX*-H_2_O_2_) was used to predict the binding site, binding affinity, and the number of conserved amino acids involved in the interaction, particularly under stress conditions.

## Materials and Methods

### Molecular and physiochemical profiling

The target (*APX*) amino acid sequence of two pigeon pea genotypes ICP 7035 (waterlogging sensitive) and ICPL 84023 (waterlogging tolerant) were used as a query for determining molecular and physiochemical profiling using ExPASy tool with ProtParam (https://web.expasy.org/protparam/) (Sairam, 2009). The solubility of these protein sequences was predicted by Predict Protein and trans-membrane protein prediction server MINNOU (https://minnou.cchmc.org/). Furthermore, the structural features were also determined using SOPMA (https://npsa-prabi.ibcp.fr/cgi-bin/npsa_automat.pl?page=/NPSA/npsa_sopma.html), PDBSum (http://www.ebi.ac.uk/thornton-srv/databases/pdbsum/Generate.html), and FindMod (https://web.expasy.org/findmod/). Prediction and analysis of sub-cellular localization, signal peptides, and antigenicity of these proteins were performed by PSORTb v3.0.2 (https://www.psort.org/psortb/), CELLO v2.5 (http://cello.life.nctu.edu.tw/), SignalP v4.1 (http://www.cbs.dtu.dk/services/SignalP-4.1/) and Immunomedicine group (http://imed.med.ucm.es/Tools/antigenic.pl) (Yu et al., 2006; Kolaskar & Tongaonkar, 1990; Yu et al., 2010).

### Phylogenetic analysis

Phylogenetic analysis was prepared using MEGAX software (Molecular Evolutionary Genetic Analysis) with maximum parsimony (default parameters) using Subtree-Pruning-Regrafting (SPR) algorithm for similarity search with other legumes (Kumar et al., 2018).

### Prediction of the 3-D structural models of the *CytccAPX* proteins from waterlogging tolerant and sensitive pigeon pea genotypes by comparative homology modeling analysis

**Figure 2:**
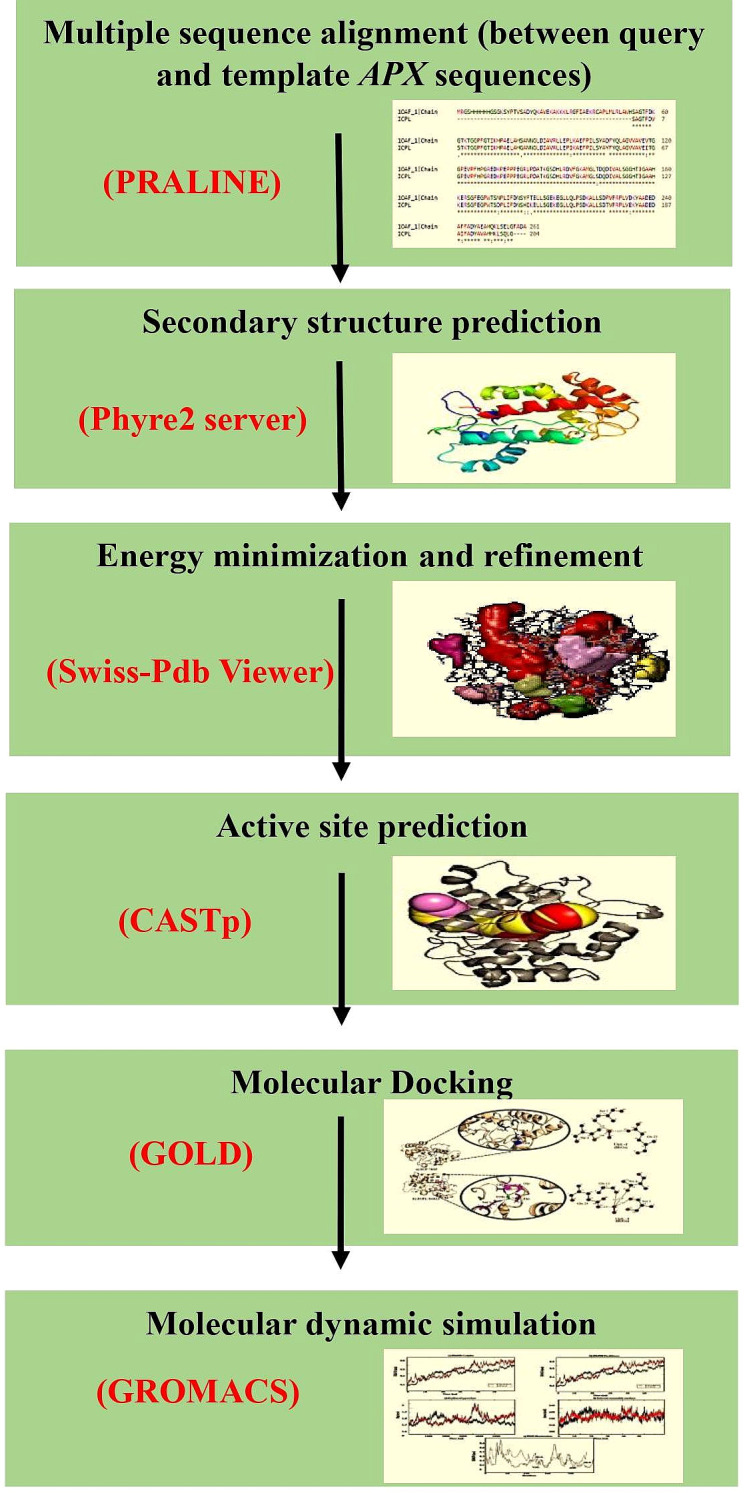
The workflow of the methodology used in molecular modeling, docking, and dynamic simulation.

The cytosolic *APX* protein sequences of ICPL 84023 and ICP 7035 were retrieved in FASTA format with accession no: ACQ99775.1 and ACQ99774.1 respectively. The homology modeling approach was used to predict the probable candidate as a template for both genotypes. Initially, BLASTp was performed against the structural database of PDB (http://www.rcsb.org/) on both the sequences to check whether any homologous structure is available or not. For modeling of the proteins, SWISS-MODEL (Waterhouse et al., 2018), a fully automated protein structure homology-modeling server that is accessible from the ExPASy web server and Phyre2 (Kelley et al., 2015), a Protein Homology/analogy Recognition Engine was used (intensive mode), since, both provide highly reliable outputs. Finally, their structures were aligned to check the RMSD value. The workflow of protein sequence retrieval and simulation analysis has been shown in Fig. 2.

### Energy minimization, superimposition, and validation

Energy minimization of the 3D structure was done on the predicted modeled structure utilizing the GROMOS96 43B1 force field executed in Swiss-PdbViewer (http://www.expasy.org/spdbv/) (Guex & Peitsch, 1997) followed by the superimposition of both the modeled structures with *G. max* using PyMOL software v1.7.4.5 to find Root Mean Square Deviation (RMSD). The modeled 3D structures of these proteins were visualized using PyMOL. Further, the quality of modeled structures was validated using various tools like ProSa (https://prosa.services.came.sbg.ac.at/prosa.php) (Wiederstein & Sippl, 2007) and SAVES v5.0 (https://servicesn.mbi.ucla.edu/SAVES/) which includes other servers like PROCHECK, Aqua, and Verify3D.The energy minimized structures were used to generate the Ramachandran plot, and its statistics give the information regarding the residues residing in the preferential regions, additional permitted regions, generously permitted regions, and disallowed regions also examined by residue-by-residue and overall geometry. Ramachandran Plot, which was offered by the PROCKECK system, ensured very strong faith for the protein that had been expected. There were 0% residues in the disallowed regions and only 2.5% of residues in the generously allowed regions. Howbeit, PROCHECK confirmed the reliability of the modeled protein structure. The SWISS-MODEL scores (QMEAN and GMQE) also provided additional reliability indicating that our model has good confidence and can be used for further studies.

### Pairwise 3D alignment and active site prediction

Pairwise 3D structure alignment of amino acid sequence of the template (1OAF) and final modeled query proteins was carried out using MATRAS 1.2 (http://strcomp.protein.osaka-u.ac.jp/matras/) (Kawabata, 2003). The active sites inside the proteins were predicted using CASTp v3.0 (http://sts.bioe.uic.edu/castp/index.html?2cpk) (Tian et al., 2018). It provides reliable pocket sites which consists of functional residues coincided as the potential protein binding sites in the ligand.

### Molecular docking studies

Molecular docking was done using the GOLD software suite (Jones et al., 1997), a protein-ligand docking software to identify the ligand-binding pockets and allosteric sites in protein 3D molecule.The interaction between protein-ligand was analyzed with PyMOL v1.7.4.5. (http://www.pymol.org/), and LIGPLOT v.4.5.3 (https://www.ebi.ac.uk/thornton-srv/software/LIGPLOT/) (Wallace, Laskowski & Thornton, 1995). The docking results were sorted according to the GOLD fitness score (GScore) and the complex with the highest score is considered to be the best docking complex and was considered for further analysis (Friesner et al., 2004). The GScore is calculated in Kcal/mol as:

\begin{eqnarray*}\text{G-Score = H bond+Lipo+Metal+Site+0:130 Coul+0:065 vdW-BuryP -RotB} \end{eqnarray*}

Where, H bond: Hydrogen bonds, Lipo: hydrophobic interactions, Metal: metal-binding term, Site: polar interactions in the binding site, Coul: columbic forces, vdW: Vander-Waals forces, Bury P: penalty for buried polar group, RotB: freezing rotable bonds.

### Molecular dynamics simulation

Molecular Dynamic (MD) simulations were performed on ICPL 84023-H_2_O_2_ and ICP 7035-H_2_O_2_ complexes in order to analyze their stability. All simulations were performed using the software GROMACS 5.1.5 (Abraham et al., 2015). Initially, to prepare the system for MD simulation, the protein-ligand complex was immersed in a dodecahedron-shaped box and solvated by the TIP3P water model. To prepare the topology of proteins CHARM36 all-atom force field was used (Huang & Mackerell, 2013). The H_2_O_2_ topologies were generated using CGenFF Server, which is an automated platform for ligand topology development (Vanommeslaeghe et al., 2009). Na^+^ counter ions were added to satisfy the electro-neutrality condition. Energy minimization was carried out for 50,000 steps using the steepest descent method. Thereafter, the system was subjected to position-restrained dynamic simulations under constant volume-constant temperature (NVT) and constant pressure-equilibrium (NPT) at a temperature of 300 K for 1,000 pico-second (ps) to extend the equilibrium. Finally, 50 nanoseconds (ns) MD simulations were performed on equilibrated systems for both the complexes. The output of MD analysis, such as trajectory files, was analyzed using RMSD, root mean square fluctuation (RMSF), the radius of gyration (Rg), and Solvent Accessible Surface Area (SASA).

### Analysis of molecular dynamics simulations

Structural dynamics of the ICPL 84023-H_2_O_2_ and ICP 7035-H_2_O_2_ complexes such as RMSD, RMSF, the radius of gyration (Rg), and SASA were taken from the trajectory files with the built-in function of GROMACS 5.1.5 and analyzed using *gmx rms, gmx rmsf, gmx gyrate* and *gmx sasa* respectively. The plots for 3D backbone model based on carbon-alpha (Cα) RMSD, RMSF of Cα, the radius of gyration, and SASA between both the complexes was generated to compare both the complexes using the GRaphing, Advanced Computation and Exploration (GRACE) program.

## Results and Discussion

### Molecular and physiochemical profiling of two pigeon pea proteins ICP 7035 and ICPL 84023

The molecular profile analysis revealed 192 and 204 amino acids in two pigeon pea proteins from ICP 7035 and ICPL 84023 respectively. The detailed analysis of two proteins based on molecular and physiochemical profiling has been shown in Table S1. The grand average of hydropathicity (GRAVY) index was measured to be −0.271 and −0.254 in ICP 7035 and ICPL 84023 respectively showing that both proteins are hydrophilic and further confirmed by Kyte and Doolittle hydropathy plot as shown in Fig. S1 and Table S2.

The function of these proteins was predicted using Predict Protein (https://www.predictprotein.org/), a stand-alone version of MINNOU (https://minnou.cchmc.org/) and CELLO v2.5 (http://cello.life.nctu.edu.tw/) servers. The result anticipated that both the proteins being intracellularly localized suggesting both the sequences were soluble protein and no trans-membrane domain was found under stress condition as shown in Fig. S2. The secondary structure composition of ICP 7035 *APX* protein contains Loop (64.06%), Helix (31.25%), and Strand (4.69%) with solvent accessibility distribution as exposed (67.19%), buried (26.04%), and intermediate (6.77%). Sub-cellular localization of eukaryotic domain predicted it as a cytoplasmic class (GO:0005737) protein with a confidence score 90, and CELLO prediction reliability 3.270. The protein-binding reliability score was found to be 81 (GO:0005515). However, secondary structure composition of ICPL 84023 *APX* protein contains Loop (61.76%), Helix (35.29%), and Strand (2.94%) with solvent accessibility distribution as exposed (47.55%), buried (38.73%) and intermediate (13.73%). Sub-cellular localization of the eukaryotic domain predicted it as a cytoplasmic class (GO:0005737) protein with confidence score 88, and CELLO prediction reliability 3.334. The protein-binding reliability score was found to be 80 (GO:0005515). No signal peptide sequences was found in ICP 7035 and ICPL 84023 by SignalP 5.0 server (http://www.cbs.dtu.dk/services/SignalP/).

The result of the predicted antigenic peptide tool suggests ICP 7035 and ICPL 84023 contain a total of 8 and 10 antigenic determinants with an average antigenic resistance of 1.0265 and 1.0323, respectively as shown in Fig. S3 and Table 1. This may be due to the fact ICPL 84023 consists of additional amino acids (i.e., 204) and greater molecular weight (i.e., 21792.47 kDa) compared with ICP 7035 (192 and 20518.97 kDa). Analysis of proteins by Pfam suggests both ICPL 84023 (accession number ACQ99775.1) and ICP 7035 (accession number ACQ99774.1) consist of one peroxidase domain (PF00141) of Class III monomeric glycoprotein containing two calcium ions and four intact disulfide bridges, having multiple tissue-specific functions including cell wall and hormone biosynthesis, defense, removal of H_2_O_2_, and toxic compounds by oxidation reaction from chloroplasts and cytosol.

**Table 1:** Distribution of antigenic determinants in two pigeon pea proteins.

**S.No.**	**Start position**	**Sequence**	**End position**	**S.No.**	**Start position**	**Sequence**	**End position**
**ICP 7035**				**ICPL 84023**			
**1**	19	GTIKHPAEL	27	**1**	4	LSAGTFDV	11
**2**	35	LDIAVRLLEPIKAEFP ILSYADFYQLAGVV AVEI	68	**2**	20	GTIKHPAEL	28
**3**	70	GGPEVPF	76	**3**	36	LDIAVRLLEPIKAEFPI LSYAYFYQLAGVVA VEI	69
**4**	101	HLRDVFG	107	**4**	71	GGPEVPF	77
**5**	113	SDQDIVALSG	122	**5**	102	HLRDVFG	108
**6**	141	TSDPLIFD	148	**6**	114	SDQDIVALSG	123
**7**	151	HFKELLS	157	**7**	142	TSDPLIFD	149
**8**	161	EGLLQLPSDKALLSD PVFRLLVEK	184	**8**	152	HIKELLS	158
				**9**	162	EGLLQLPSDKALLSD TVFRPLVEK	185
				**10**	193	IFADYAVAHHKL	204

### Phylogenetic analysis of *APX* gene from different legumes

**Figure 3:**
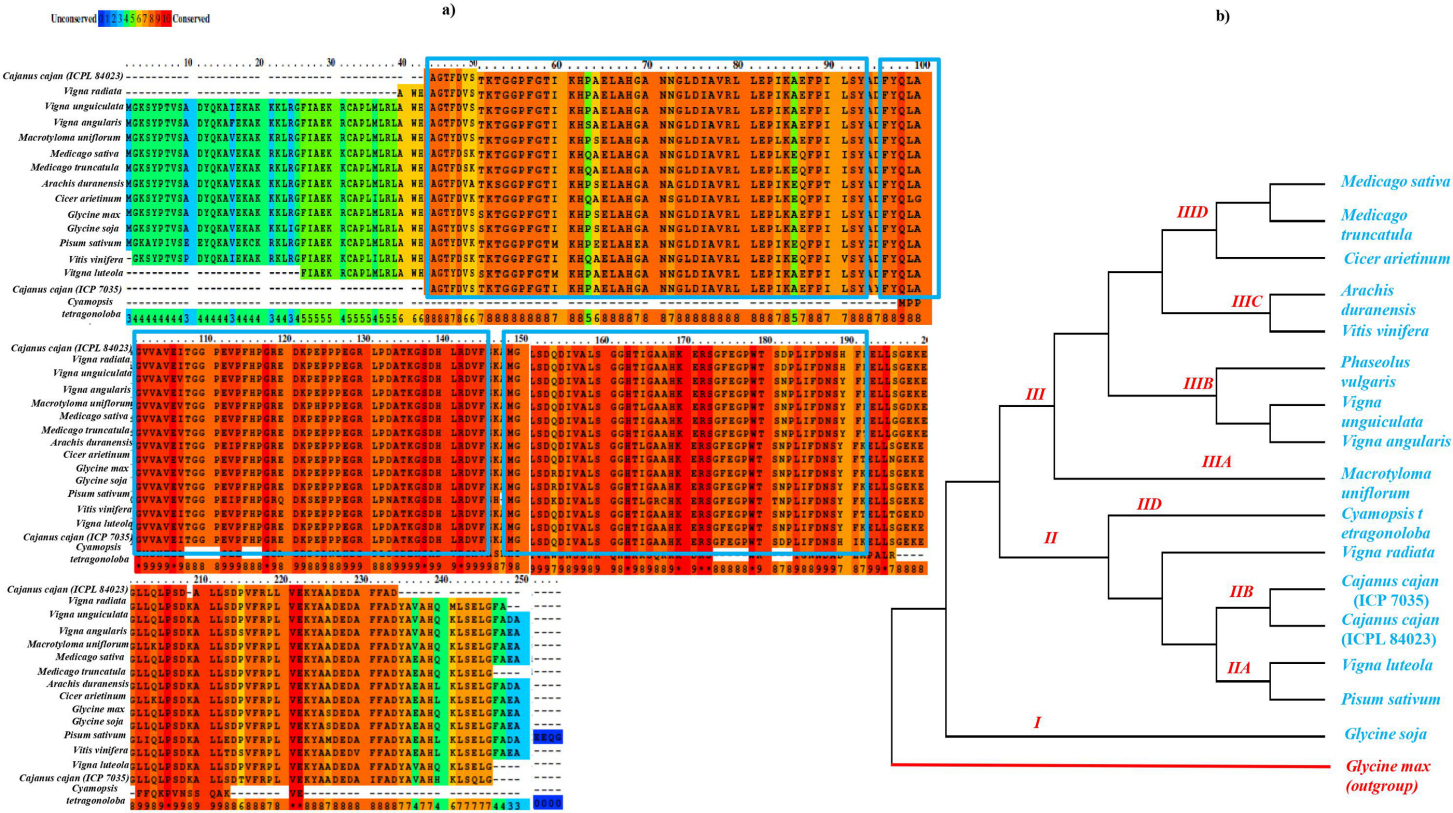
(A) Sequence similarity and (B) phylogenetic analysis of *APX* gene in 16 different legume species. The blue rectangle box represents the highly conserved *APX* domain.

The sequence similarity based on conserved amino acid sequence among sixteen different legume species including 11 genus for *APX* gene (*Glycine max:* NP_001235587.1, *Glycine soja:* XP_028194531.1 *, Macrotyloma uniflorum:* AFN21424.1 *, Vigna radiata:* ACD44387.1 *, Vigna angularis:* XP_017433572.1 *, Vigna unguiculata:* XP_027910468.1 *, Cicer arietinum:* XP_004505943.1 *, Medicago sativa:* AIY27528.1 *, Medicago tranculata:* KEH30074.1 *, Arachis duranensis:* XP_015952343.1 *, Vitis vinifera:* A9UFX7*, Cajanus cajan* ICP 7035*:* ACQ99774.1; ICPL 84023: ACQ99775.1 *, Pisum sativum:* P48534 *, Phaseolus vulgaris:* XP_007132164 *, Cyamopsis tetragonoloba,* and *Vigna lutela:* ACD44386.1) was done using both PRALINE multiple sequence alignment tool (http://ibi.vu.nl/programs/pralinewww/) and Multiple Em for Motif Elicitation (MEME) as shown in Fig. 3A. The well characterized *G. max APX* gene family was used as a reference for the preparation of phylogenetic tree for *CcAPX* along with other legume species. The phylogenetic analysis performed using MEGAX revealed that *APX* gene family clustered into broadly three main Clades I, II and III which is further bifurcated into IIA, IIB, IIC, IID, IIIA, IIIB, IIIC and IIID clades based on the sequences that are quite similar and limited to small numbers of sequences. The evolutionary relationship between these species was done using the Maximum Parsimony (MP) obtained using the Subtree-Pruning-Regrafting (SPR) algorithm, with consistency index (0.964206), the retention index (0.912088), and the composite index (0.879440) for all sites (Fig. 3B). Among 16 legume species, *C. tetragonoloba APX* gene (Table S3) was found to be similar with *G.max* and *C. cajan* species.

### Molecular modeling of *APX* gene in tolerant and sensitive pigeon pea genotypes

For ICP 7035, the top template (PDB: 1OAF, 3ZCH, 3ZCG, 3ZCY) in the BLAST results had 94.27% identity and 100% query coverage and similarly, for ICPL 84023, the top template (PDB: 1OAF, 3ZCH, 3ZCG, 3ZCY) in the BLAST results had 91.67% identity and 100% query coverage, hence indicating that homology modeling will be an ideal approach in both cases. The RMSD of the output structures for ICP 7035 from SWISS-MODEL and Phyre2 was 0.28 A° and for ICPL 84023 was 0.26 A° indicating that the output from both the tools is almost similar. Chloroplastic ascorbate peroxidase protein of *Glycine max* (PDB:1OAF) had the highest homologous structure and was selected for comparative modeling. The GMQE score for both the proteins was found to be 0.98 which indicates that the output structure is highly reliable. The Q-Mean scores were 0.86 and 0.90 for ICP 7035 and ICPL 84023, respectively which highlights good quality structures. In the Phyre2 results, 99% of residues of ICP 7035 were modeled at >90% confidence and 100% residues of ICPL 84023 were modeled at >90% confidence. The *CytccAPX* protein of both the genotypes thus generated from Phyre2 was considered for the active site recognition and substrate docking (H_2_O_2_) with the enzyme.

**Figure 4:**
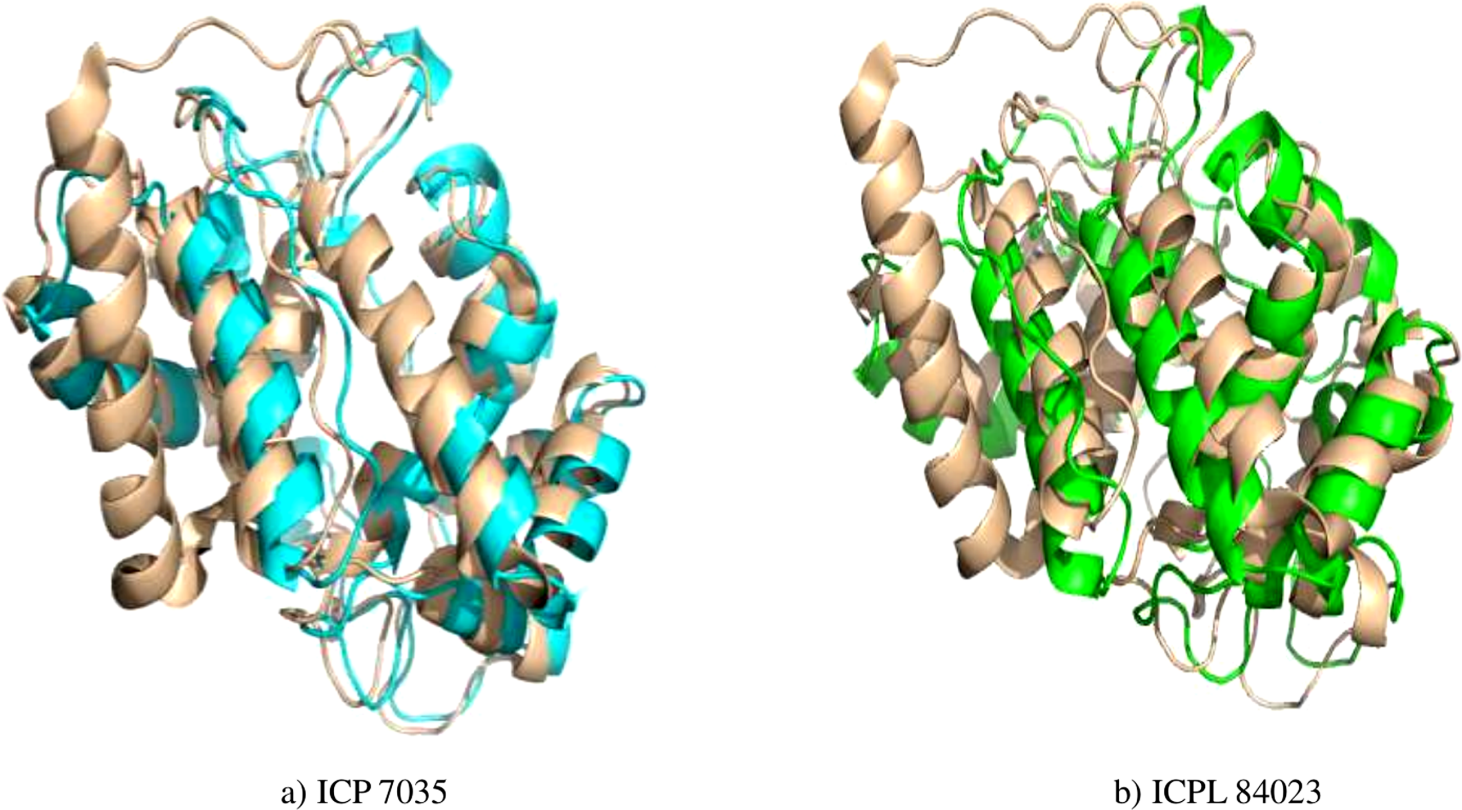
Superimposed structure of energy minimized template (*ccAPX*) of ICP 7035 shown in blue colour and ICPL 84023 shown in green colour with homologous sequence of soybean (1OAF: in cream colour).

### Structure optimization (EM), superimposition of structure, and validation

Energy minimization measured by the mean distance of superimposed proteins between the backbones of ICP 7035 and ICPL 84023 was done using Swiss-PDB Viewer. The modeled structures were found to have RMSD values less than 1 A° in both the genotypes i.e., 0.288 A° (between 1,140 atoms) and 0.261 A° (1,154 atoms) after superimposition of energy minimized structure with *Glycine max* (1OAF) in ICP 7035 and ICPL 84023, respectively (Fig. 4).

**Figure 5:**
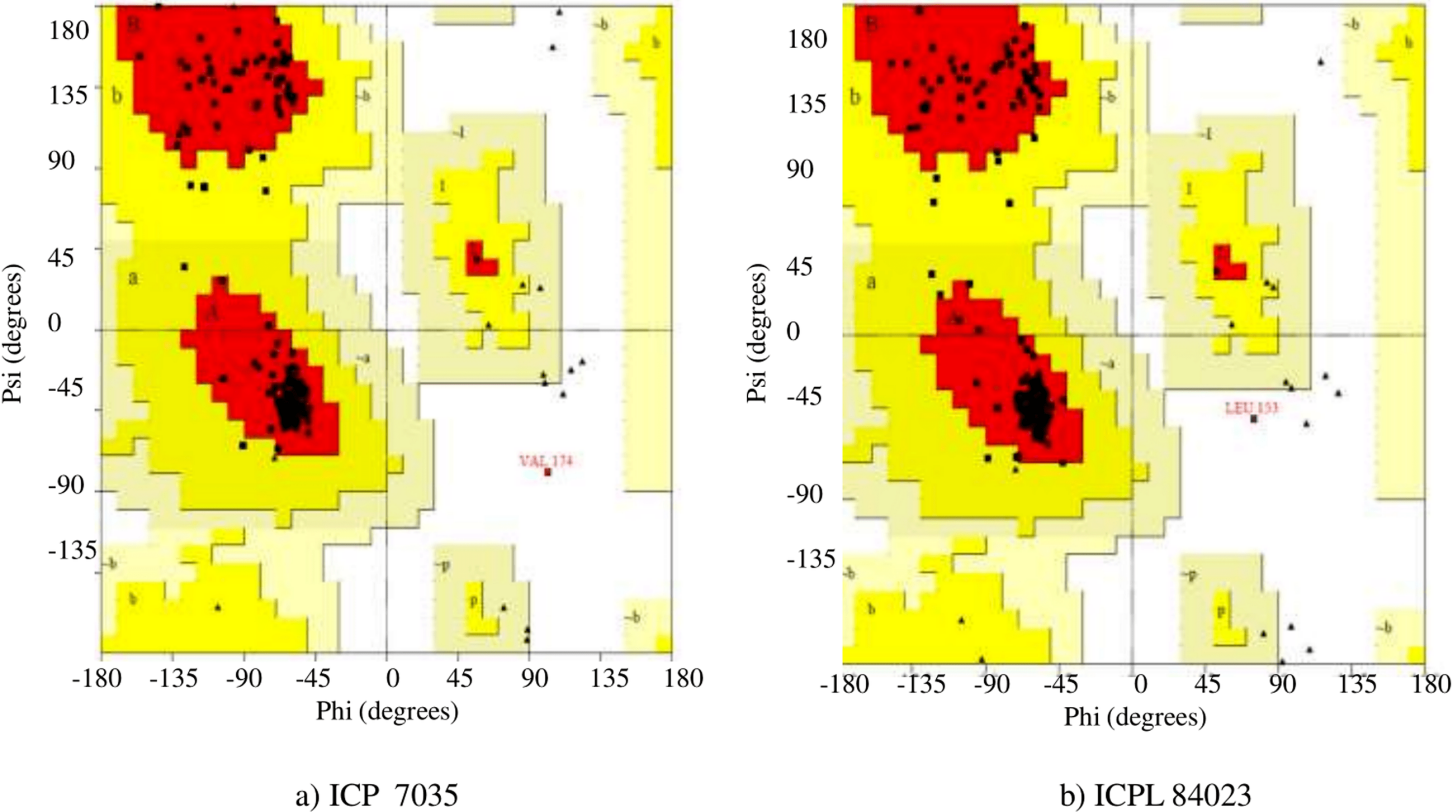
Ramachandran plot of the homology based model of (A) ICP 7035 and (B) ICPL 84023.

In the Ramachandran plot study, the residues were graded in the quadrangle (Psi/Phi) according to their regions as shown in Fig. 5. The results revealed that the allowed areas residues were 93.3% and 95.4%, respectively with detailed stereochemical properties shown in Table S4. The distribution of bond lengths and bond angles of the main chain for these proteins was found to be within the limits shown in Fig. S4. These figures assigned by the Ramachandran plot reflect the good quality of the models predicted and worth investigating further. The overall quality factor was further conformed by ERRAT, ProSA, and PROVE and found to be satisfactory with Z-score −5.73 and −6.01, thus proving the constructed model to be valid for active site prediction and docking of ligand (H_2_O_2_) with *ccAPX* proteins of ICP 7035 and ICPL 84023 (Fig. S5). Hence, it proved that a similar structure may exist in nature.

### Pairwise 3D alignment and active site identification of model protein *CcAPX*

**Figure 6:**
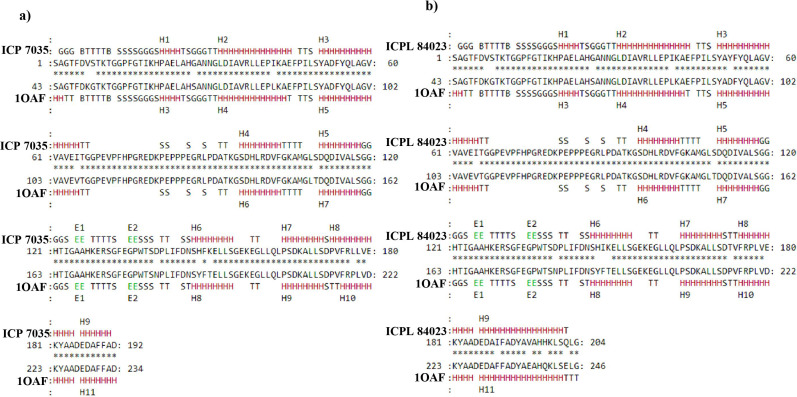
Pairwise 3D secondary structure alignment between the template (1OAF) and the model protein *ccAPX* of ICP 7035 and ICPL 84023 using MATRAS. Alignment showing secondary structure identity (A) 96.9% for ICP 7035 and (B) 98.5% for ICPL 84023 (red color represents conserved secondary structure).

The secondary structure of final modeled query proteins (ICP 7035 and ICPL 84023) and the template protein (1OAF) were also compared by pairwise 3D alignment using MATRAS 1.2 as shown in Fig. 6. The structure of the template and the final model protein *ccAPX* of ICP 7035 was found to have 94.3% and 96.9% sequence and secondary structure identity while 91.7% and 98.5% sequence and secondary structure identity was predicted for ICPL 84023 respectively. These results revealed that the final structures were highly reliable and suitable for further active site identification. The detailed secondary structure prediction analysis including summary, number of the beta-sheet, helices, turns and strand, beta hairpin, helix-helix interaction and gamma turn of both ICP 7035, and ICPL 84023 with their structural view has been described in Table S5 and Fig. S6.

The final protein model was used for the identification of amino acid residues present in the active site pocket using CASTp v3.0. Possible binding site for template protein (1OAF) was also predicted using the same methodology. The detail of active site residues has been mentioned in Table 2. The comparative studies revealed that residues ALA, ARG, ASN, ASP, GLN, GLU, GLY, HIS, ILE, LEU, LYS, MET, PHE, PRO, SER, THR, TRP, TYR, and VAL were highly conserved in active site of both the model proteins and the template protein (1OAF) and thus, it could be predicted that they would share similar biological function.

**Table 2:** Active site residues identified in two pigeon pea genotypes.

**Active Sites for ICP 7035**	**Active Sites for ICPL 84023**
SER1, ALA2, GLY3, THR4, PHE5, ASP6, VAl7, SER8, LYS10, THR11, GLY12, GLY13, PRO14, PHE15, GLY16, THR17, ILE18, LYS19, HIS20, GLU23, HIS26, GLY27, ALA28, ASN29, ASN30, GLY31, LEU32, ILE34, ALA35, VAL36, LEU38, Leu39, GLU40, ILE42, LYS43, LEU49, TYR51, ALA52, ASP53, PHE54, TYR55, GLN56, LEU57, ALA58, GLY59, VAL60, VAL61, ALA62, VAL63, GLU64, ILE65, THR66, PRO69, GLU70, VAL71, PRO72, PHE73, PRO75, ARG77, ASP79, LYS80, PRO81, GLU82, PRO84, PRO85, GLU86, GLY87, ARG88, LEU89, PRO90, ASP91, ALA92, LYS94, GLY95, HIS98, LEU99, ARG100, VAL102, PHE103, GLY104, LYS105, ALA106, MET107, LEU109, SER110, Asp111, Gln112, ASP113, ILE114, ALA116, LEU117, SER118, GLY120, HIS121, THR122, ILE123, GLY124, ALA125, ALA126, HIS127, ARG130, SER131, PHE133, TRP137, THR138, ASP140, PRO141, LEU142, ILE143, PHE144, ASP145, SER147, HIS148, GLU151, LEU152, GLY155, GLU156, LYS157, LEU160, LEU161, GLN162, LEU163, SER165, ASP166, LEU169, PHE189, PHE190, ASP192	SER1, ALA2, GLY3, THR4, PHE5, ASP6, VAL7, SER8, LYS10, THR11, GLY12, GLY13, PRO14, PHE15, GLY16, THR17, ILE18, LYS19, HIS20, GLU23, HIS26, GLY27, ALA28, ASN29, ASN30, GLY31, LEU32, ILE34, ALA35, VAL36, LEU38, LEU39, GLU40, ILE42, LYS43, TYR51, ALA52, PHE54, TYR55, GLN56, LEU57, ALA58, GLY59, VAL60, VAL61, ALA62, VAL63, GLU64, ILE65, THR66, PRO69, GLU70, VAL71, PHE73, ARG77, ASP79, LYS80, PRO81, GLU82, PRO84, PRO85, GLU86, GLY87, ARG88, LEU89, PRO90, ASP91, ALA92, LYS94, GLY95, HIS98, LEU99, ARG100, VAL102, PHE103, GLY104, LYS105, ALA106, MET107, LEU109, SER110, ASP111, GLN112, ASP113, ILE114, ALA116, LEU117, SER118, GLY119, GLY120, HIS121, THR122, ILE123, GLY124, ALA125, ALA126, HIS127, ARG130, SER131, PHE133, TRP137, THR138, ASP140, PRO141, LEU142, ILE143, PHE144, ASP145, ASN146, SER147, HIS148, ILE149, GLU151, LEU152, GLY155, GLU156, LYS157, LEU160, LEU161, GLN162, LEU163, SER165, ASP166, LEU169, ILE189, PHE190, ASP192, TYR193, VAL195, ALA196, HIS197, HIS198, LEU200, SER201, GLN202, LEU203, GLY204

Various studies have been shown that the wide number of proline residues under abiotic stress conditions lead to the accumulation of higher ROS production implicating in the hypersensitivity of plants (Liang et al., 2013). It is also reported that higher proline level act as a chaperone which prevents protein aggregation and thermo-denaturation and helps in plant protection against oxidative stress. Besides, it is also involved in stabilizing redox enzymes, including *SOD, CAT, GSH,* and *APX* (Xu, Yin & Li, 2009; Kumar et al., 1998; Islam et al., 2009a; Islam et al., 2009b; Chen & Dickman, 2005; Hoque et al., 2008; Hoque et al., 2007). While the presence of tyrosine residues in ICPL 84023 indicate the flow of the TCA cycle, which can further act as a precursor for the accumulation of the secondary metabolite pathways involved in stress tolerance. In this study, we also found the high content of proline residues in ICP 7035 as compared to ICPL 84023 indicating some kind of contribution to the sensitive and tolerant behavior of plants under waterlogging stress conditions. Various in vitro studies based on physiological and biochemical parameters also confirmed that higher expression of *APX* in pigeon pea genotypes ICPL 84023 and ICP 301 contributing to making the plant tolerant to waterlogging stress (Sairam, 2009; Kumutha et al., 2009).

### Molecular docking

The ligand H_2_O_2_(CID=784) was obtained from the PubChem database, NCBI (https://pubchem.ncbi.nlm.nih.gov/compound/Hydrogen-peroxide). Energy minimized 3D structures of ICP 7035 and ICPL 84023 were used as the target proteins with potential H_2_O_2_ ligand to dock into protein active sites using GOLD software suite with default functions which are based on genetic algorithm runs was used for all computation and the ligand saved a range of 10 solutions. Genetic Optimization for Ligand Docking (GOLD) is useful software to identify the best compound by optimizing their topographical and chemical complementarity and scoring their interaction dependent on kcal/mol binding compatibility (Jones et al., 1997; Nissink et al., 2002). The final docked structure obtained from two genotypes of pigeon pea was determined depending on hydrogen bonds formed and the bond distance between the active site and inhibitor atomic coordinates. The gold scores for each structure using H_2_O_2_ as ligand has been shown in Table 3.

**Table 3:** Gold score values for ICP 7035 and ICPL 84023 with GOLD rank.

	**ICP 7035**	**ICPL 84023**
** Gold Score kcal/mol (GOLD rank)**	16.7423 (1)	23.3952 (1)
	16.6919 (2)	23.1180 (2)
	16.5020 (3)	22.7178 (3)
		21.7407 (4)
		21.7120 (5)

**Figure 7:**
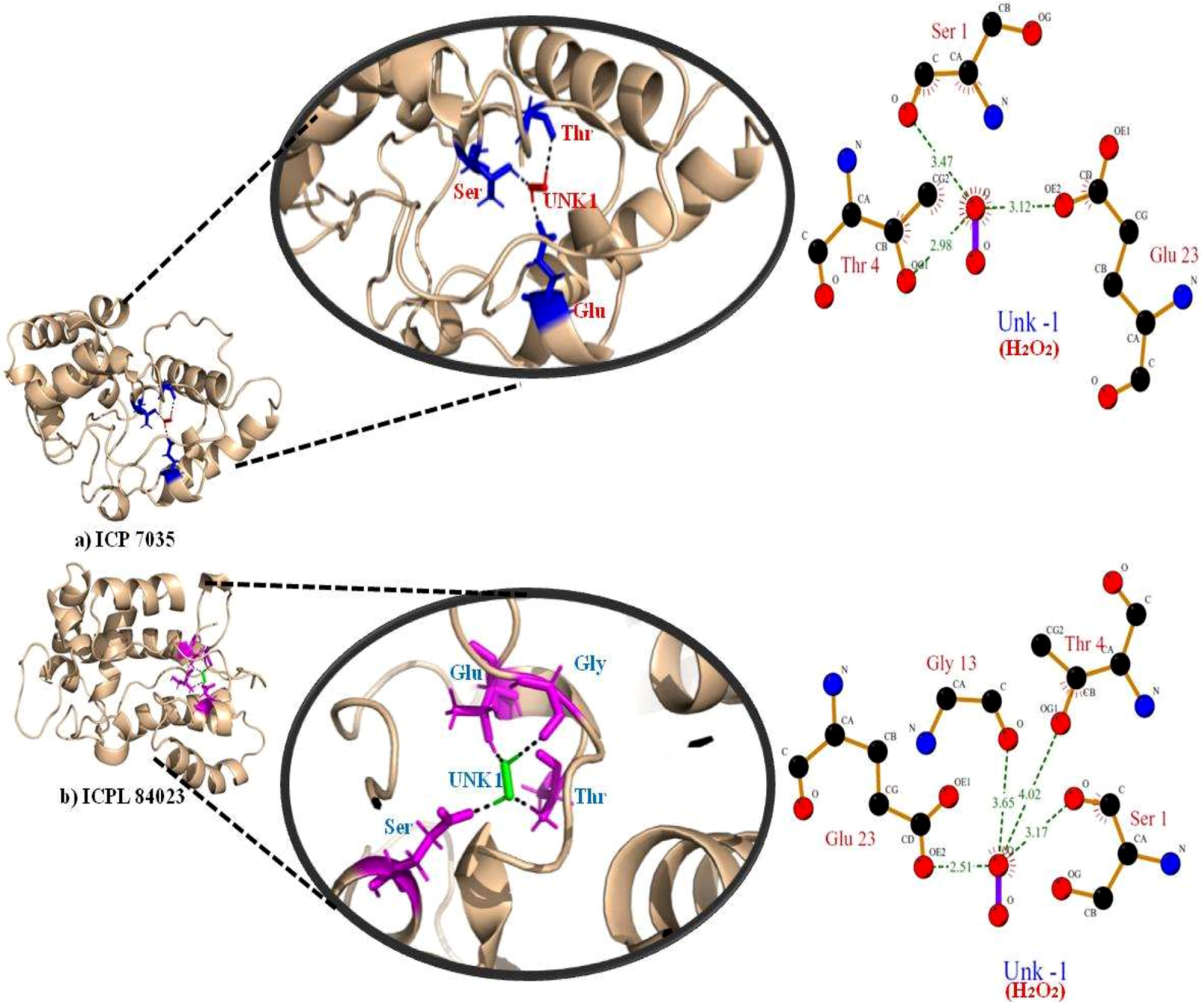
Docking of *APX* gene with H_2_O_2_ in two pigeon pea genotypes (A) ICP 7035 and (B) ICPL 84023.

The highest gold score value describing the higher binding energy was taken to visualize the final 3D structure docking with H_2_O_2_ ligand using PyMol has been shown in Fig. 7. The Ligplot analysis shows that ICP 7035 is bounded with three amino acid residues Ser1, Thr4, and Glu23 while ICPL 84023 is bounded with four residues Ser1, Thr4, Gly13, and Glu23 through h-bond interaction with docked ligand H_2_O_2_. The docked complex of ICPL 84023 showed the best G-score (23.39 kcal/mol) in comparison to ICP 7035 (16.74 kcal/mol) depicting the higher production of *APX* for scavenging ROS production making this genotype more tolerant. This is due to the extra Glycine residue present in tolerant pigeon pea genotype ICPL 84023. Various studies have been shown that glycine and choline are the two key regulatory residues in glycinebetaine biosynthesis pathway playing important role in osmoregulation (Xu et al., 2018; Annunziata et al., 2019). All the hydrogen bonds along with their distance between the ligand and the proteins with the active site of the protein are shown in Table 4.

**Table 4:** Number of hydrogen bonds in ICP 7035 and ICPL 84023 (*ccAPX*) along with their distance between the H_2_O_2_ ligand and active site residues.

**Genotypes**	**Peroxidase enzyme**		**H**_2_**O**_2_ atom	Distance (A^∘^)
	**Residue**	**Atom**		
**ICP 7035**	SER (1)	O (4)	O (−1)	3.47
	THR (4)	OG1(36)	O (−1)	2.98
	GLU (23)	OE2(313)	O (−1)	3.12
**ICPL 84023**	SER (1)	O (4)	O (−1)	3.17
	THR (4)	OG1(36)	O (−1)	4.02
	GLY (13)	O (164)	O (−1)	3.65
	GLU (23)	OE2 (313)	O (−1)	2.51

### Simulation analysis

The stability of both complexes was evaluated using RMSD values of the backbone atoms through MD simulation run of 50 ns. The trajectory analysis shows that the complexes attained equilibrium for the protein backbone atoms. The structure properties, such as RMSD of the backbone and Cα atoms were calculated for ICPL 84023-H_2_O_2_ and ICP 7035- H_2_O_2_ complexes as a function of time. The average RMSD for backbone atoms in ICPL 84023-H_2_O_2_ and ICP 7035-H_2_O_2_ were found to be 0.419 nm and 0.481 nm, respectively (Table 5). The plot of radius of gyration, Rg (protein) vs time varied between 1.687 nm and 1.875 nm for ICPL 84023-H_2_O_2_ whereas 1.675 nm and 1.964 nm for ICP 7035-H_2_O_2_. The average radius of gyration (protein) of ICPL 84023-H_2_O_2_ and ICP 7035-H_2_O_2_ were 1.765 nm and 1.778 nm, respectively. The SASA of ICPL 84023-H_2_O_2_ varied from ∼132 nm^2^ to ∼105 nm^2^(average 132.827 nm^2^); and that of ICP 7035-H_2_O_2_ from ∼133 nm^2^ to ∼108 nm^2^ (average 133.861 nm^2^), during the entire run ranging from 0-50 ns (Table 5).

**Table 5:** Time averaged structural properties of ICPL 84023-H_2_O_2_ and ICP 7035-H_2_O_2_ complexes at 50 ns.

Properties	**ICPL 84023-H**_2_O_2_ complex	ICP 7035-H_2_O_2_ complex
C*α* RMSD (nm)	0.426	0.488
Backbone RMSD (nm)	0.419	0.481
Radius of Gyration(nm)	1.765	1.778
SASA (nm^2^)	132.827	133.861
RMSF	0.233	0.290

**Figure 8:**
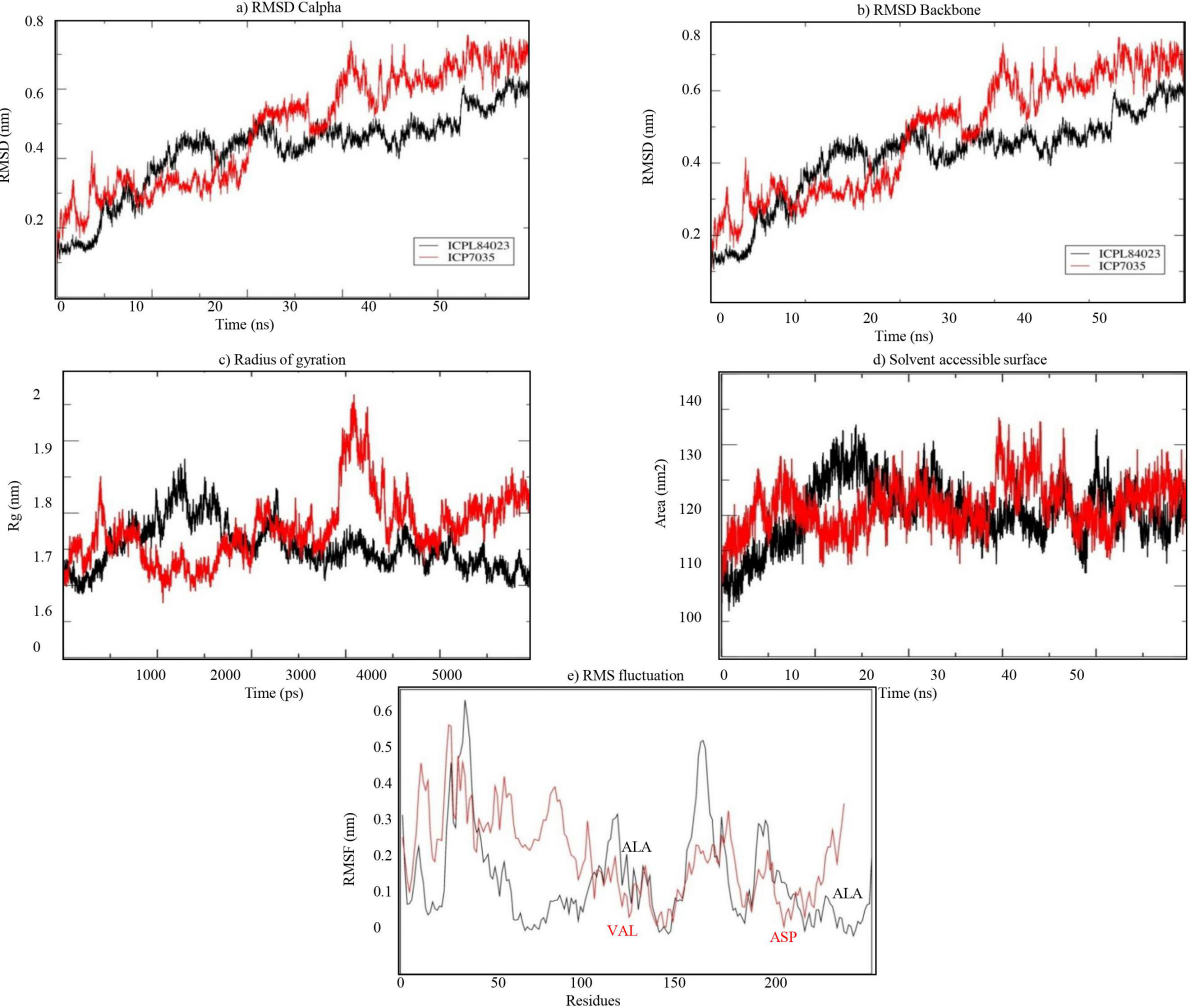
Structural properties of ICPL 84023-H_2_O_2_ complex (black line) and ICP 7035-H_2_O_2_ complex (red line): (A) CαRMSD of both the complexes; (B) backbone RMSD of both the complexes; (C) Radius of gyration of proteins; (D) solvent.

The RMSF analysis of ICPL 84023-H_2_O_2_ complex specifies that ALA196 and ALA116 are amongst the least fluctuated or flexible residues while in ICP 7035-H_2_O_2_ complex, VAL115, and ASP166 have revealed the least fluctuation in comparison to other residues (Fig. 8E). The important binding residues in ICPL 84023-H_2_O_2_complex (SER1,THR4, GLU23, and GLY13) have shown less fluctuation than ICP 7035-H_2_O_2_ complex (SER1, THR4, and GLU23). In the docking study, these residues have also given their major role in binding the ligand site. Thus, their interactions with H_2_O_2_may be attributed to the limited flexibility of these residues. Overall, residues in the ICP 7035-H_2_O_2_ complex have shown relatively more fluctuations than the ICPL 84023-H_2_O_2_ complex. The plots for RMSD, RMSF, and the radius of gyration (Rg) indicated that ICPL 84023 complex was compact with stable protein folds during MD simulation leading to more stability than ICP 7035 (Fig. 8).

## Conclusion

Waterlogging is one of the significant challenges in the global pigeon pea production with little molecular information on the key genes such as *APX*. The detailed characterization of the molecular and physiochemical properties of *APX* proteins under waterlogging sensitive and tolerant conditions including their 3D structure, antigenicity, structural and functional properties, will be informative and may contribute towards identifying the function of the *APX* in the exact stress tolerance mechanism. Here, we have identified the binding efficiency of ligand H_2_O_2_with *ccAPX* by docking study and hypothesized that glycine residue could be the reason for contributing to better waterlogging stress tolerance in ICPL 84023 genotype. Besides, MD simulation analysis also highlighted the role of this residue in molecular recognition. The glycine residue identified in this study could be the reason for controlling the production of glycinebetaine pathway, which can ameliorate the inhibitory effect of waterlogging stress in pigeon pea. The results obtained in this study can be used as a reliable path for future metabolic and gene editing studies targeting several genes in glycinebetaine biosynthetic pathway for generating enhanced waterlogging tolerance in pigeon pea.

##  Supplemental Information

10.7717/peerj.10888/supp-1 Supplemental Information 1 Kyte and Doolittle hydropathy plot in a) ICP 7035 and b) ICPL 84023

10.7717/peerj.10888/supp-2 Supplemental Information 2 Transmembrane domain structure prediction in (a) ICP 7035 and (b) ICPL 84023

10.7717/peerj.10888/supp-3 Supplemental Information 3 Average antigenic propensity in (a) ICP 7035 and (b) ICPL 84023

10.7717/peerj.10888/supp-4 Supplemental Information 4 Structural view of helix, beta turn, gamma turn and distorted geometry in ICP 7035 and ICPL 84023

10.7717/peerj.10888/supp-5 Supplemental Information 5 Ramachandran plots of all residue types, main chain bond length, main chain properties, side chain properties, residue properties, RMS distance from planarity and chi1-chi2 plots in ICP 7035 and ICPL 84023

10.7717/peerj.10888/supp-6 Supplemental Information 6 Verify 3D, ERRAT, SAVES, PROVE and ProSA results of ICP 7035 and ICPL 84023

10.7717/peerj.10888/supp-7 Supplemental Information 7 Molecular and physiochemical properties of *APX* protein in two pigeon pea genotypes

10.7717/peerj.10888/supp-8 Supplemental Information 8 Individual hydropathy index of all 20 amino acids using the Hphob./Kyte& Doolittle scale in ICP 7035 and ICPL 84023

10.7717/peerj.10888/supp-9 Supplemental Information 9 Cytosolic APX sequence of *Cymopsis tetragonoloba* (unpublished data)

10.7717/peerj.10888/supp-10 Supplemental Information 10 Detailed secondary structure in ICP 7035 and ICPL 84023 showing a) Secondary structure summary b) beta sheet c) beta hairpins, d) beta strands, e) helices, f) helix-helix interaction, g) beta turns, h) gamma turns

10.7717/peerj.10888/supp-11 Supplemental Information 11 Summary of steriochemical properties of ICP 7035 using PROCHECK

## Abbreviations

APX: Ascorbate peroxidase

BLAST: Basic Local Alignment Search Tool

CAT: Catalase

CCP-like: *Cytochrome c peroxidase*

GPX: Glutathione peroxidase

H_2_O_2_: Hydrogen peroxide

MD: Molecular dynamics

NOS: Nitric oxide synthase

ns: nano second

O${}_{2}^{-}$: Superoxide

ps: pico second

RMSD: Root Mean Square Deviation

RMSF: Root Mean Square Fluctuation

Rg: Radius of Gyration

ROS: Reactive oxygen species

RNS: Reactive nitrogen species

SASA: Solvent Accessible Surface Area

SOD: Superoxide dimutase

TCA cycle: Tricarboxylic acid cycle

## Additional Information and Declarations

### Data Availability

The following information was supplied regarding data availability: Raw data are available in the Supplemental Files.
