# Supplementary figures and images for: In silico characterization and homology modeling of cytosolic APX gene predicts novel glycine residue modulating waterlogging stress response in pigeon pea
Source: PeerJ. 2021 May 12;9:e10888. doi: 10.7717/peerj.10888 (PMC8123230; doi:10.7717/peerj.10888)

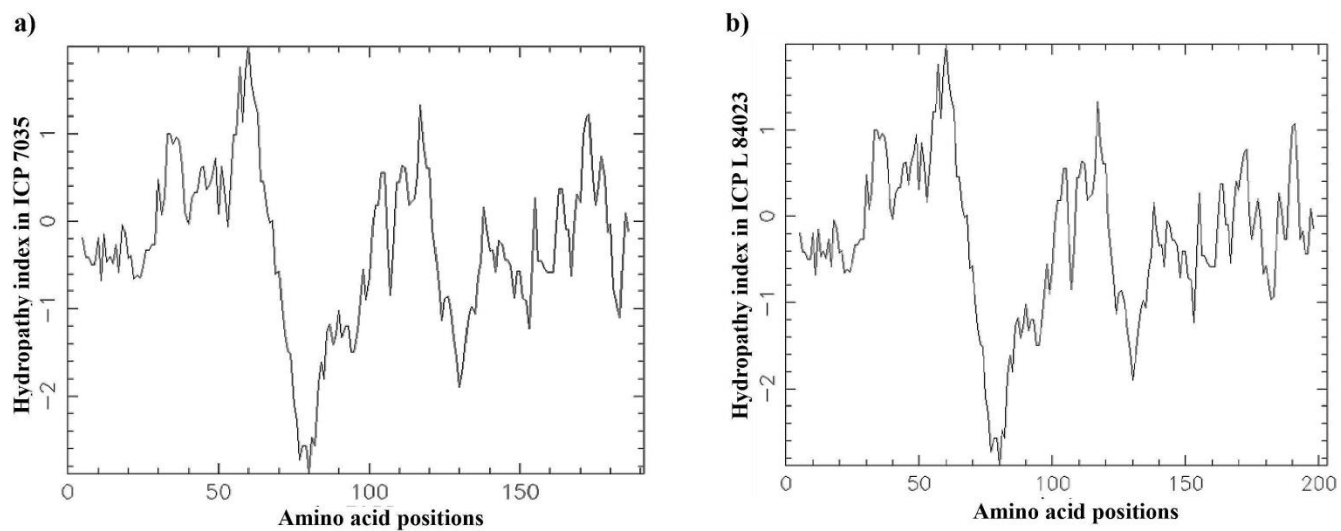

**Fig. S1:** Kyte and Doolittle hydropathy plot in a) ICP 7035 and b) ICPL 84023

Supplement: Supplemental Information 1 [file peerj-09-10888-s001.pdf]

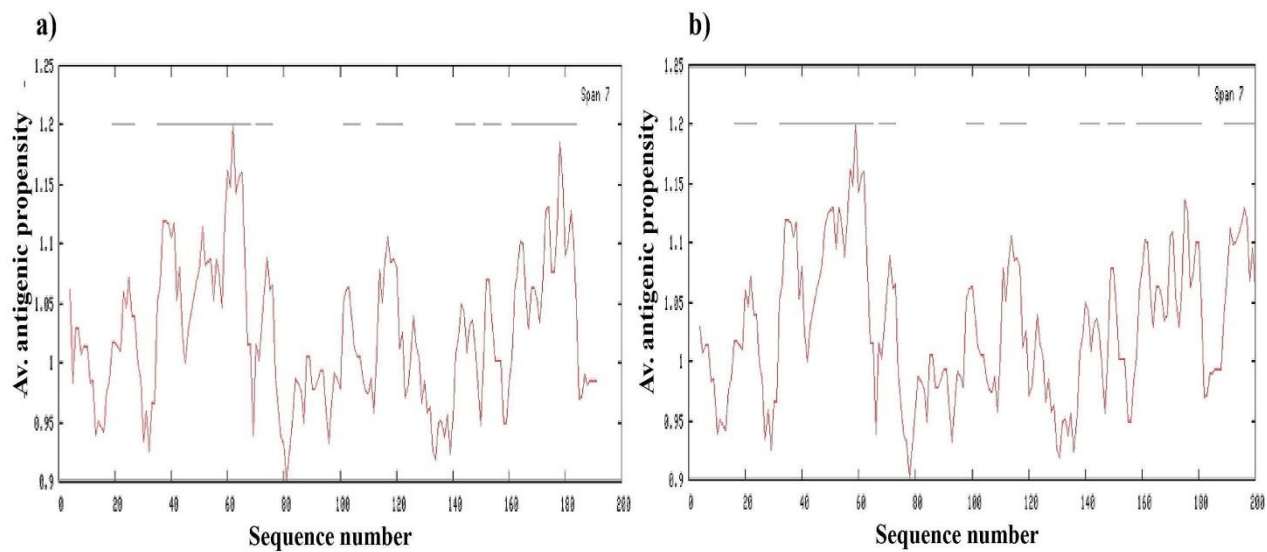

**Fig. S3:** Average antigenic propensity in a) ICP 7035 and b) ICPL 84023

Supplement: Supplemental Information 3 [file peerj-09-10888-s003.pdf]
