# Supplementary material for: In silico characterization and homology modeling of cytosolic APX gene predicts novel glycine residue modulating waterlogging stress response in pigeon pea
Source: PeerJ. 2021 May 12;9:e10888. doi: 10.7717/peerj.10888 (PMC8123230; doi:10.7717/peerj.10888)
Supplement: Supplemental Information 2 [file peerj-09-10888-s002.pdf]

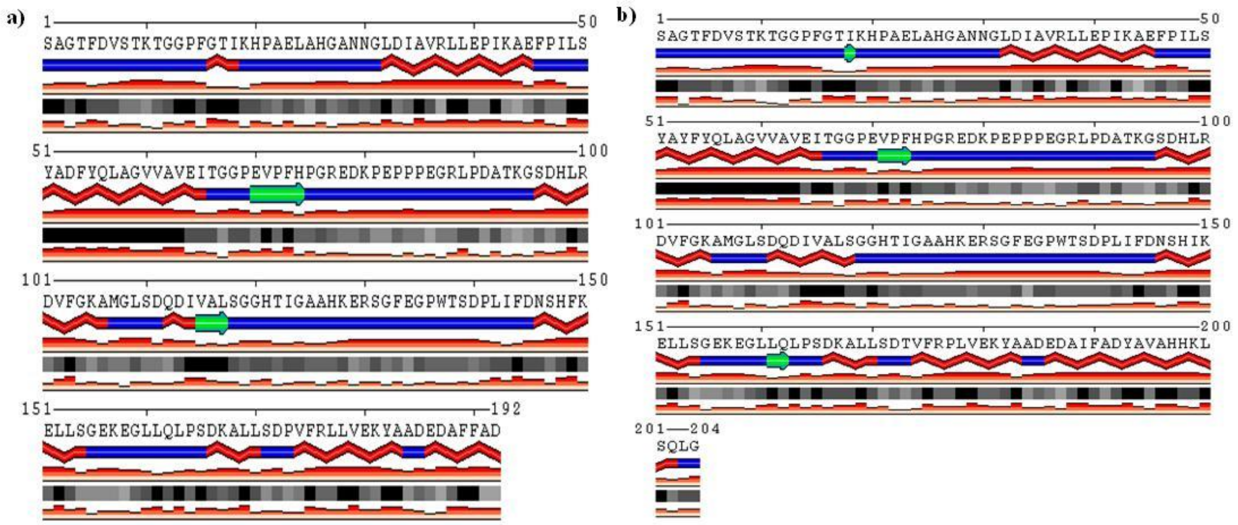

Fig. S2: Transmembrane domain structure prediction in a) ICP 7035 and b) ICPL 84023 where the alpha-helices are annotated with a red line, C coiled structure marked with a blue line, and the beta-sheet region is marked with green arrow.
