# Supplementary material for: In silico characterization and homology modeling of cytosolic APX gene predicts novel glycine residue modulating waterlogging stress response in pigeon pea
Source: PeerJ. 2021 May 12;9:e10888. doi: 10.7717/peerj.10888 (PMC8123230; doi:10.7717/peerj.10888)

Fig. S4: Ramachandran plots of a) all residue types, b) main chain bond length, c) main chain properties, d) side chain properties, e) residue properties, f) RMS distance from planarity and g) chi1-chi2 plots in ICP 7035 and ICPL 84023

a) Ramachandran plots of all residue types in ICP 7035 in pigeon pea

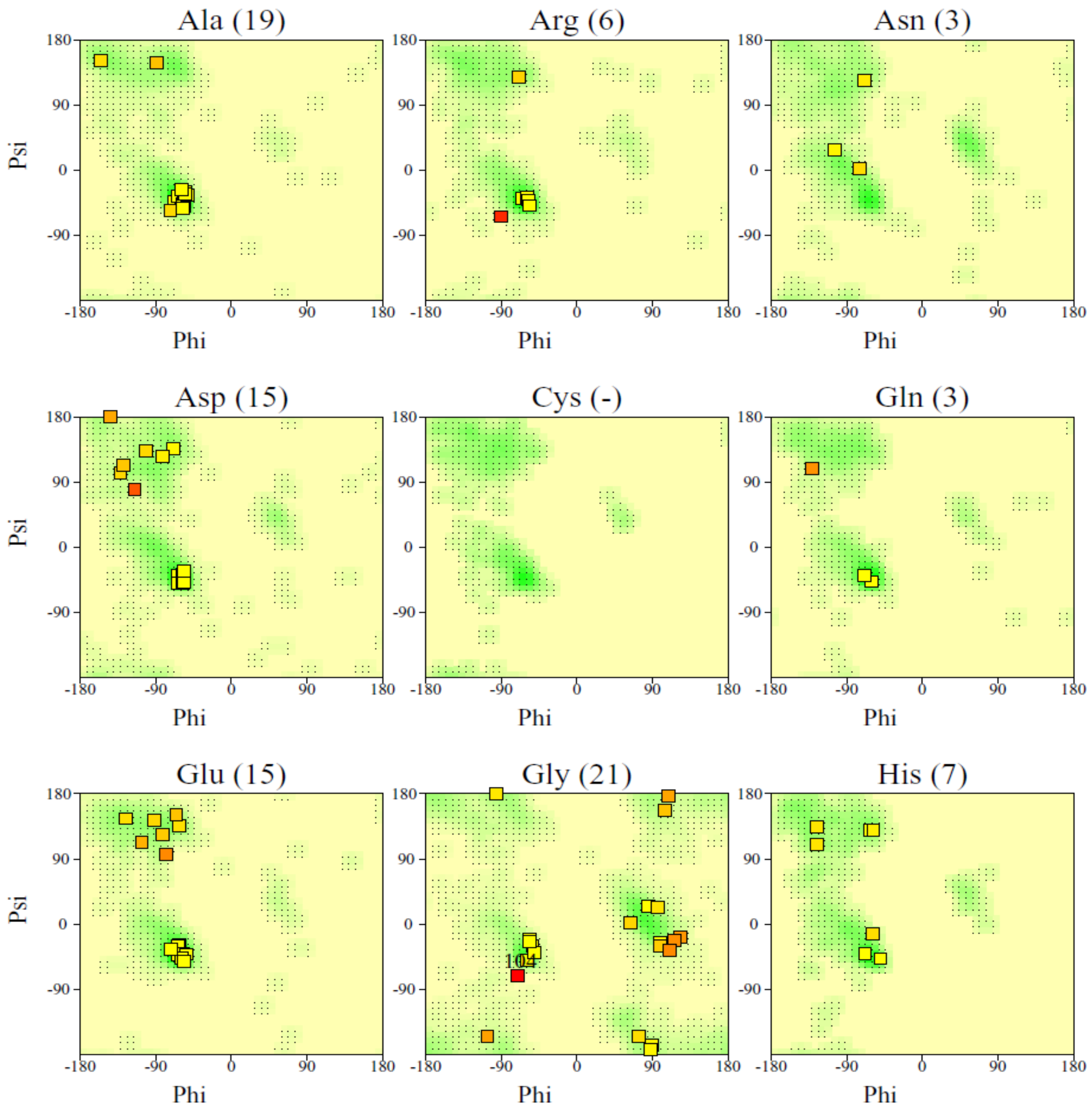

Numbers of residues are shown in brackets. Those in unfavourable conformations (score < -3.00) are labelled.
Shading shows favourable conformations as obtained from an analysis of 163 structures at resolution 2.0A or better.

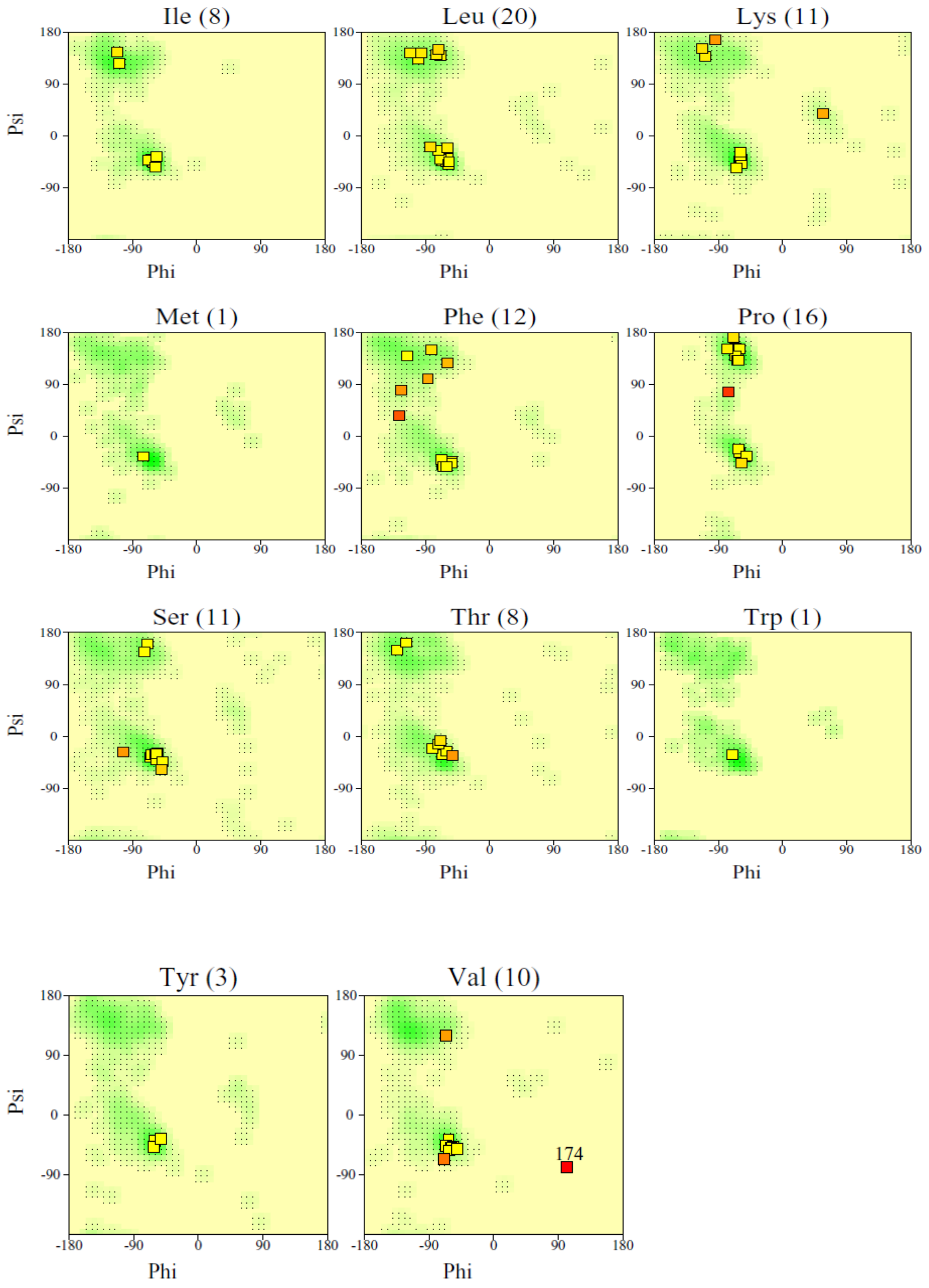
Ile (8)
Leu (20)
Lys (11)
Met (1)
Phe (12)
Pro (16)
Ser (11)
Thr (8)
Trp (1)
Tyr (3)
Val (10)
174
Psi
Phi
180
90
0
-90
-180
90
180

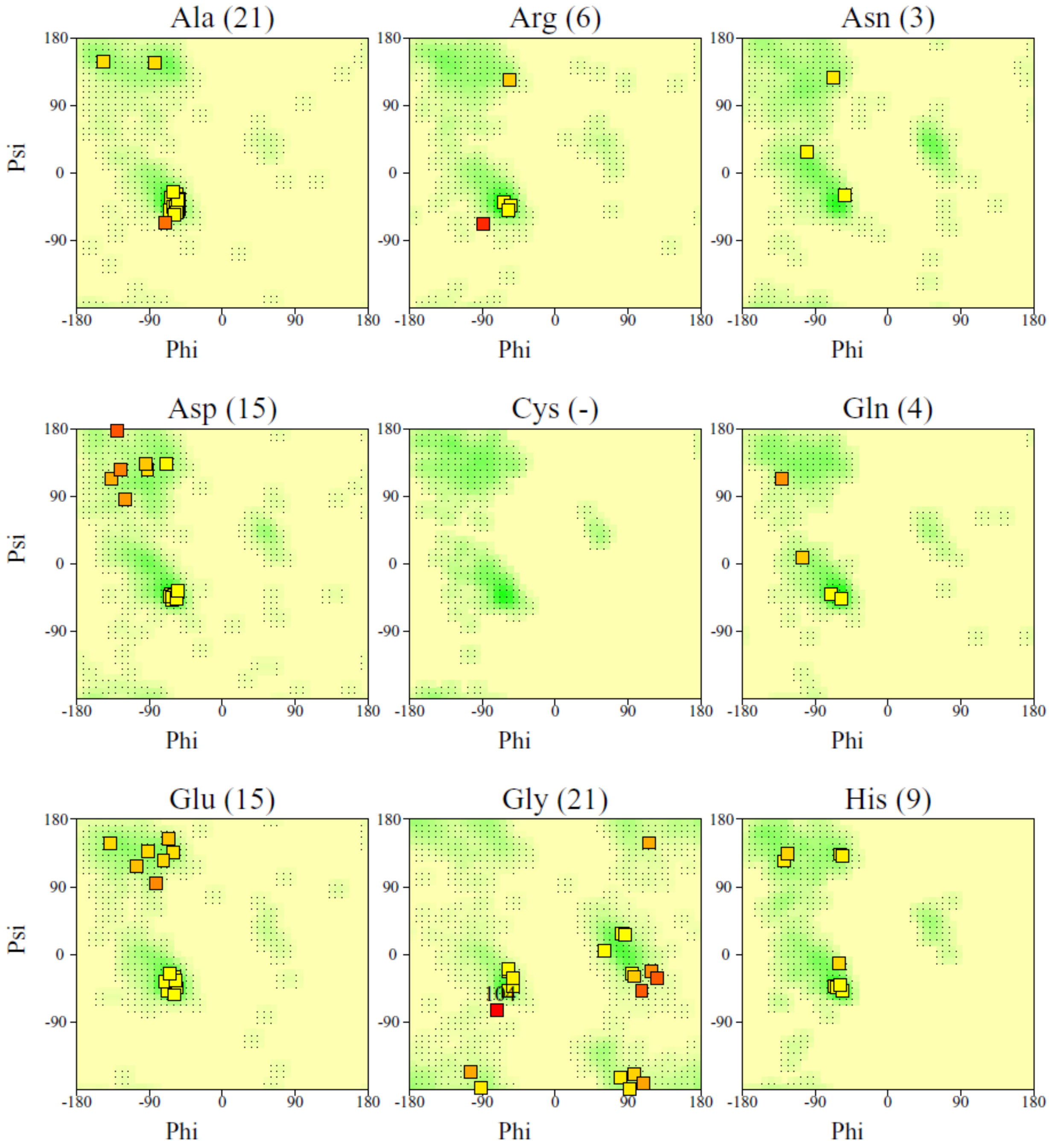

Numbers of residues are shown in brackets. Those in unfavourable conformations (score < -3.00) are labelled.
Shading shows favourable conformations as obtained from an analysis of 163 structures at resolution 2.0A or better.

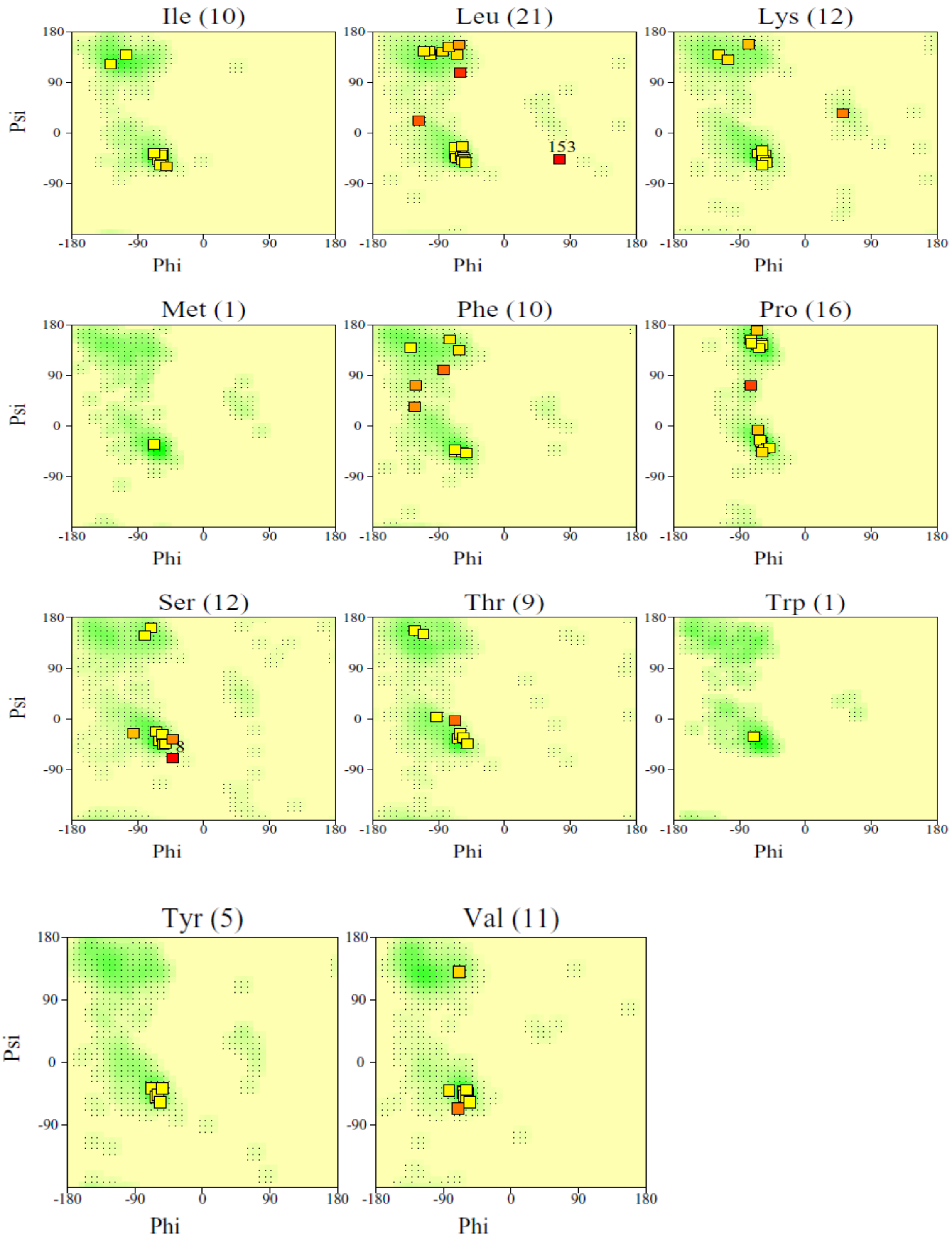

Ile (10)
Leu (21)
153
Lys (12)
Met (1)
Phe (10)
Pro (16)
Ser (12)
Thr (9)
Trp (1)
Tyr (5)
Val (11)
Psi
Phi
180
90
0
-90
-180

b) Main chain bond length

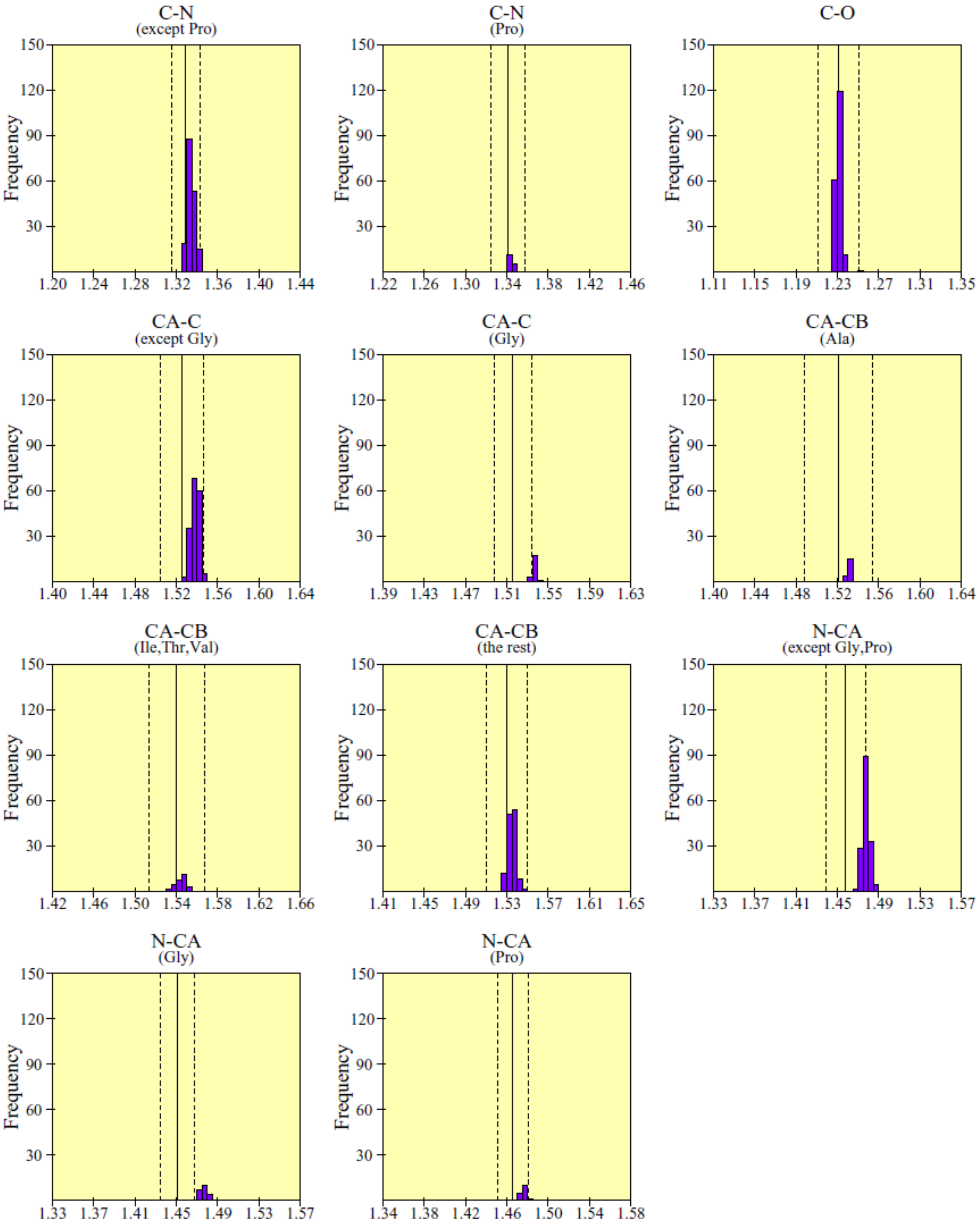

Black bars > 2.0 st. devs. from mean.

Solid and dashed lines represent the mean and standard deviation values as per Engh & Huber small-molecule data.

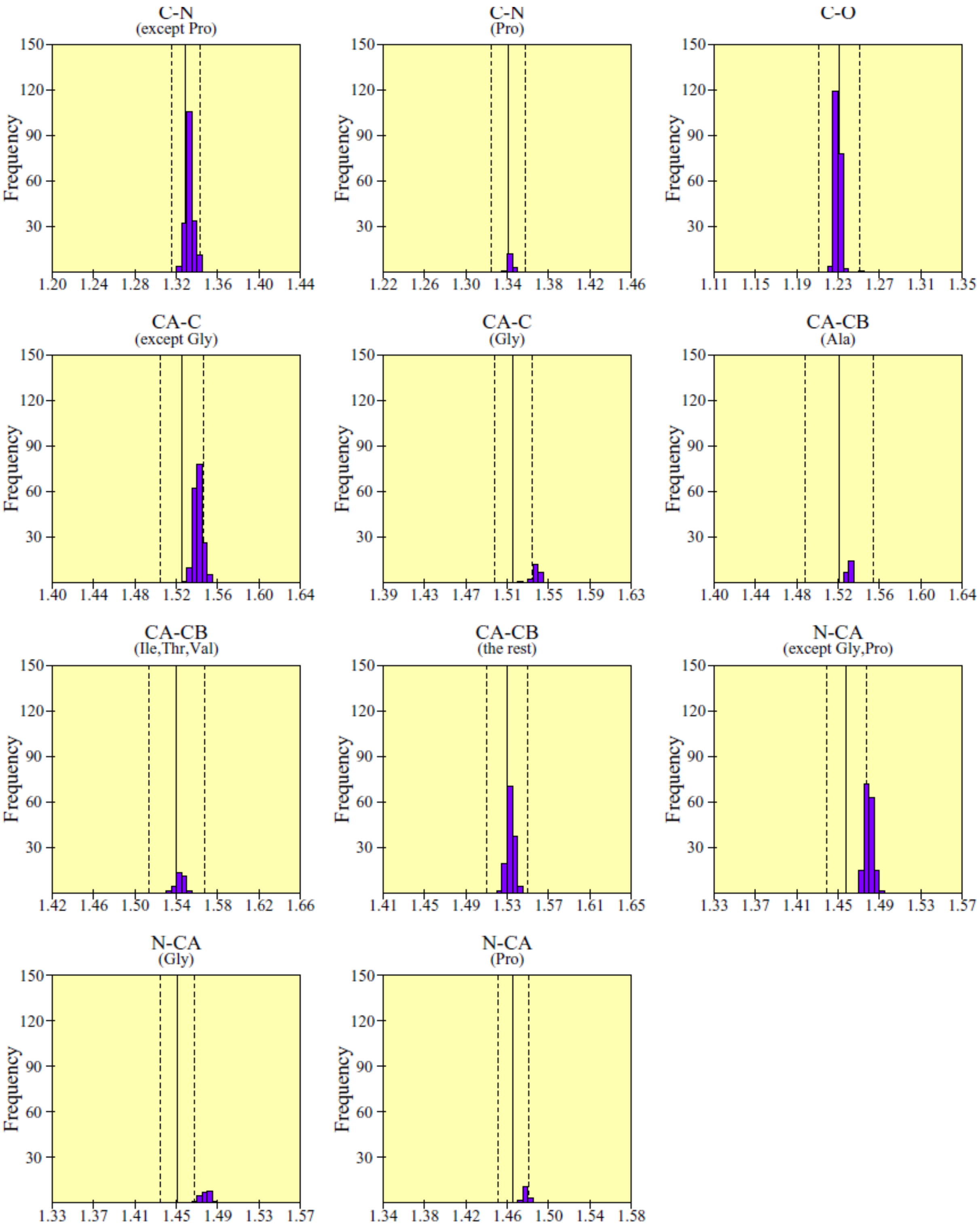

Black bars > 2.0 st. devs. from mean.

Solid and dashed lines represent the mean and standard deviation values as per Engh & Huber small-molecule data.

c) Main chain properties

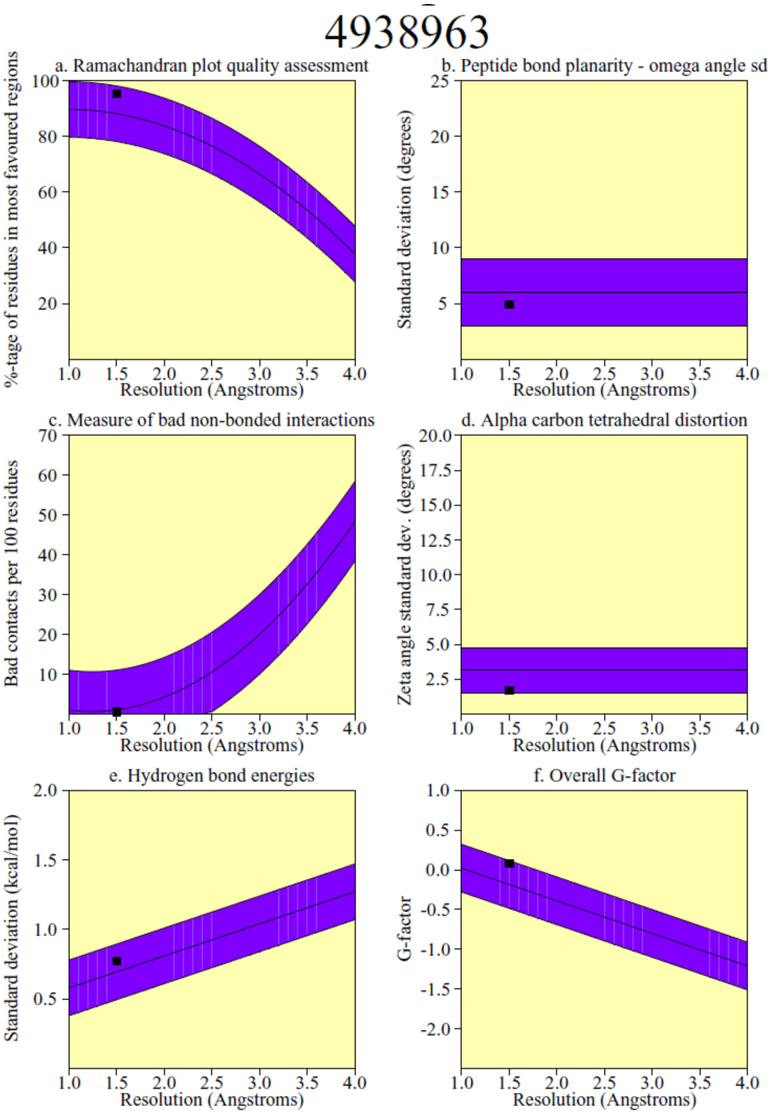

Plot statistics

Stereochemical parameter	No. of data pts	Parameter value	Comparison values Typical value	Band width	No. of band widths from mean	
a. %-tage residues in A, B, L	153	95.4	88.2	10.0	0.7	Inside
b. Omega angle st dev	191	4.9	6.0	3.0	-0.4	Inside
c. Bad contacts / 100 residues	1	0.5	1.0	10.0	-0.1	Inside
d. Zeta angle st dev	171	1.7	3.1	1.6	-0.9	Inside
e. H-bond energy st dev	112	0.8	0.7	0.2	0.4	Inside
f. Overall G-factor	192	0.1	-0.2	0.3	0.9	Inside

8150565

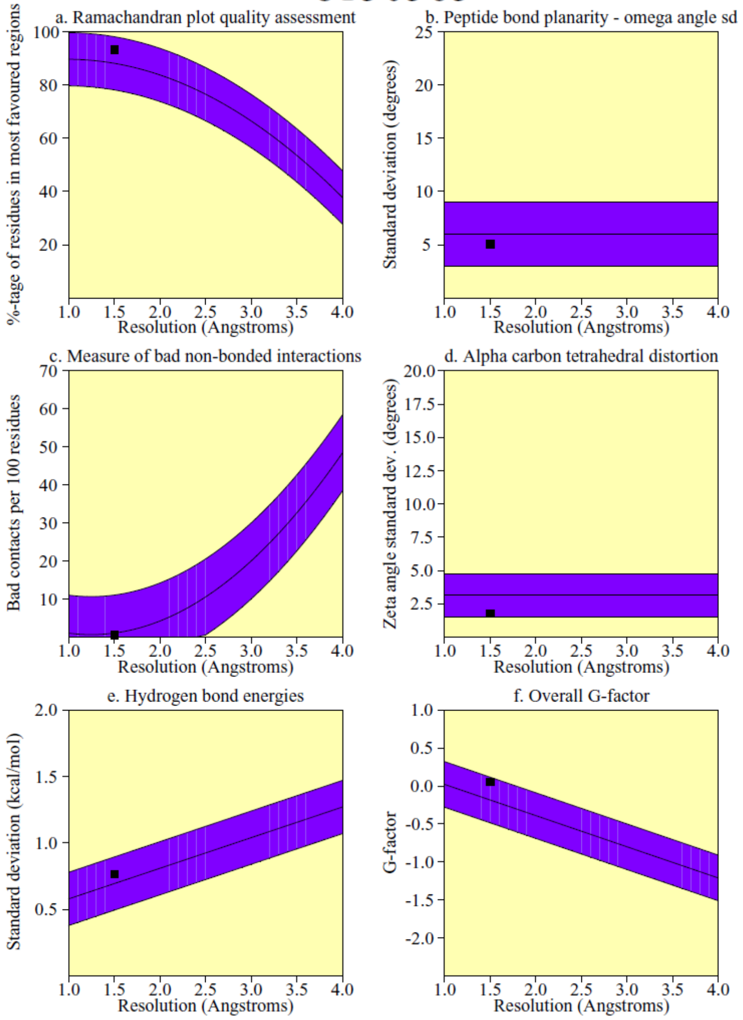

Plot statistics

Stereochemical parameter	No. of data pts	Parameter value	Comparison values Typical value	Band width	No. of band widths from mean	
a. %-tage residues in A, B, L	165	93.3	88.2	10.0	0.5	Inside
b. Omega angle st dev	203	5.1	6.0	3.0	-0.3	Inside
c. Bad contacts / 100 residues	1	0.5	1.0	10.0	-0.1	Inside
d. Zeta angle st dev	182	1.8	3.1	1.6	-0.8	Inside
e. H-bond energy st dev	120	0.8	0.7	0.2	0.3	Inside
f. Overall G-factor	204	0.1	-0.2	0.3	0.8	Inside

Main chain bond angle of ICP 7035

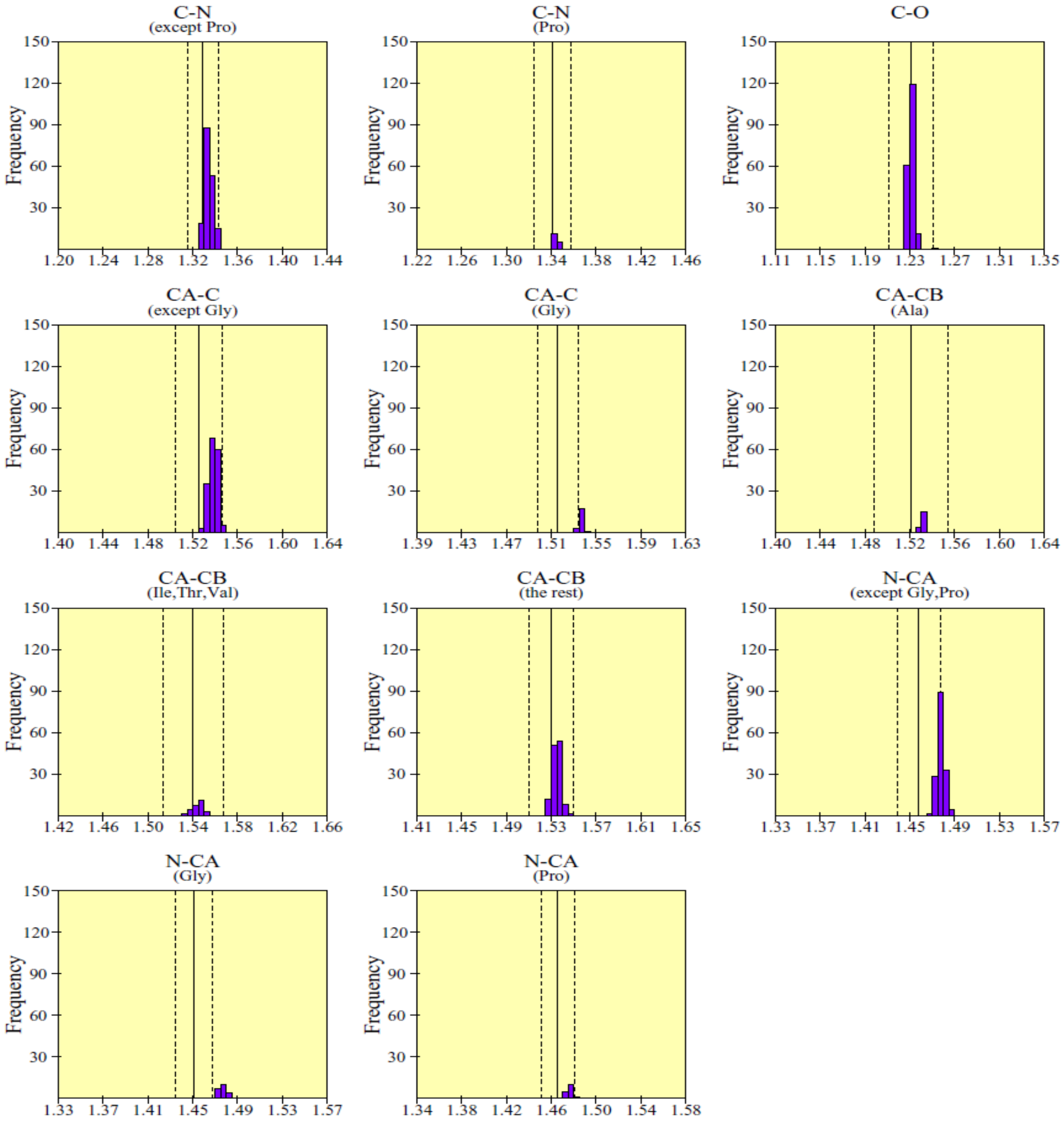

Black bars > 2.0 st. devs. from mean.

Solid and dashed lines represent the mean and standard deviation values as per Engh & Huber small-molecule data.

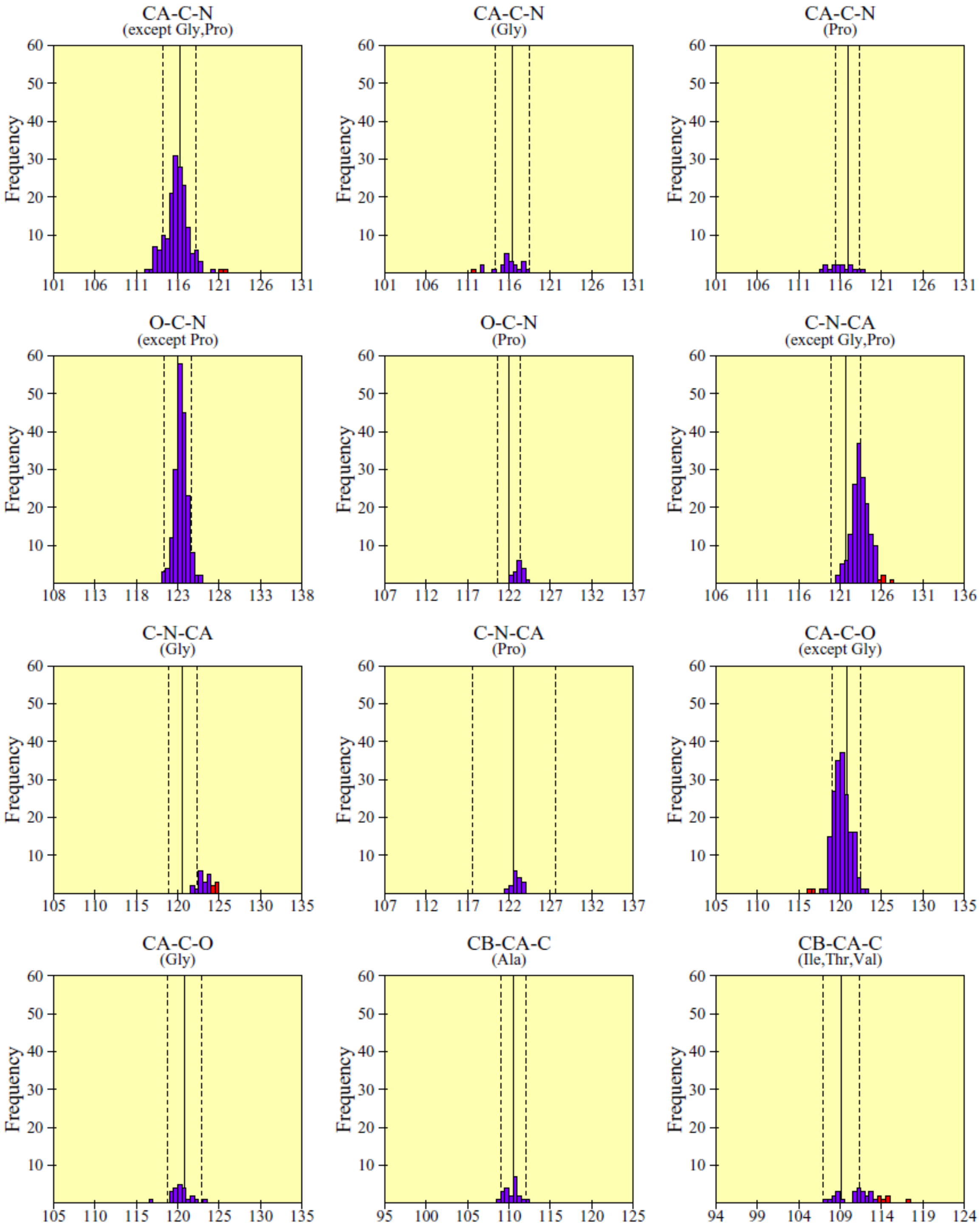

Black bars > 2.0 st. devs. from mean.
Solid and dashed lines represent the mean and standard deviation values as per Engh & Huber small-molecule data.

d) Side chain properties

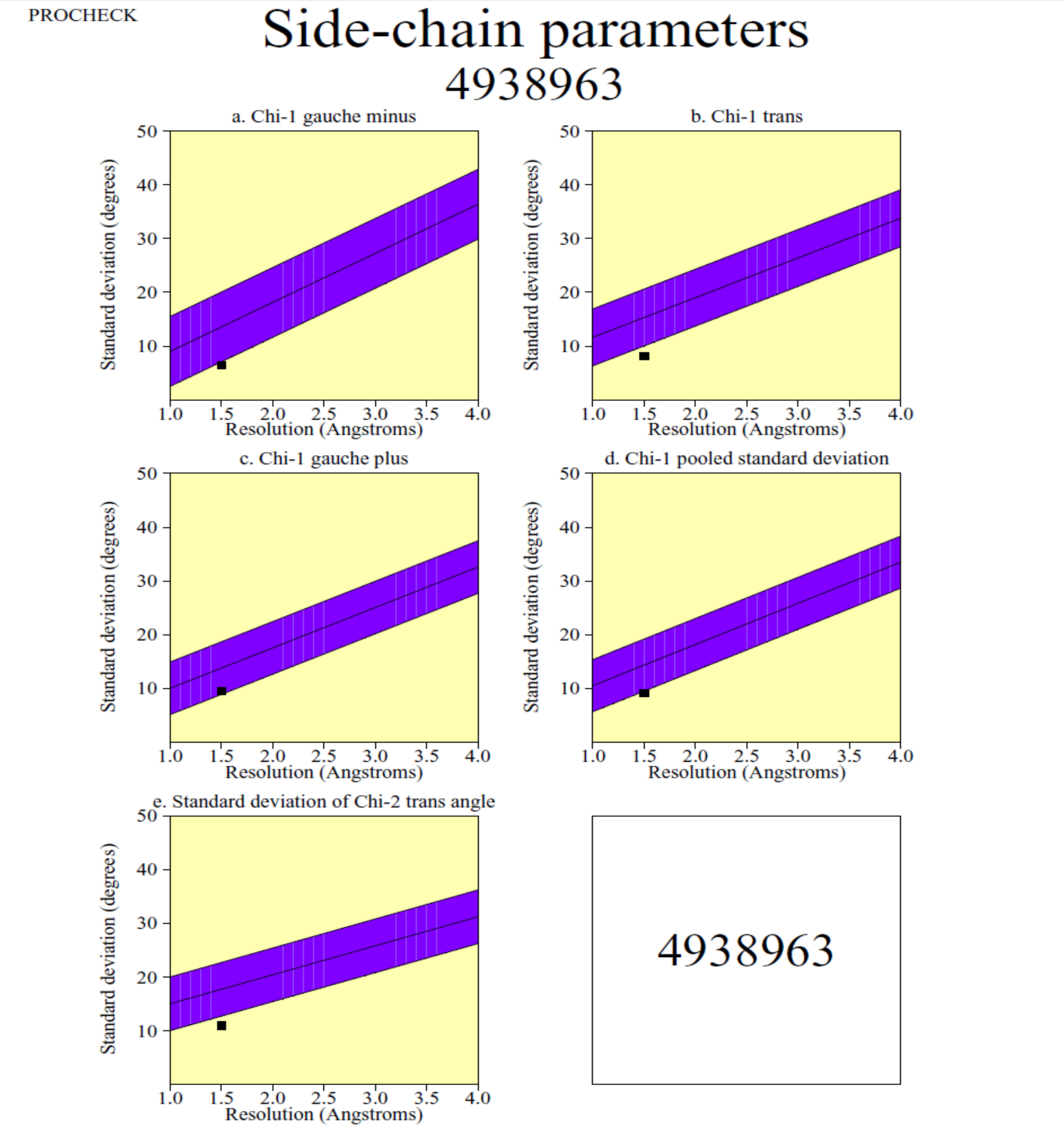

Plot statistics

Stereochemical parameter	No. of data pts	Parameter value	Comparison values Typical value	Band width	No. of band widths from mean	
a. Chi-1 gauche minus st dev	20	6.5	13.6	6.5	-1.1	BETTER
b. Chi-1 trans st dev	44	8.2	15.3	5.3	-1.3	BETTER
c. Chi-1 gauche plus st dev	72	9.5	13.8	4.9	-0.9	Inside
d. Chi-1 pooled st dev	136	9.2	14.3	4.8	-1.1	BETTER
e. Chi-2 trans st dev	42	11.0	17.7	5.0	-1.3	BETTER

PROCHECK

Side-chain parameters

8150565

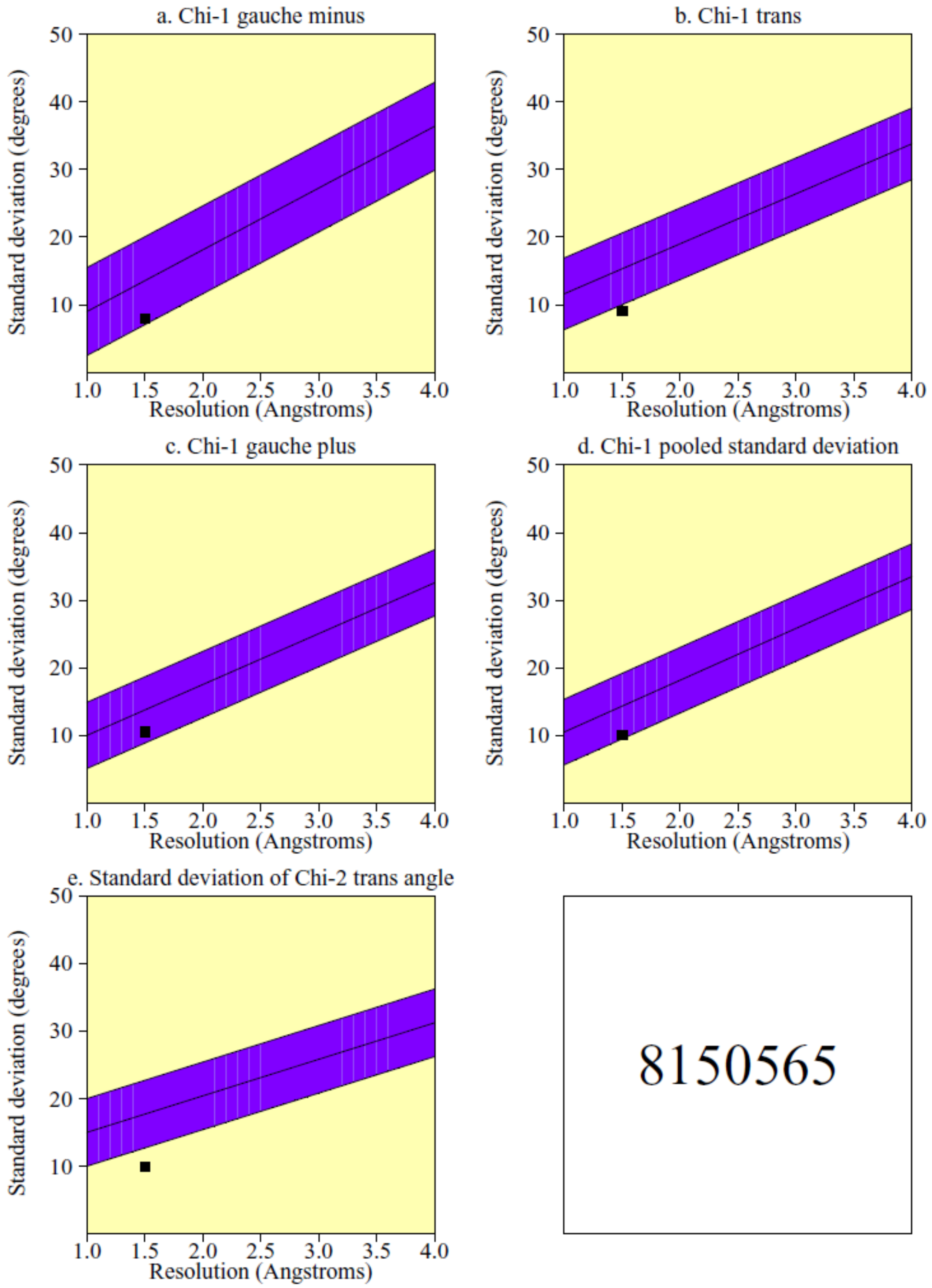

Plot statistics

Stereochemical parameter	No. of data pts	Parameter value	Comparison values Typical value	Band width	No. of band widths from mean	
a. Chi-1 gauche minus st dev	19	8.0	13.6	6.5	-0.9	Inside
b. Chi-1 trans st dev	49	9.1	15.3	5.3	-1.2	BETTER
c. Chi-1 gauche plus st dev	77	10.5	13.8	4.9	-0.7	Inside
d. Chi-1 pooled st dev	145	10.1	14.3	4.8	-0.9	Inside
e. Chi-2 trans st dev	48	9.9	17.7	5.0	-1.6	BETTER

e) Residue properties

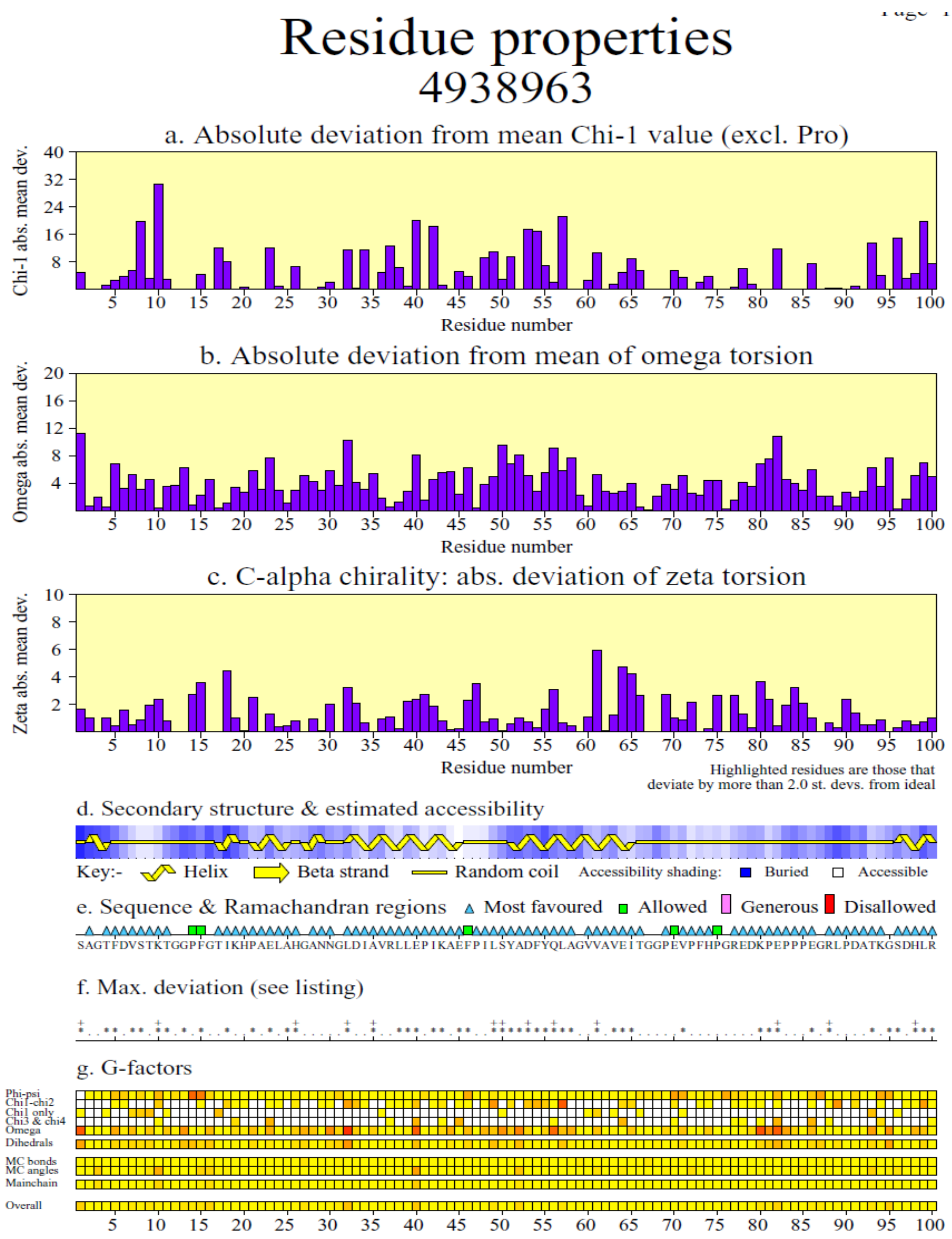

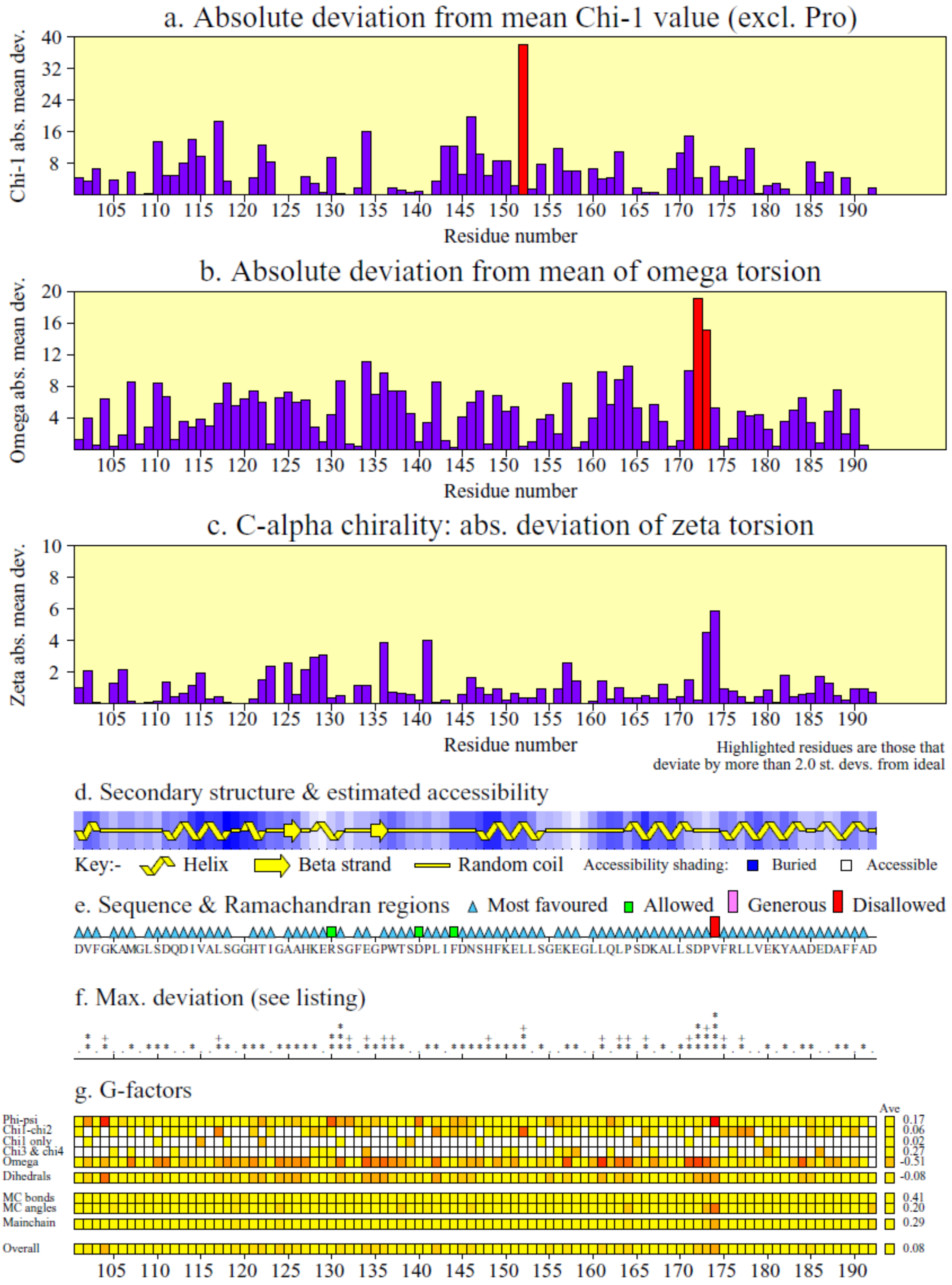
a. Absolute deviation from mean Chi-1 value (excl. Pro)
Chi-1 abs. mean dev.
Residue number
b. Absolute deviation from mean of omega torsion
Omega abs. mean dev.
Residue number
c. C-alpha chirality: abs. deviation of zeta torsion
Zeta abs. mean dev.
Residue number
Highlighted residues are those that deviate by more than 2.0 st. devs. from ideal
d. Secondary structure & estimated accessibility
Key:- Helix Beta strand Random coil Accessibility shading: Buried Accessible
e. Sequence & Ramachandran regions Most favoured Allowed Generous Disallowed
DVFGKAMGLSDQDIVALSGGHTIGAAHKERSGFEGPWTSDPLIFDNSHFKELLSGEKEGLLQLPSDKALLSDPVFRLLVEKYAADEDAFFAD
f. Max. deviation (see listing)
g. G-factors
Ave
Phi-psi 0.17
Chi1-chi2 0.06
Chi1 only 0.02
Chi3 & chi4 0.27
Omega -0.51
Dihedrals -0.08
MC bonds 0.41
MC angles 0.20
Mainchain 0.29
Overall 0.08
105 110 115 120 125 130 135 140 145 150 155 160 165 170 175 180 185 190

Residue properties
8150565

a. Absolute deviation from mean Chi-1 value (excl. Pro)

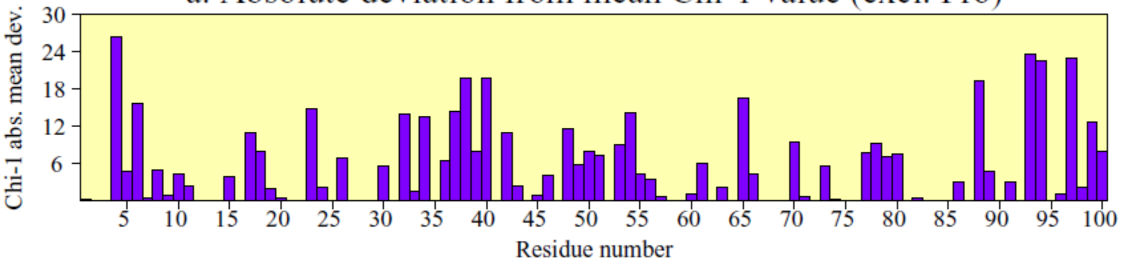

b. Absolute deviation from mean of omega torsion

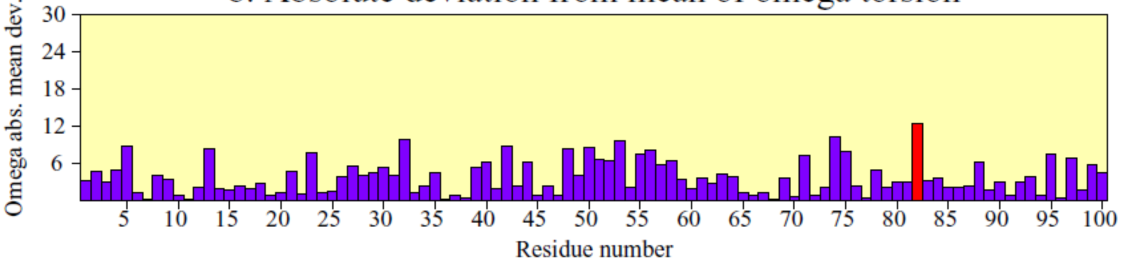

c. C-alpha chirality: abs. deviation of zeta torsion

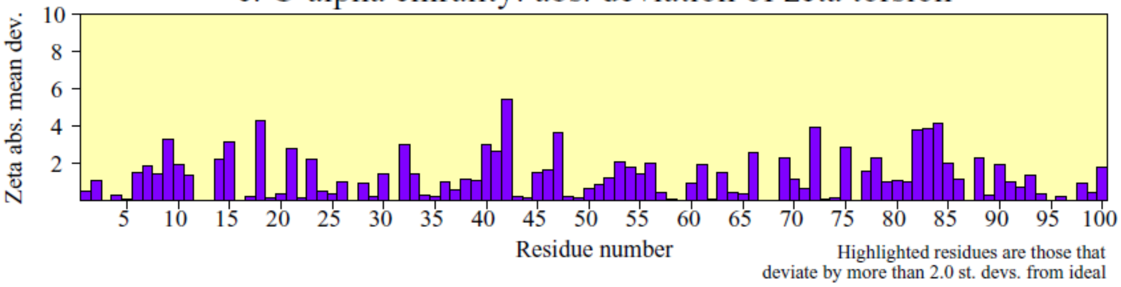

Highlighted residues are those that deviate by more than 2.0 st. devs. from ideal

d. Secondary structure & estimated accessibility

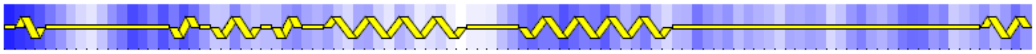

Key:- Helix Beta strand Random coil Accessibility shading: Buried Accessible

e. Sequence & Ramachandran regions Most favoured Allowed Generous Disallowed

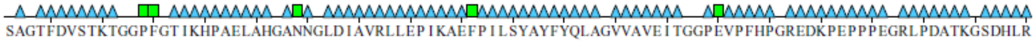

f. Max. deviation (see listing)

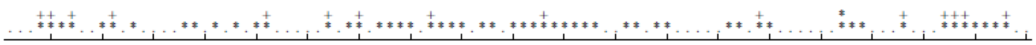

g. G-factors

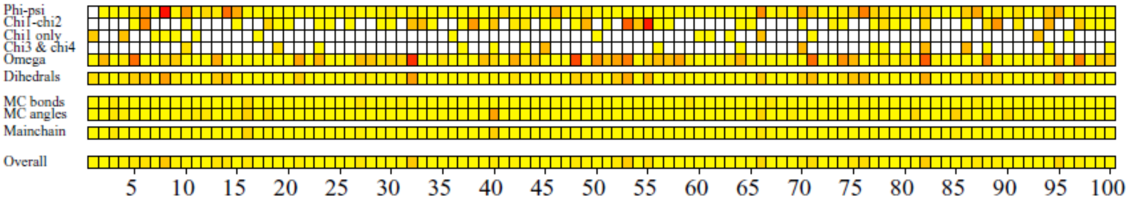

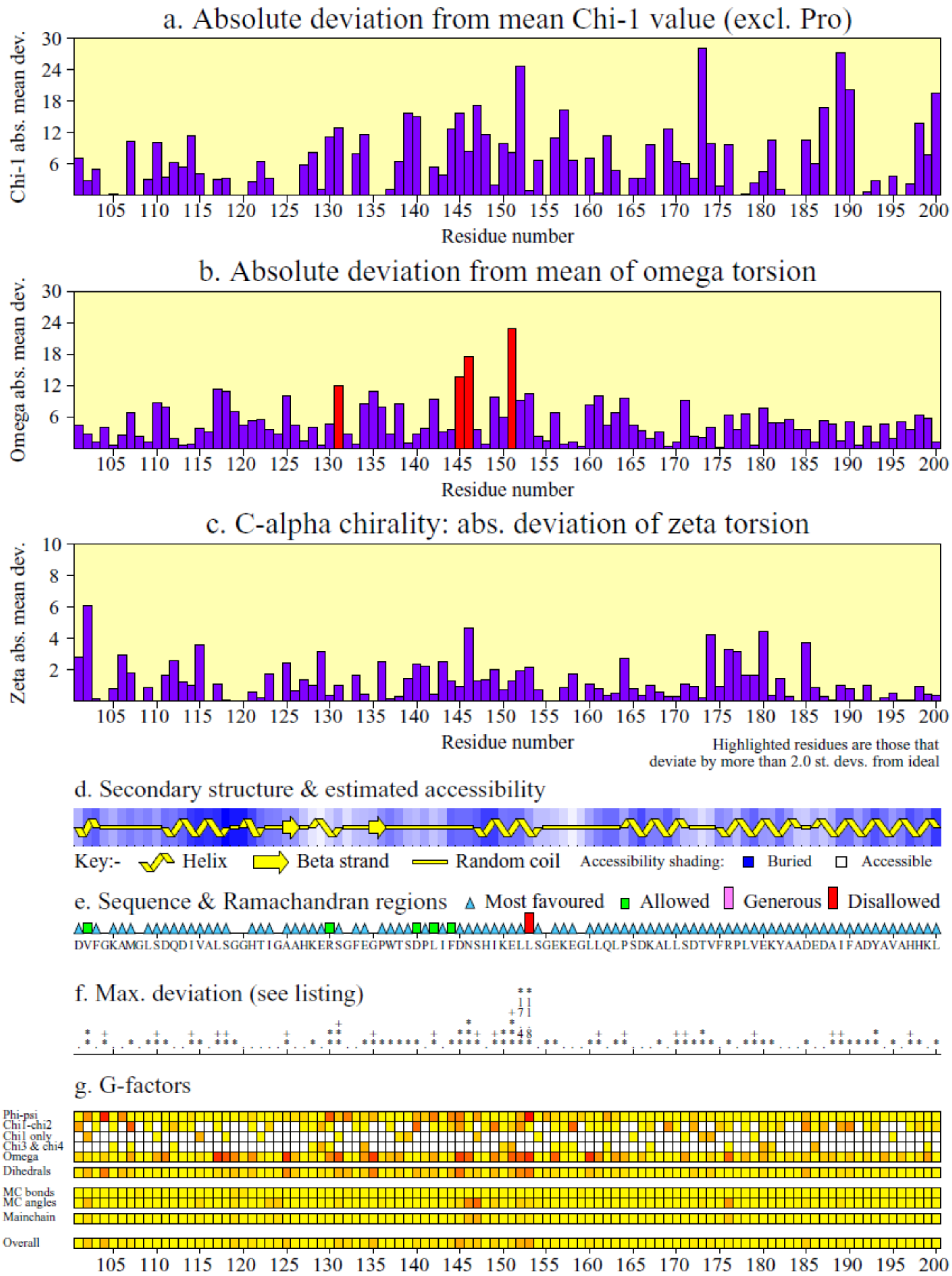
a. Absolute deviation from mean Chi-1 value (excl. Pro)
Chi-1 abs. mean dev.
Residue number
b. Absolute deviation from mean of omega torsion
Omega abs. mean dev.
Residue number
c. C-alpha chirality: abs. deviation of zeta torsion
Zeta abs. mean dev.
Residue number
Highlighted residues are those that deviate by more than 2.0 st. devs. from ideal
d. Secondary structure & estimated accessibility
Key:- Helix Beta strand Random coil Accessibility shading: Buried Accessible
e. Sequence & Ramachandran regions Most favoured Allowed Generous Disallowed
DVFGKAMGLSDQDIVALSGGHTIGAAHKERSGFEGPWTSDPLIFDNSHIKELLSGEKEGLLQLPSDKALLSDTVFRPLVEKYAADEDAIFADYAVAHHKL
f. Max. deviation (see listing)
g. G-factors
Phi-psi
Chi1-chi2
Chi1 only
Chi3 & chi4
Omega
Dihedrals
MC bonds
MC angles
Mainchain
Overall

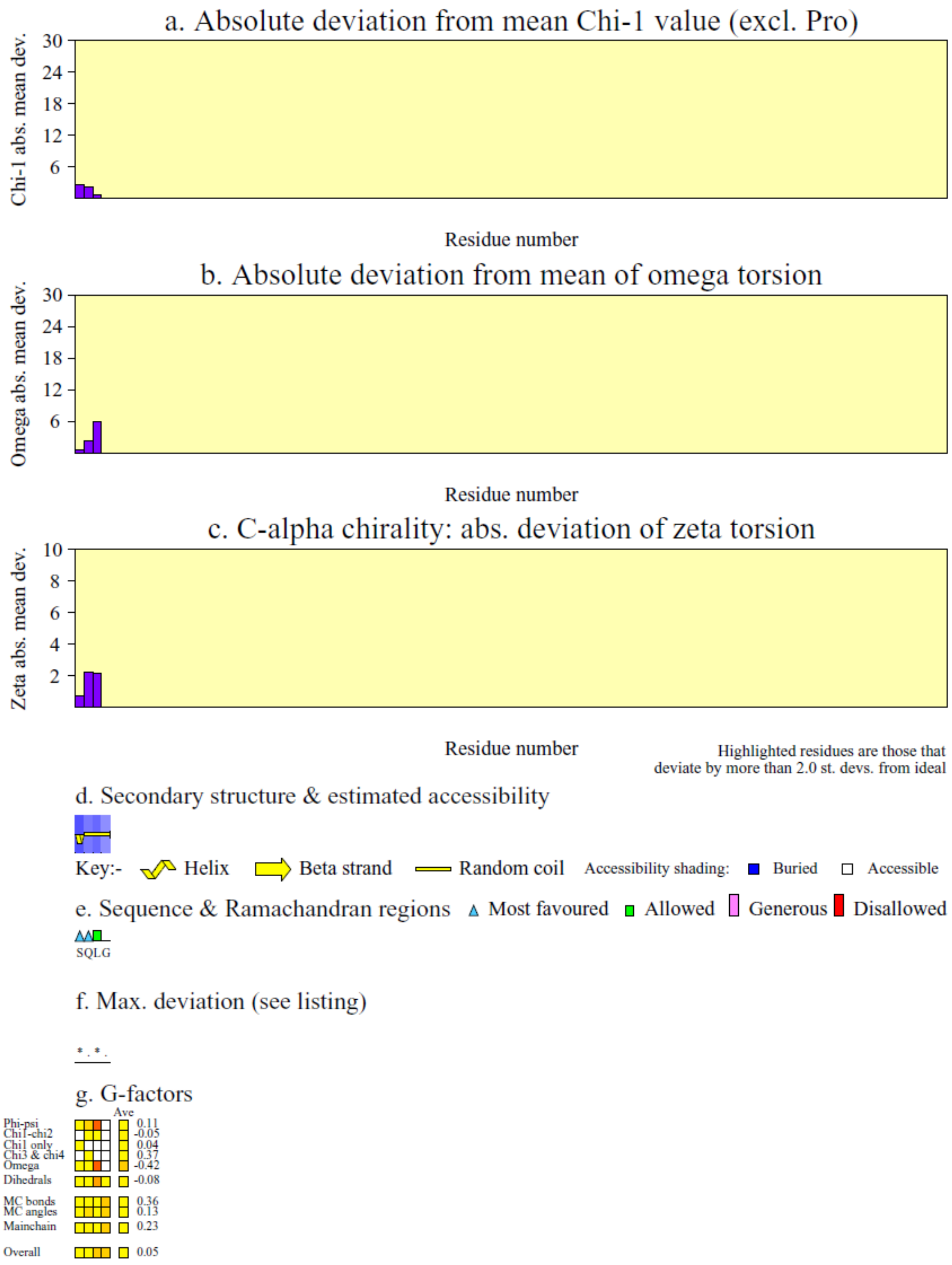
a. Absolute deviation from mean Chi-1 value (excl. Pro)
Chi-1 abs. mean dev.
30
24
18
12
6
Residue number
b. Absolute deviation from mean of omega torsion
Omega abs. mean dev.
30
24
18
12
6
Residue number
c. C-alpha chirality: abs. deviation of zeta torsion
Zeta abs. mean dev.
10
8
6
4
2
Residue number
Highlighted residues are those that deviate by more than 2.0 st. devs. from ideal
d. Secondary structure & estimated accessibility
Key:- Helix
Beta strand
Random coil
Accessibility shading:
Buried
Accessible
e. Sequence & Ramachandran regions
Most favoured
Allowed
Generous
Disallowed
SQLG
f. Max. deviation (see listing)
* . * .
g. G-factors
Ave
Phi-psi 0.11
Chi1-chi2 -0.05
Chi1 only 0.04
Chi3 & chi4 0.37
Omega -0.42
Dihedrals -0.08
MC bonds 0.36
MC angles 0.13
Mainchain 0.23
Overall 0.05

f) RMS distance from planarity

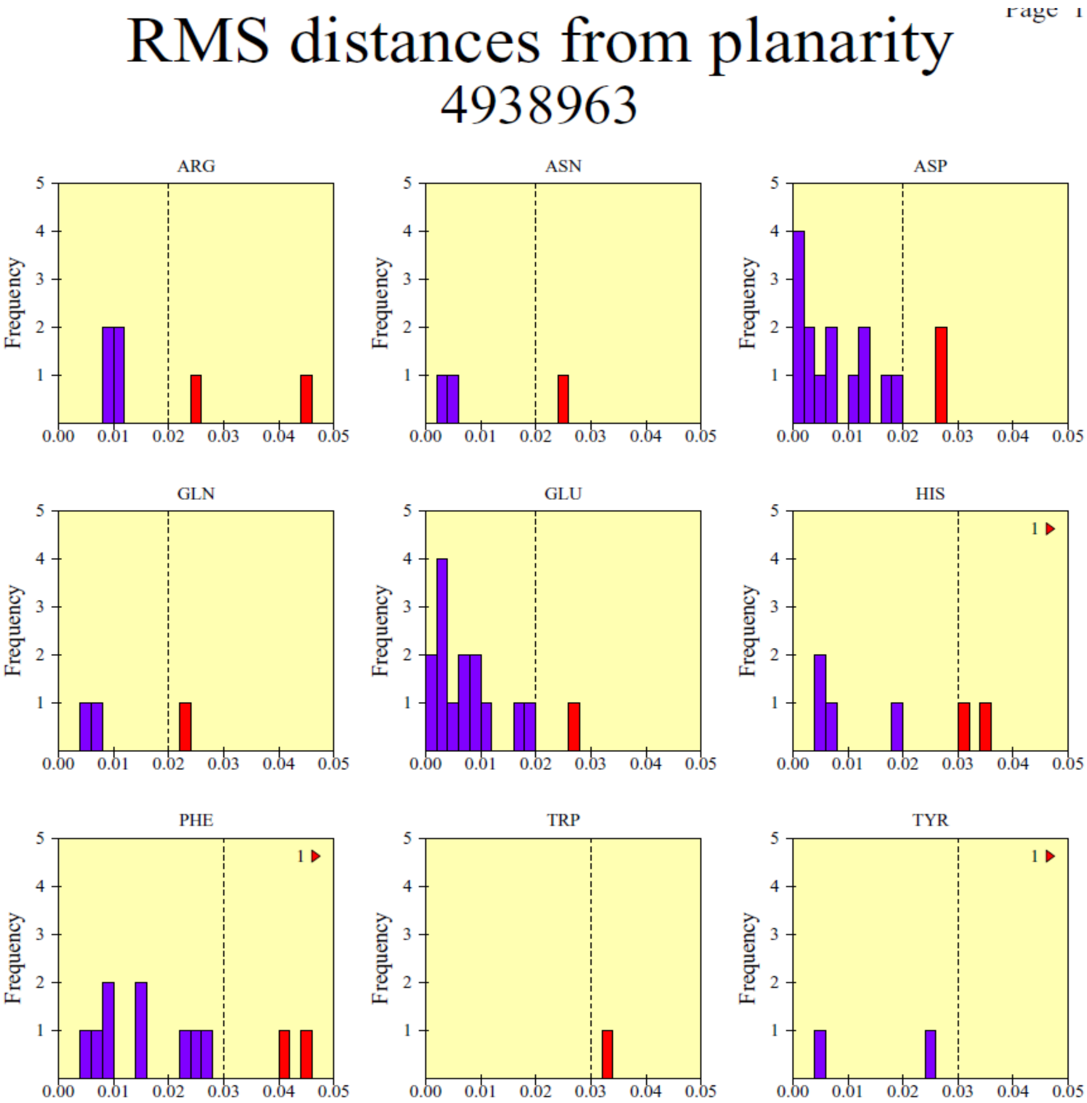

Histograms showing RMS distances of planar atoms from best-fit plane.
Black bars indicate large deviations from planarity: RMS dist > 0.03 for rings, and > 0.02 otherwise.

▶ signifies data points off the graph in the direction shown.

RMS distances from planarity
8150565

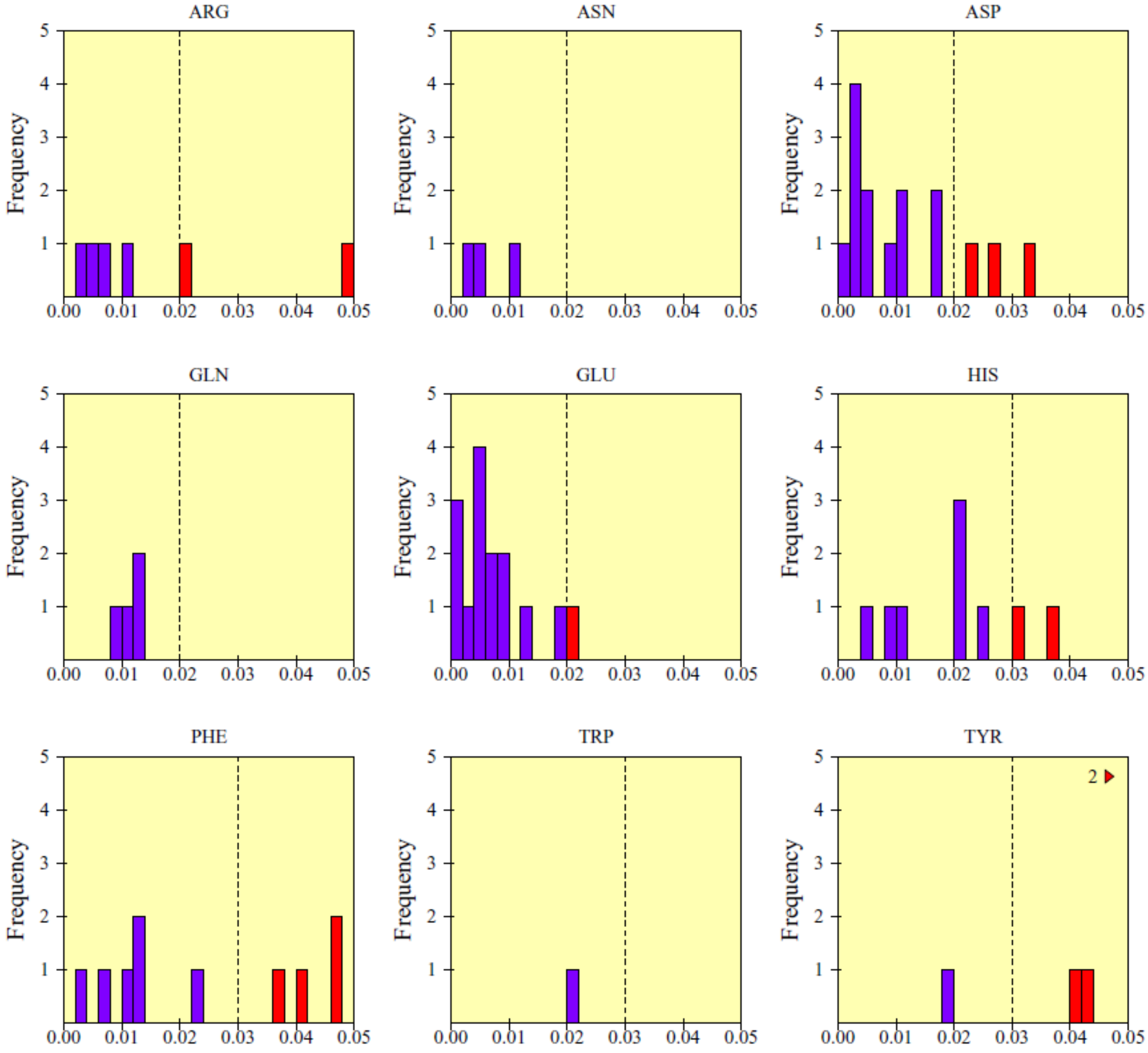

Histograms showing RMS distances of planar atoms from best-fit plane.
Black bars indicate large deviations from planarity: RMS dist > 0.03 for rings, and > 0.02 otherwise.

▶ signifies data points off the graph in the direction shown.

g) All Chi1-Chi2 plots of ICP 7035 and ICPL 84023 in pigeon pea

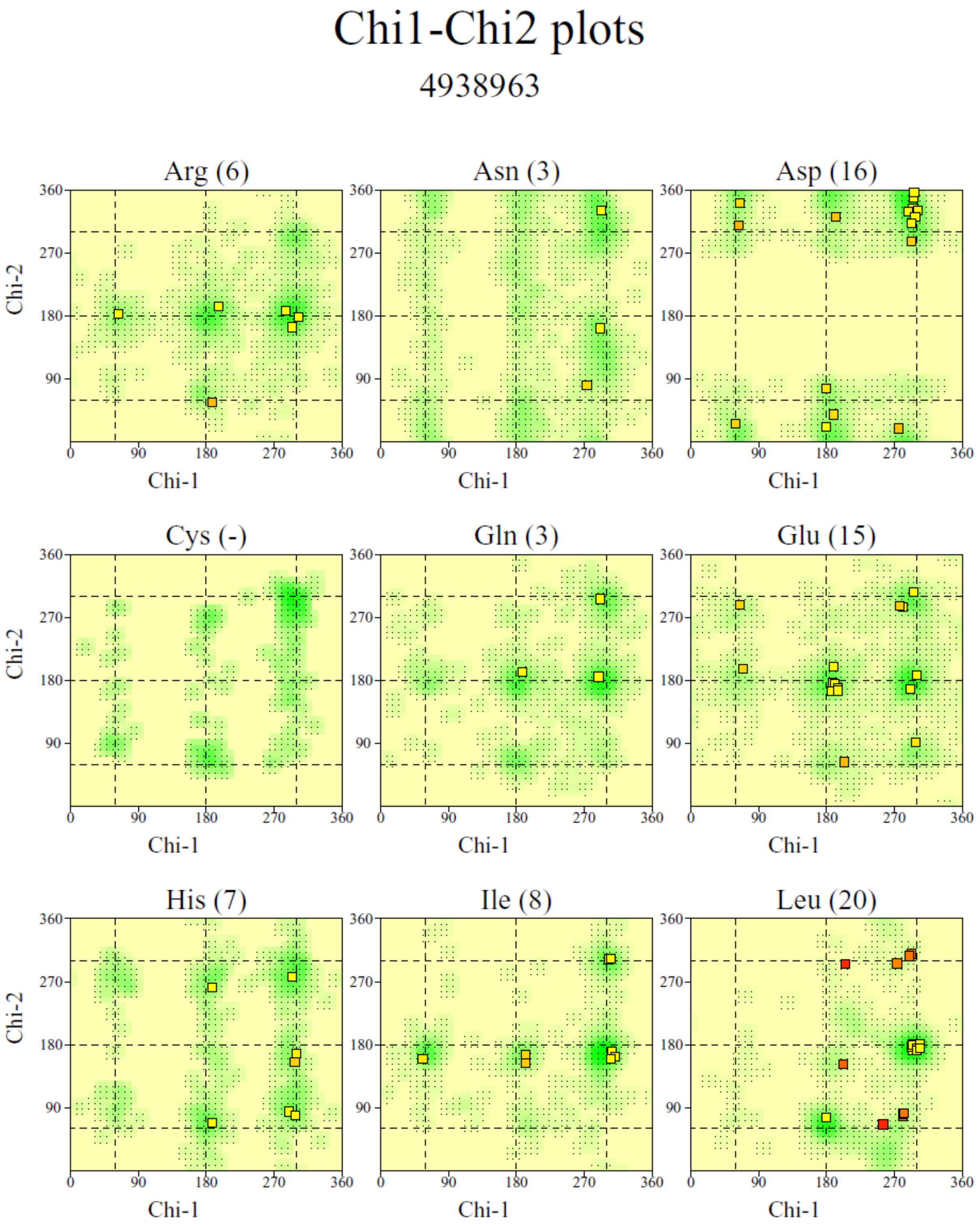

Numbers of residues are shown in brackets. Those in unfavourable conformations (score < -3.00) are labelled.
Shading shows favourable conformations as obtained from an analysis of 163 structures at resolution 2.0A or better.

Chi1-Chi2 plots

4938963

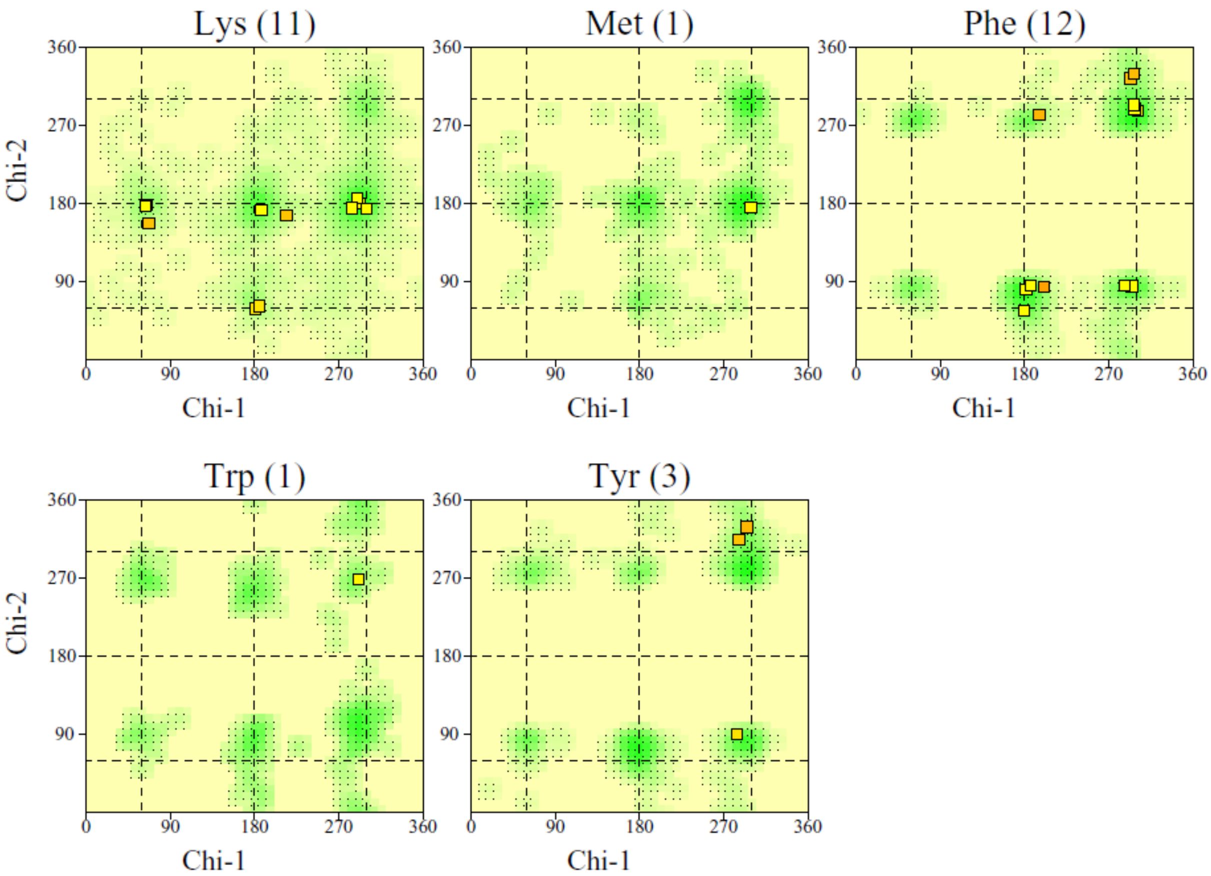

Chi1-Chi2 plots

8150565

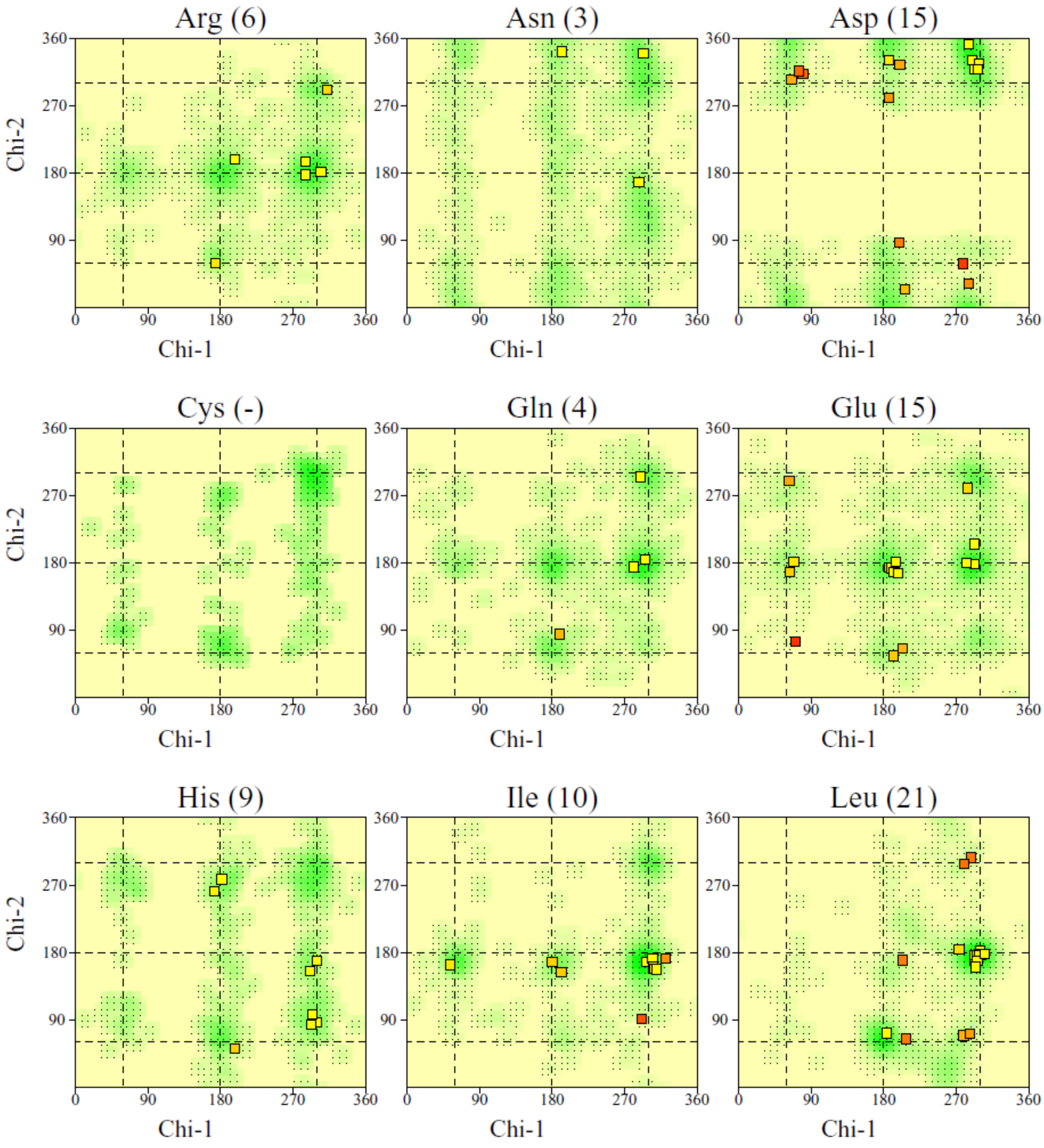

Numbers of residues are shown in brackets. Those in unfavourable conformations (score < -3.00) are labelled.
Shading shows favourable conformations as obtained from an analysis of 163 structures at resolution 2.0A or better.

Chi1-Chi2 plots

8150565

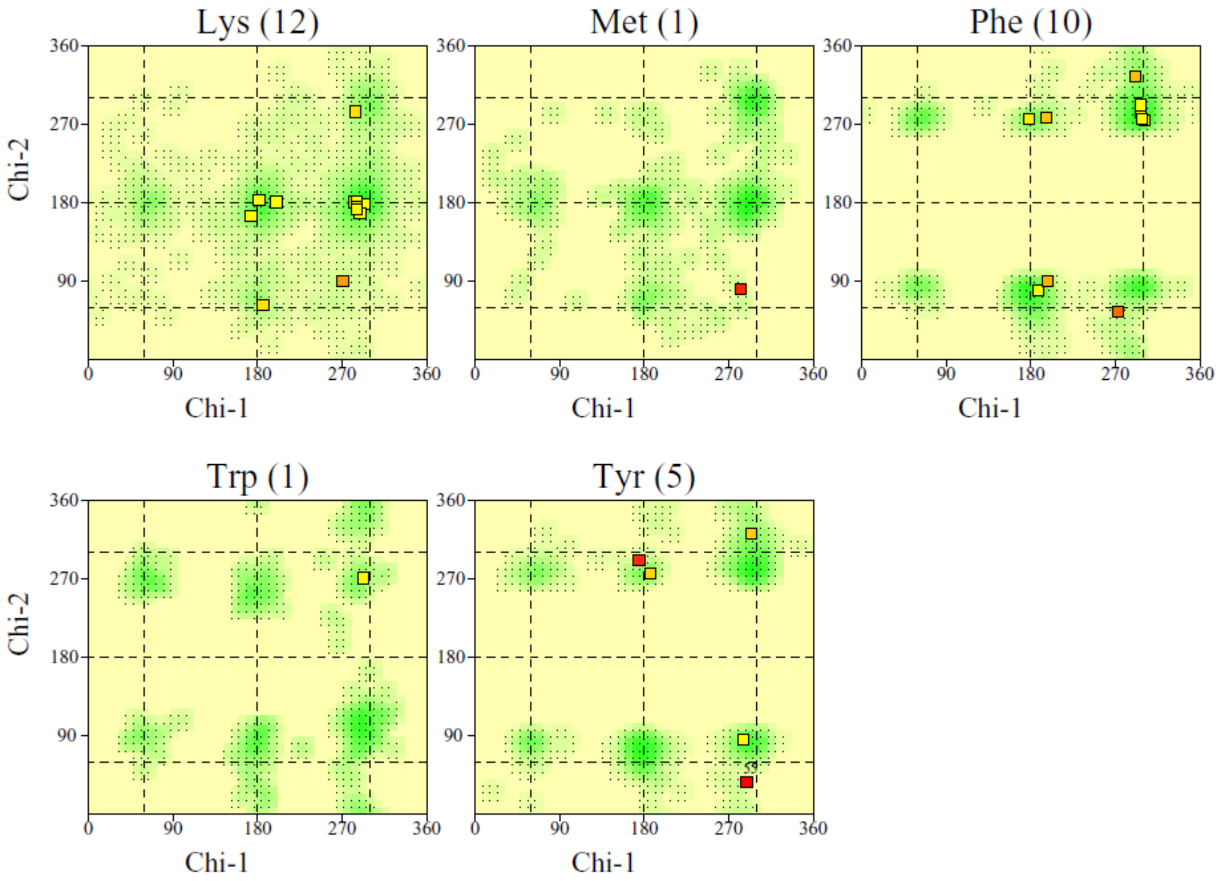

Supplement: Supplemental Information 4 [file peerj-09-10888-s004.pdf]
