# Supplementary material for: In silico characterization and homology modeling of cytosolic APX gene predicts novel glycine residue modulating waterlogging stress response in pigeon pea
Source: PeerJ. 2021 May 12;9:e10888. doi: 10.7717/peerj.10888 (PMC8123230; doi:10.7717/peerj.10888)
Supplement: Supplemental Information 5 [file peerj-09-10888-s005.pdf]

Fig. S5: Verify 3D image of a) ICP 7035 (84.38% of the residues have averaged 3D-1D score >= 0.2) and b) ICPL 84023 (87.25% of the residues have averaged 3D-1D score >= 0.2)

a)

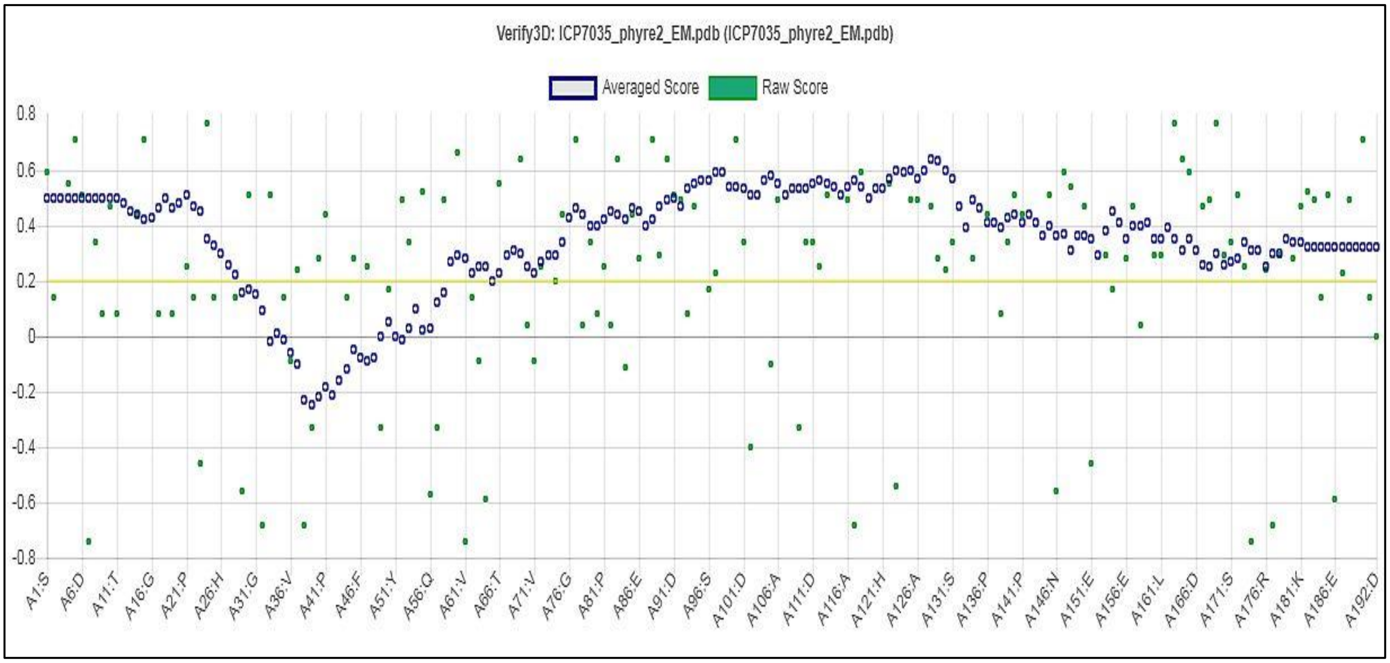

b)

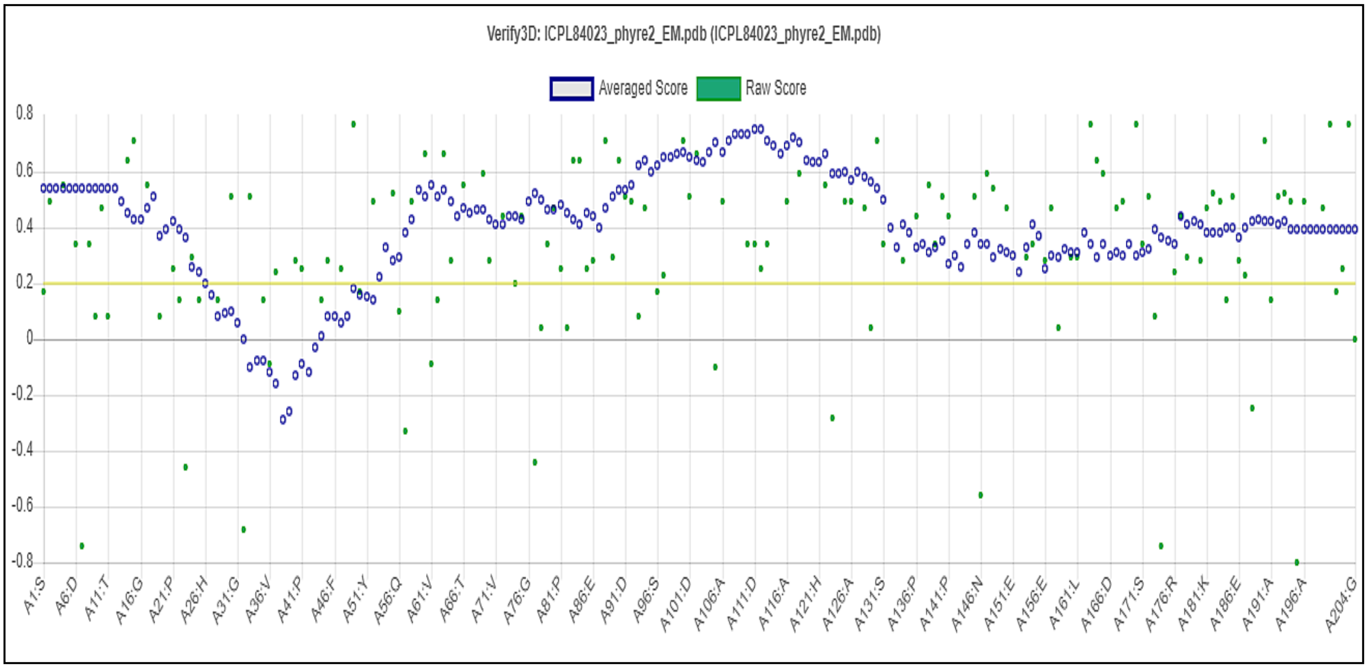

a)

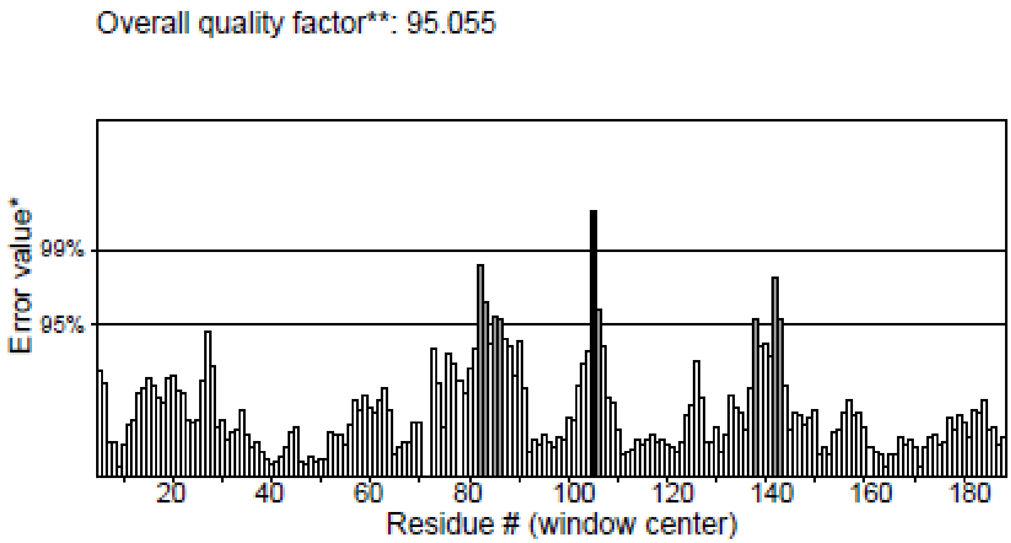

b)

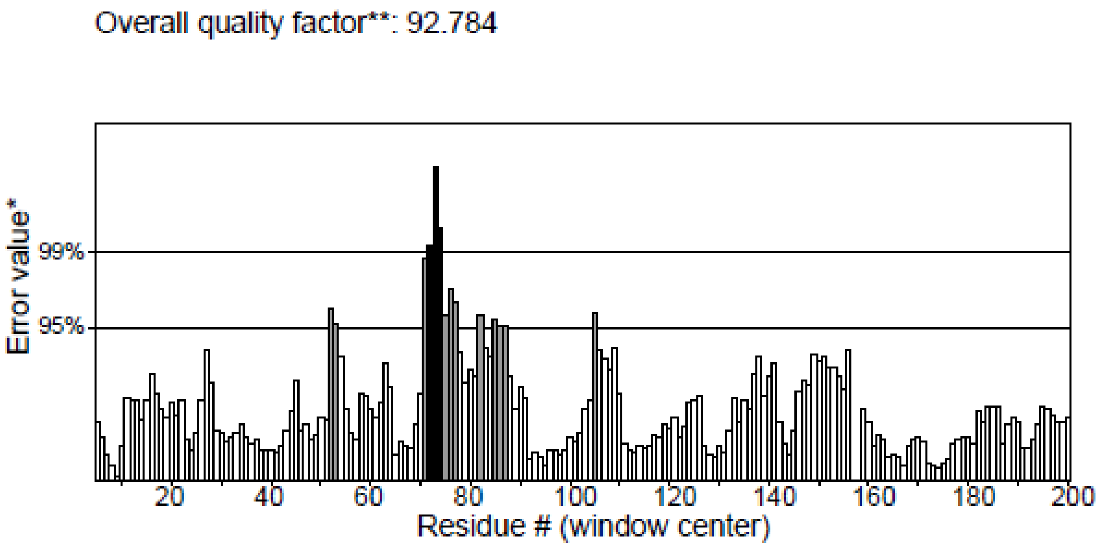

ERRAT results of a) ICP 7035 and b) ICPL 84023 showing overall quality factor.

a)

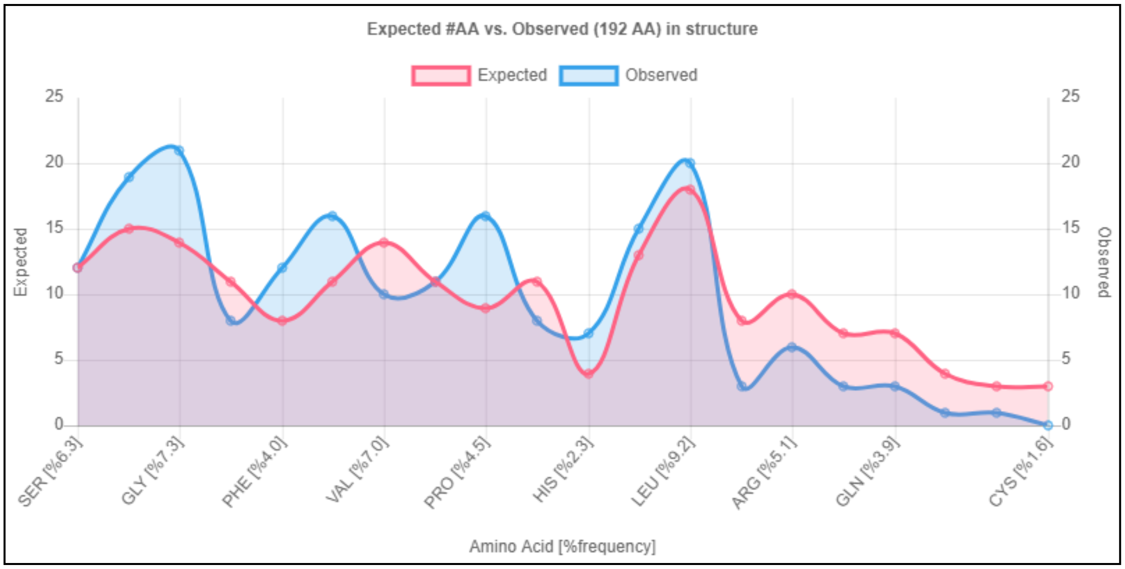

b)

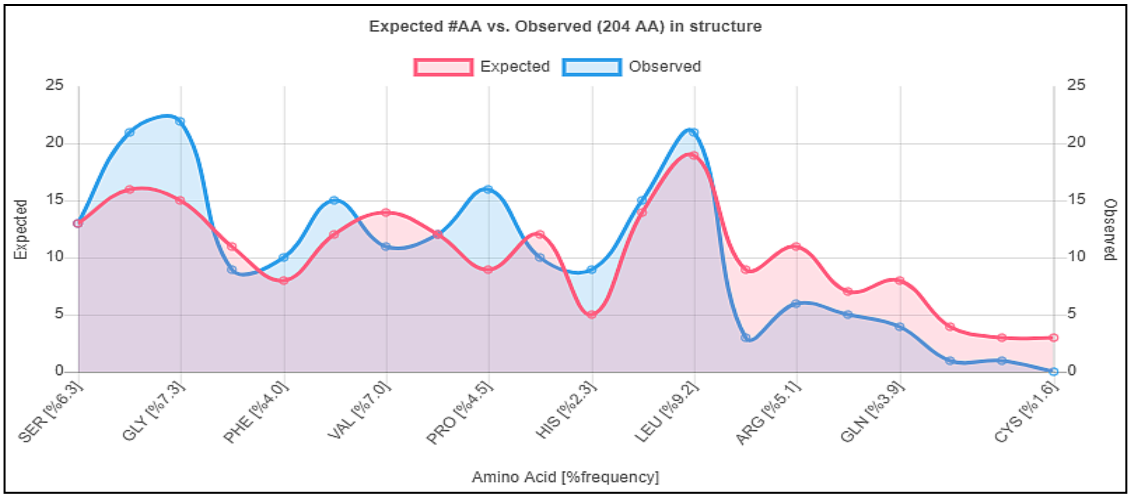

SAVES result of a) ICP 7035 and b) ICPL 84023 showing expected and observed amino acid distribution.

a)

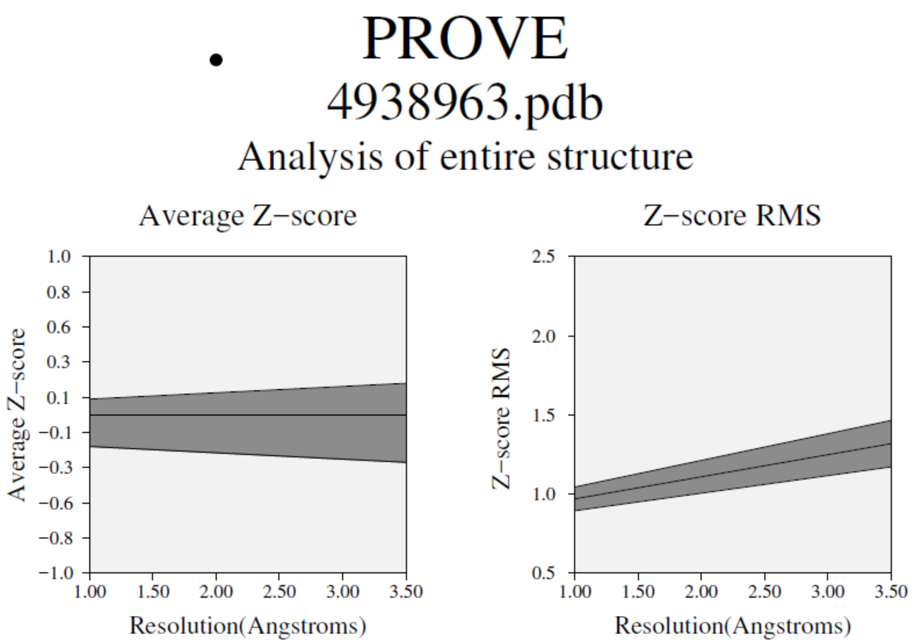

b)

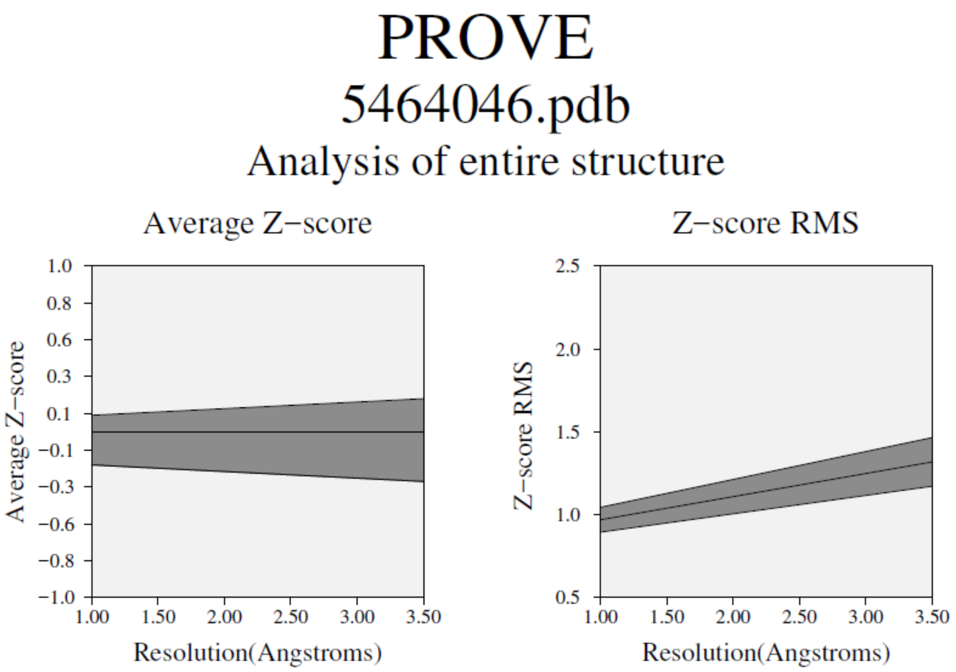

PROVE results for a) ICP 7035 and b) ICPL 84023

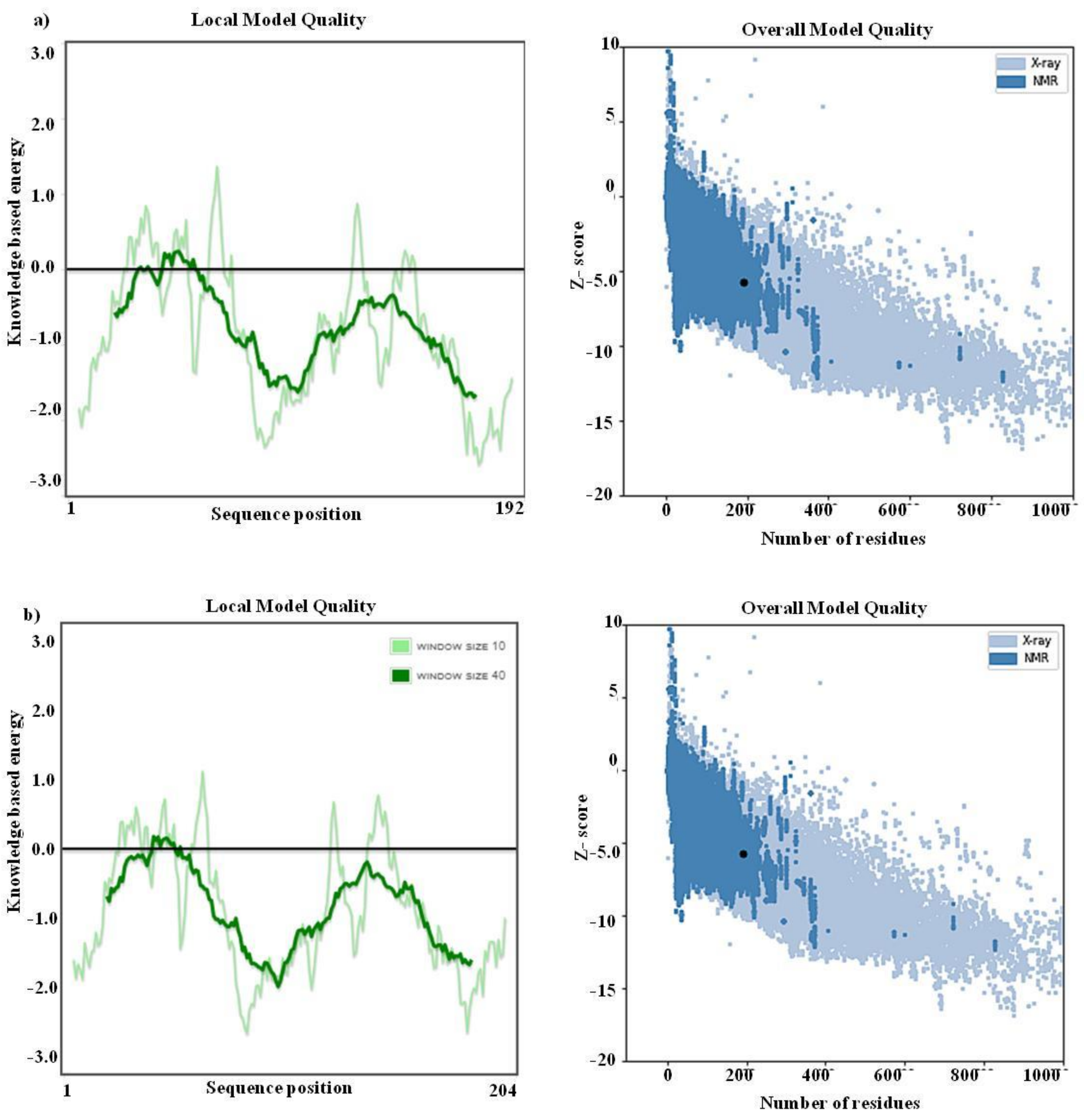

Local and overall model quality of a) ICP 7035 and b) ICPL 84023 structure using ProSA after energy minimization.
