# Supplementary material for: In silico characterization and homology modeling of cytosolic APX gene predicts novel glycine residue modulating waterlogging stress response in pigeon pea
Source: PeerJ. 2021 May 12;9:e10888. doi: 10.7717/peerj.10888 (PMC8123230; doi:10.7717/peerj.10888)
Supplement: Supplemental Information 6 [file peerj-09-10888-s006.pdf]

Fig. S6: Structural view of helix, beta turn, gamma turn and distorted geometry in two pigeon pea genotypes ICP 7035 and ICPL 84023

A): Structural view of 14 helix in ICP 7035

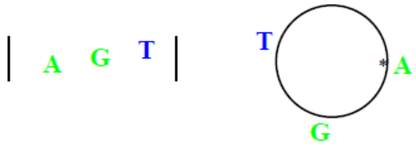

Helix 1. Ala2-Thr4
Sequence: AGT
Length: 3 residues

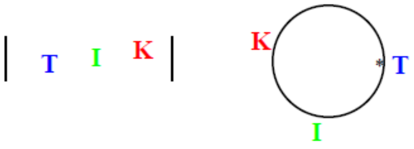

Helix 2. Thr17-Lys19
Sequence: T I K
Length: 3 residues

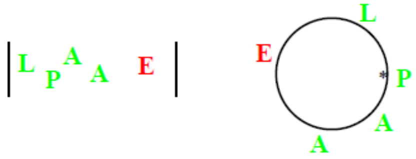

Helix 3. Pro21-Ala25
Sequence: P A E L A
Length: 5 residues

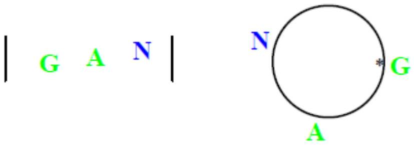

Helix 4. Gly27-Asn29
Sequence: GAN
Length: 3 residues

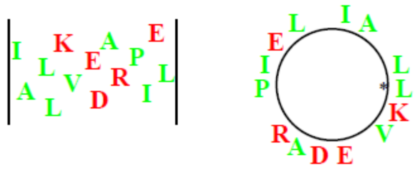

Helix 5. Leu32-Glu45
Sequence: LD I AVRLLE P I KAE
Length: 14 residues

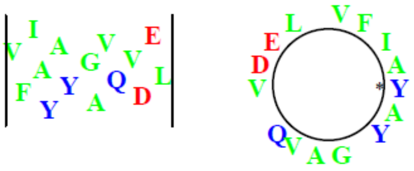

Helix 6. Tyr51-Ile65
Sequence: YAD FYQLAGVVAVE I
Length: 15 residues

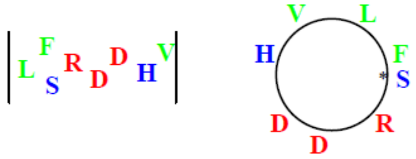

Helix 7. Ser96-Phe103
Sequence: S DHLRDV F
Length: 8 residues

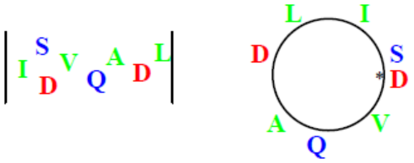

Helix 8. Asp111-Ser118
Sequence: DQD I VALS
Length: 8 residues

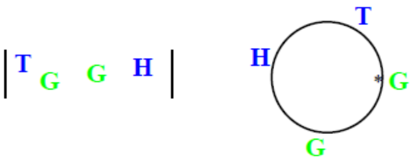

Helix 9. Gly119-Thr122
Sequence: GGHT
Length: 4 residues

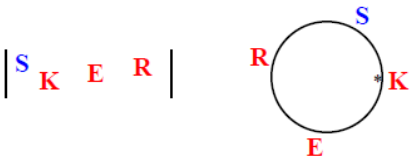

Helix 10. Lys128-Ser131
Sequence: KERS
Length: 4 residues

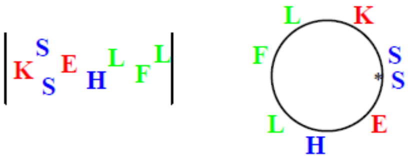

Helix 11. Ser147-Ser154
Sequence: SHFKELLS
Length: 8 residues

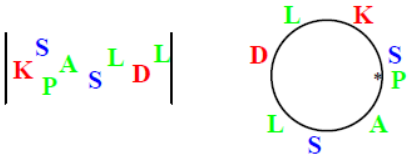

Helix 12. Pro164-Ser171
Sequence: PSDKALLS
Length: 8 residues

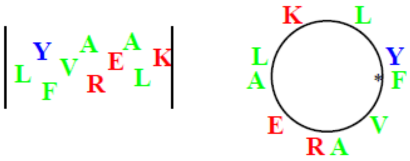

Helix 13. Phe175-Ala184
Sequence: FRLLVEKYAA
Length: 10 residues

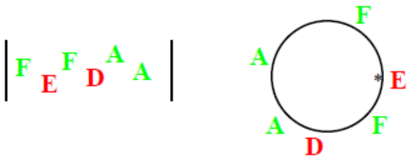

Helix 14. Glu186-Ala191
Sequence: EDAFFA
Length: 6 residues

B): Structural view of 14 helix in ICPL 84023

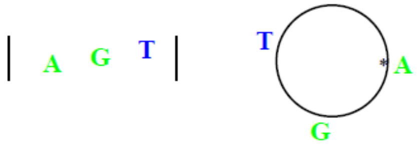

Helix 1. Ala2-Thr4
Sequence: AGT
Length: 3 residues

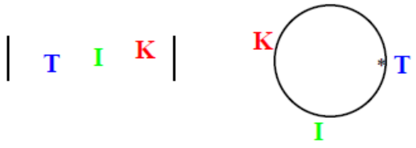

Helix 2. Thr17-Lys19
Sequence: TIK
Length: 3 residues

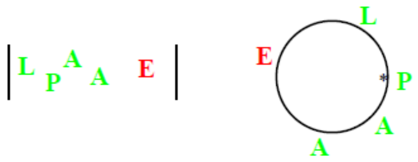

Helix 3. Pro21-Ala25
Sequence: PAELA
Length: 5 residues

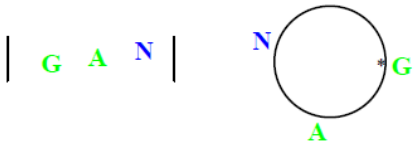

Helix 4. Gly27-Asn29
Sequence: GAN
Length: 3 residues

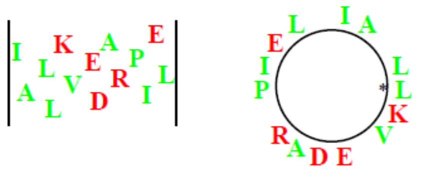

Helix 5. Leu32-Glu45
Sequence: LDIAVRLLEPIKAE
Length: 14 residues

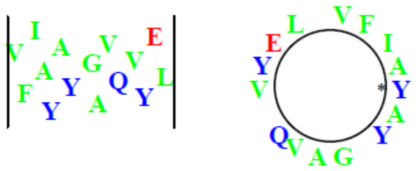

Helix 6. Tyr51-Ile65
Sequence: YAYFYQLAGVVAVEI
Length: 15 residues

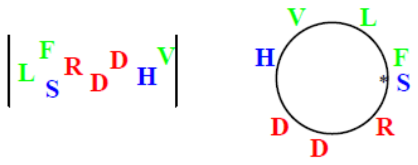

Helix 7. Ser96-Phe103
Sequence: SDHLRDVF
Length: 8 residues

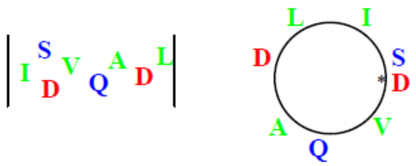

Helix 8. Asp111-Ser118
Sequence: DQDIVALS
Length: 8 residues

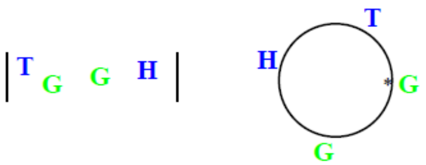

Helix 9. Gly119-Thr122
Sequence: G G H T
Length: 4 residues

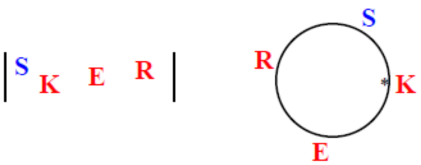

Helix 10. Lys128-Ser131
Sequence: K E R S
Length: 4 residues

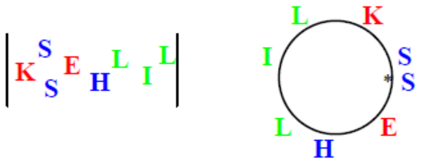

Helix 11. Ser147-Ser154
Sequence: S H I K E L L S
Length: 8 residues

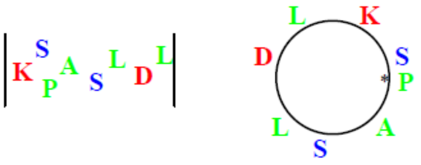

Helix 12. Pro164-Ser171
Sequence: P S D K A L L S
Length: 8 residues

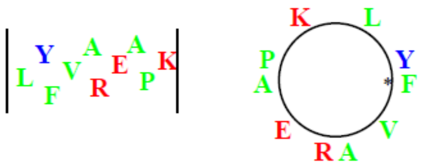

Helix 13. Phe175-Ala184
Sequence: F R P L V E K Y A A
Length: 10 residues

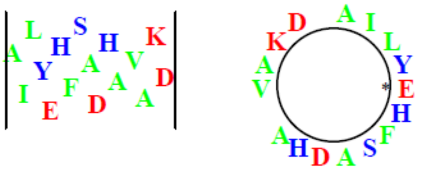

Helix 14. Glu186-Ser201
Sequence: E D A I F A D Y A V A H H K L S
Length: 16 residues

C) beta turn in ICP 7035

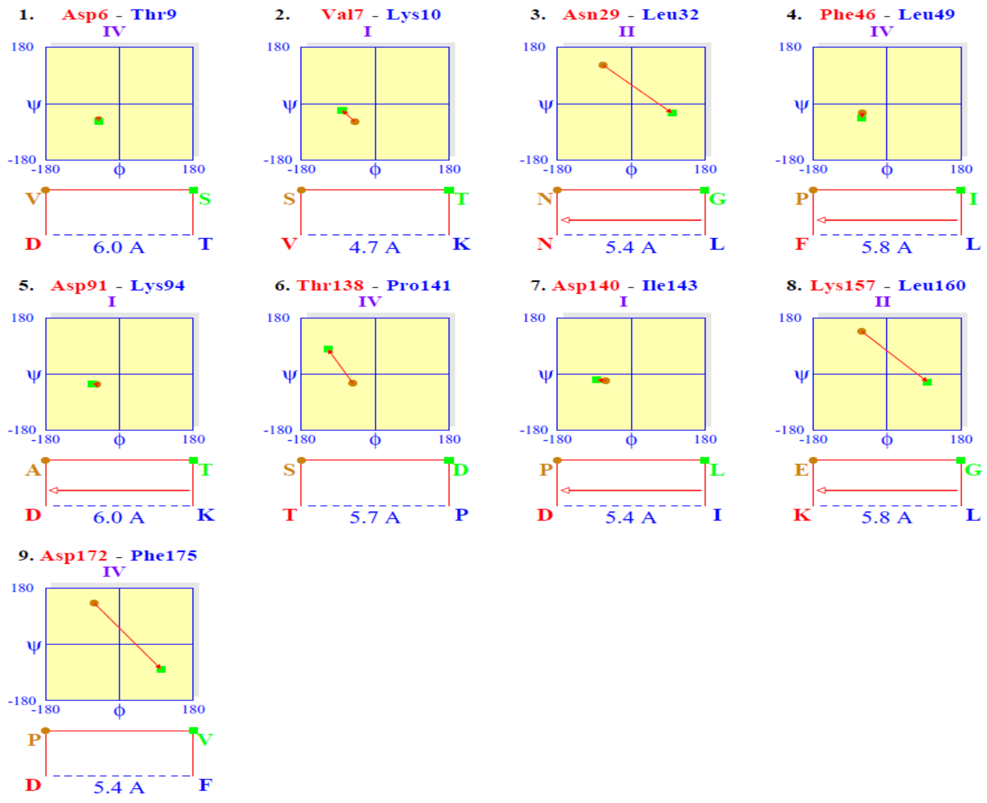

beta turn in ICPL 84023

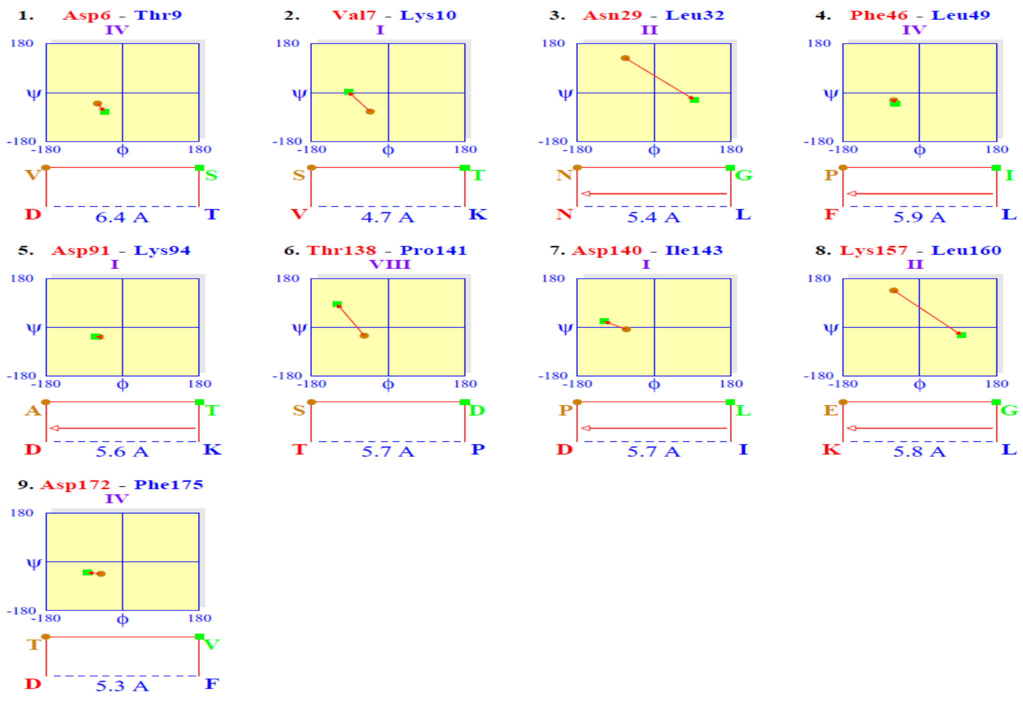

D) Distorted geometry of ICP 7035

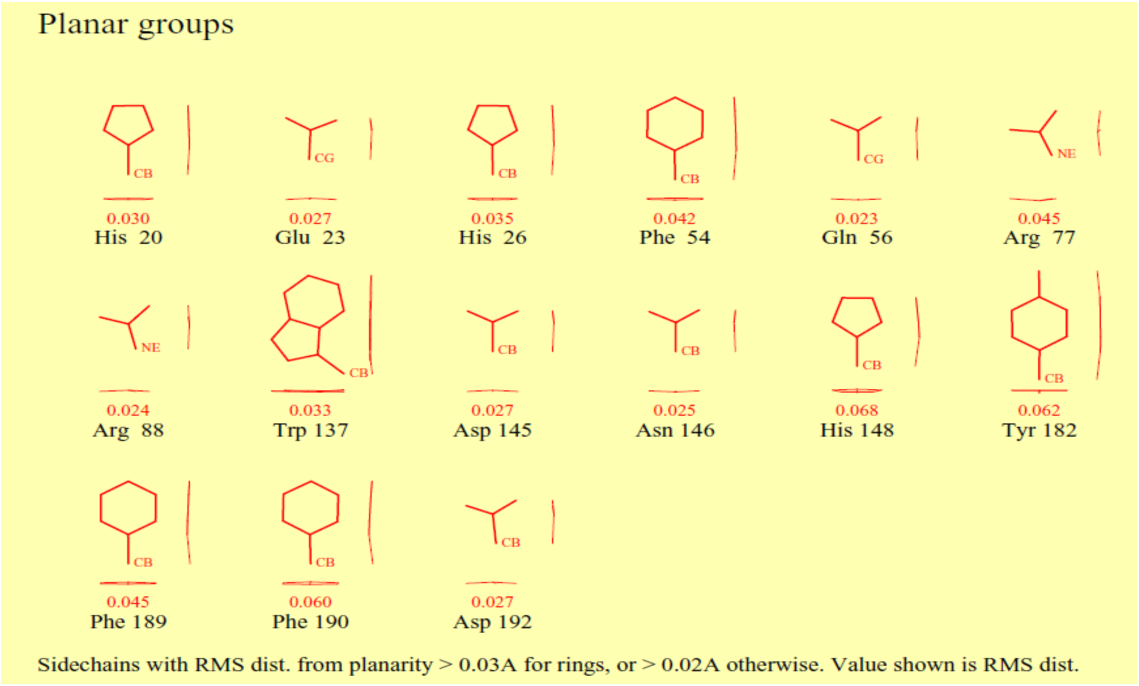

Distorted geometry of ICPL 84023

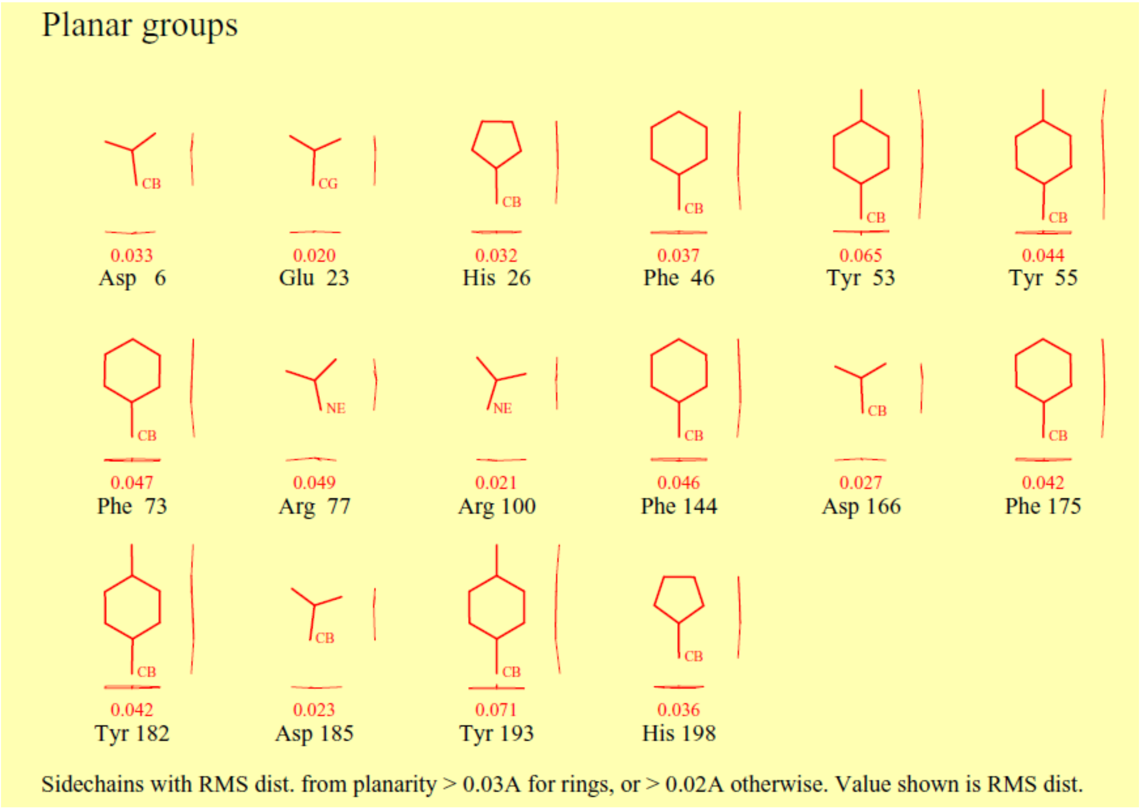

E) Gamma turn of ICP 7035 and ICPL 84023

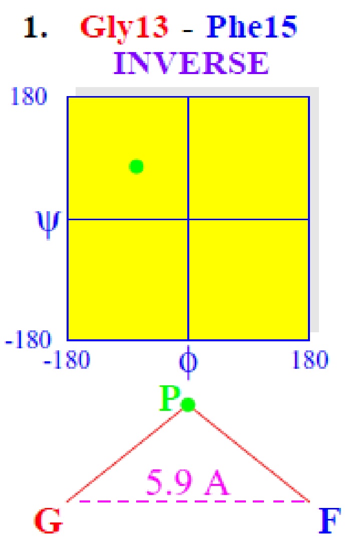

ICP 7035

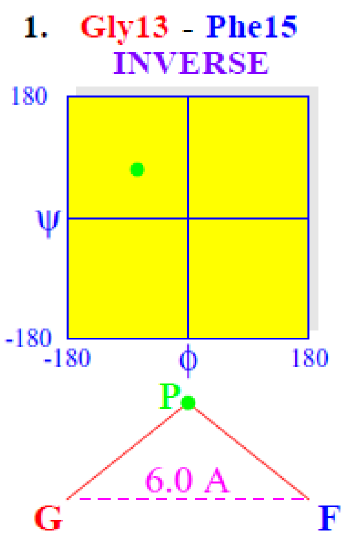

ICPL 84023
