# Supplementary material for: In silico characterization and homology modeling of cytosolic APX gene predicts novel glycine residue modulating waterlogging stress response in pigeon pea
Source: PeerJ. 2021 May 12;9:e10888. doi: 10.7717/peerj.10888 (PMC8123230; doi:10.7717/peerj.10888)
Supplement: Supplemental Information 7 [file peerj-09-10888-s007.docx]

| **Molecular and physiochemical profiling** | **ICP 7035** | **ICPL 84023** |
| --- | --- | --- |
| Number of amino acids | 192 | 204 |
| Molecular weight | 20518.97 | 21792.47 |
| Theoretical pI | 4.77 | 5.09 |
| Total number of negatively charged residues (Asp + Glu) | 31 | 30 |
| Total number of positively charged residues (Arg + Lys) | 17 | 18 |
| Atomic composition | Carbon 926  Hydrogen 1420  Nitrogen 242  Oxygen 284  Sulfur 1 | Carbon 983  Hydrogen 1516  Nitrogen 260  Oxygen 299  Sulfur 1 |
| Formula | C926H1420N242O284S1 | C983H1516N260O299S1 |
| Total number of atoms | 2873 | 3059 |
| Extinction coefficients in H2O(280nm) | 9970 | 12950 |
| Estimated half-life:  The N-terminal of the sequence considered is S (Ser) | 1.9 hours (mammalian reticulocytes, in vitro).  >20 hours (yeast, in vivo).  >10 hours (Escherichia coli, in vivo) | 1.9 hours (mammalian reticulocytes, in vitro).  >20 hours (yeast, in vivo).  >10 hours (Escherichia coli, in vivo) |
| Instability index (II) | 31.16 (Stable) | 35.38 (Stable) |
| Aliphatic index | 81.88 | 85.20 |
| Grand average of hydropathicity (GRAVY) | -0.271 | -0.254 |
| Amino acid composition | Amino Number Percentage  acid  Ala (A) 19 9.9%  Arg (R) 6 3.1%  Asn (N) 3 1.6%  Asp (D) 16 8.3%  Cys (C) 0 0.0%  Gln (Q) 3 1.6%  Glu (E) 15 7.8%  Gly (G) 21 10.9%  His (H) 7 3.6%  Ile (I) 8 4.2%  Leu (L) 20 10.4%  Lys (K) 11 5.7%  Met (M) 1 0.5%  Phe (F) 12 6.2%  Pro (P) 16 8.3%  Ser (S) 12 6.2%  Thr (T) 8 4.2%  Trp (W) 1 0.5%  Tyr (Y) 3 1.6%  Val (V) 10 5.2% | Amino Number Percentage  acid  Ala (A) 21 10.3%  Arg (R) 6 2.9%  Asn (N) 3 1.5%  Asp (D) 15 7.4%  Cys (C) 0 0.0%  Gln (Q) 4 2.0%  Glu (E) 15 7.4%  Gly (G) 22 10.8%  His (H) 9 4.4%  Ile (I) 10 4.9%  Leu (L) 21 10.3%  Lys (K) 12 5.9%  Met (M) 1 0.5%  Phe (F) 10 4.9%  Pro (P) 16 7.8%  Ser (S) 13 6.4%  Thr (T) 9 4.4%  Trp (W) 1 0.5%  Tyr (Y) 5 2.5%  Val (V) 11 5.4% |

**Table S1: Molecular and physiochemical properties of *APX* protein in two pigeon pea genotypes**
