# Supplementary material for: In silico characterization and homology modeling of cytosolic APX gene predicts novel glycine residue modulating waterlogging stress response in pigeon pea
Source: PeerJ. 2021 May 12;9:e10888. doi: 10.7717/peerj.10888 (PMC8123230; doi:10.7717/peerj.10888)
Supplement: Supplemental Information 8 [file peerj-09-10888-s008.docx]

**Table S2**

**Individual hydropathy index of all 20 amino acids using the** [**Hphob. / Kyte & Doolittle**](https://web.expasy.org/protscale/pscale/Hphob.Doolittle.html) **scale  in ICP 7035**

| Ala: 1.800 | Arg: -4.500 | Asn: -3.500 | Asp: -3.500 | Cys: 2.500 | Gln: -3.500 |
| --- | --- | --- | --- | --- | --- |
| Glu: -3.500 | Gly: -0.400 | His: -3.200 | Ile: 4.500 | Leu: 3.800 | Lys: -3.900 |
| Met: 1.900 | Phe: 2.800 | Pro: -1.600 | Ser: -0.800 | Thr: -0.700 | Trp: -0.900 |
| Tyr: -1.300 | Val: 4.200, -3.500, -3.500 ,-0.490 |  |  |  |  |

**Individual hydropathy index of all 20 amino acids using the** [**Hphob. / Kyte & Doolittle**](https://web.expasy.org/protscale/pscale/Hphob.Doolittle.html) **scale  in ICPL84023**

| Ala: 1.800 | Arg: -4.500 | Asn: -3.500 | Asp: -3.500 | Cys: 2.500 | Gln: -3.500 |
| --- | --- | --- | --- | --- | --- |
| Glu: -3.500 | Gly: -0.400 | His: -3.200 | Ile: 4.500 | Leu: 3.800 | Lys: -3.900 |
| Met: 1.900 | Phe: 2.800 | Pro: -1.600 | Ser: -0.800 | Thr: -0.700 | Trp: -0.900 |
| Tyr: -1.300 | Val: 4.200, -3.500, -3.500 ,-0.490 |  |  |  |  |
