# Supplementary material for: In silico characterization and homology modeling of cytosolic APX gene predicts novel glycine residue modulating waterlogging stress response in pigeon pea
Source: PeerJ. 2021 May 12;9:e10888. doi: 10.7717/peerj.10888 (PMC8123230; doi:10.7717/peerj.10888)
Supplement: Supplemental Information 9 [file peerj-09-10888-s009.docx]

**>maker_00053869 P48534 (unpublished cytosolic APX sequence of *Cymopsis tetragonoloba*)**

| **Description** | L-ascorbate peroxidase, cytosolic (AP) (EC 1.11.1.11) (PsAPx01) APX1 APPX1 Pisum sativum (Garden pea) 1.11.1.11 |
| --- | --- |
| **Gene ontology** | GO:0005737; GO:0006979; GO:0016688; GO:0020037; GO:0042744; GO:0046872 cytoplasm [GO:0005737]; heme binding [GO:0020037]; L-ascorbate peroxidase activity [GO:0016688]; metal ion binding [GO:0046872]; hydrogen peroxide catabolic process [GO:0042744]; response to oxidative stress [GO:0006979] hydrogen peroxide catabolic process [GO:0042744]; response to oxidative stress [GO:0006979] cytoplasm [GO:0005737] L-ascorbate-peroxidase activity [GO:0016688]; heme binding [GO:0020037]; metal ion binding [GO:0046872] |
| **Sequence** | ATGGCAATTGGAAGAGGCCTTCTTTCTCACCACTCCTTCAAGGTAAGAGTGGTAAAAAATGAAGGGTTTGTAGTCCAGGGCCCTCAACAGATTGATGTAACATCTTTTCAATTATTTTGATGGTAGAAGTCAGCATAGCTCAAAATAGGGAATTCCTGTTTAATAGGCTCAACAATCTTACAGTGATGTCAAGCCCGTTGTTAGCACCGTGGGCAAGTTCAGAAGGGTGCTTGATAGTTCCGAATGGACCACCCGTCTTCGTTTTATGGTTAAATGTCCCAGCCAGTGCCATCTACAAATATACCATGAAGCTATACTAAGCATCAAAGGAGCACATCTCTTCTCAGTGATGAAATCTCTGAGCTTCCTCTTGGCCTTCTCAACAGCCTTTTTGGTAATCTGCACTCACAGTTGGGTAGGACTTGCCCATAGCTAA |
