# Supplementary material for: In silico characterization and homology modeling of cytosolic APX gene predicts novel glycine residue modulating waterlogging stress response in pigeon pea
Source: PeerJ. 2021 May 12;9:e10888. doi: 10.7717/peerj.10888 (PMC8123230; doi:10.7717/peerj.10888)
Supplement: Supplemental Information 10 [file peerj-09-10888-s010.docx]

**Table S3: Summary of steriochemical properties of ICP 7035 using PROCHECK**

| **Steriochemical properties of ICP 7035** | | **Steriochemical properties of ICPL 84023** | |
| --- | --- | --- | --- |
| **Ramachandran plot** | 95.4% core  3.9% allow  0.0% gener  0.7% disall | **Ramachandran plot** | 93.3% core  6.1% allow  0.0% gener  0.6% disall |
| **All Ramachandrans** | 2 labelled  Residues | **All Ramachandrans** | 3 labelled  Residues |
| **Chi1-chi2 plots** | 0 labelled residues | **Chi1-chi2 plots** | 1 labelled residues |
| **Side-chain parameters** | 5 better  0 inside  0 worse | **Side-chain parameters** | 5 better  0 inside  0 worse |
| **Residue properties** | Max.deviation: 4.0  Bad contacts: 1  Bond len/angle: 3.3  Morris et al class: 1,1,2 | **Residue properties** | Max.deviation: 17.4  Bad contacts: 1  Bond len/angle: 3.8  Morris et al class: 1,1,2 |
| **G-factors** | Dihedrals: -0.08  Covalent: 0.29  Overall: 0.08 | **G-factors** | Dihedrals: -0.08  Covalent: 0.23  Overall: 0.05 |
| **Planar groups** | 77.3% within limits  22.7% highlighted  3 off graph | **Planar groups** | 76.5% within limits  23.5% highlighted  2 off graph |
