# Supplementary material for: In silico characterization and homology modeling of cytosolic APX gene predicts novel glycine residue modulating waterlogging stress response in pigeon pea
Source: PeerJ. 2021 May 12;9:e10888. doi: 10.7717/peerj.10888 (PMC8123230; doi:10.7717/peerj.10888)
Supplement: Supplemental Information 11 [file peerj-09-10888-s011.docx]

| **Table S4: Detailed secondary structure in ICP 7035 showing a) Secondary structure summary b) beta sheet c) beta hairpins, d) beta strands, e) helices, f) helix-helix interaction, g) beta turns, h) gamma turns**  **a) Secondary structure summary**    \| **** \| \| \| \| \| \| \| \| \| \| --- \| --- \| --- \| --- \| --- \| --- \| --- \| --- \| --- \| \| **Strand** \| **** \| **Alpha helix** \| **** \| **3-10 helix** \| **** \| **Other** \| **** \| **Total residues** \| \| **** \| \| \| \| \| \| \| \| \| \| **4 (2.1%)** \|  \| **86 (44.6%)** \|  \| **12 (6.2%)** \|  \| **91 (47.2%)** \|  \| **193** \|   [**b) Beta sheet**](http://www.ebi.ac.uk/thornton-srv/databases/cgi-bin/pdbsum/GetPage.pl?pdbcode=e493&pdb_type=UPLOAD&code=063658&template=protein.html&o=SHEETS&l=1&c=2&pdb_type=UPLOAD&code=063658&chain=NONE)    \| **** \| \| \| \| \| \| \| \| \| \| --- \| --- \| --- \| --- \| --- \| --- \| --- \| --- \| --- \| \| **Sheet** \| **** \| **No.   strands** \| **** \| **Type** \| **** \| **Barrel** \| **** \| **Topology** \| \| **** \| \| \| \| \| \| \| \| \| \| **A** \|  \| **2** \|  \| **Antiparallel** \|  \| **N** \|  \| **1** \| \| **** \| \| \| \| \| \| \| \| \|   **c) Beta hairpins** |
| --- | --- | --- | --- | --- | --- | --- | --- | --- | --- | --- | --- | --- | --- | --- | --- | --- | --- | --- | --- | --- | --- | --- | --- | --- | --- | --- | --- | --- | --- | --- | --- | --- | --- | --- | --- | --- | --- | --- | --- | --- | --- | --- | --- | --- | --- | --- | --- | --- | --- | --- | --- | --- | --- | --- | --- | --- | --- | --- | --- | --- | --- | --- | --- | --- | --- | --- | --- | --- | --- | --- | --- | --- | --- | --- | --- | --- | --- | --- | --- | --- | --- |
|  |
| \|  \| \| \| \| \| \| \| \| \| \| \| \| \| \| \| \| --- \| --- \| --- \| --- \| --- \| --- \| --- \| --- \| --- \| --- \| --- \| --- \| --- \| --- \| --- \| \|  \|  \| **Strand 1** \| \| \| \| \|  \| **Strand 2** \| \| \| \| \|  \| **Hairpin** \| \|  \|  \|  \| \| \| \| \|  \|  \| \| \| \| \|  \|  \| \| **No.** \|  \| **Start** \|  \| **End** \|  \| **Length** \|  \| **Start** \|  \| **End** \|  \| **Length** \|  \| **class** \| \|  \| \| \| \| \| \| \| \| \| \| \| \| \| \| \| \| 1. \|  \| Ala125 \|  \| Ala126 \|  \| 2 \|  \| Gly135 \|  \| Pro136 \|  \| 2 \|  \| 8:8 \| \|  \| \| \| \| \| \| \| \| \| \| \| \| \| \| \| |
|  |
|  |
| **d) Beta strands** |
|  |
| \|  \| \| \| \| \| \| \| \| \| \| \| \| \| --- \| --- \| --- \| --- \| --- \| --- \| --- \| --- \| --- \| --- \| --- \| --- \| \| **No.** \|  \| **Start** \|  \| **End** \|  \| **Sheet** \|  \| **No. residue** \|  \| **Edge** \| **Sequence** \|  \| \|  \| \| \| \| \| \| \| \| \| \| \| \| \| 1. \|  \| Ala125 \|  \| Ala126 \|  \| A \|  \| 2 \|  \| Yes \| AA \|  \| \| 2. \|  \| Gly135 \|  \| Pro136 \|  \| A \|  \| 2 \|  \| Yes \| GP \|  \| \|  \| \| \| \| \| \| \| \| \| \| \| \| |
|  |
|  |
| **e) Table of helices** |
|  |
| \|  \| \| \| \| \| \| \| \| \| \| \| \| \| \| \| \| \| \| \| \| \| \| \| \| --- \| --- \| --- \| --- \| --- \| --- \| --- \| --- \| --- \| --- \| --- \| --- \| --- \| --- \| --- \| --- \| --- \| --- \| --- \| --- \| --- \| --- \| --- \| \| **No.** \|  \| **Start** \|  \| **End** \|  \| **Type** \|  \| **No. resid** \|  \| **Length** \|  \| **Unit rise** \|  \| **Residues per turn** \|  \| **Pitch** \|  \| **Deviation from ideal** \|  \| **Sequence** \|  \|  \| \|  \| \| \| \| \| \| \| \| \| \| \| \| \| \| \| \| \| \| \| \| \| \| \| \| *1. \|  \| Ala2 \|  \| Thr4 \|  \| G \|  \| 3 \|  \| - \|  \| - \|  \| - \|  \| - \|  \| - \|  \| AGT \|  \|  \| \| *2. \|  \| Thr17 \|  \| Lys19 \|  \| G \|  \| 3 \|  \| - \|  \| - \|  \| - \|  \| - \|  \| - \|  \| TIK \|  \|  \| \| *3. \|  \| Pro21 \|  \| Ala25 \|  \| H \|  \| 5 \|  \| 8.07 \|  \| 1.48 \|  \| 3.59 \|  \| 5.30 \|  \| 21.4 \|  \| PAELA \|  \|  \| \| *4. \|  \| Gly27 \|  \| Asn29 \|  \| G \|  \| 3 \|  \| - \|  \| - \|  \| - \|  \| - \|  \| - \|  \| GAN \|  \|  \| \|  \| \| \| \| \| \| \| \| \| \| \| \| \| \| \| \| \| \| \| \| \| \| \| \| 5. \|  \| Leu32 \|  \| Glu45 \|  \| H \|  \| 14 \|  \| 22.57 \|  \| 1.58 \|  \| 4.01 \|  \| 6.34 \|  \| 31.1 \|  \| LDIAVRLLEPIKAE \|  \|  \| \| 6. \|  \| Tyr51 \|  \| Ile65 \|  \| H \|  \| 15 \|  \| 22.31 \|  \| 1.45 \|  \| 3.67 \|  \| 5.32 \|  \| 11.3 \|  \| YADFYQLAGVVAVEI \|  \|  \| \| 7. \|  \| Ser96 \|  \| Phe103 \|  \| H \|  \| 8 \|  \| 11.71 \|  \| 1.41 \|  \| 3.82 \|  \| 5.41 \|  \| 13.4 \|  \| SDHLRDVF \|  \|  \| \| 8. \|  \| Asp111 \|  \| Ser118 \|  \| H \|  \| 8 \|  \| 12.87 \|  \| 1.56 \|  \| 3.59 \|  \| 5.58 \|  \| 13.2 \|  \| DQDIVALS \|  \|  \| \|  \| \| \| \| \| \| \| \| \| \| \| \| \| \| \| \| \| \| \| \| \| \| \| \| 9. \|  \| Gly119 \|  \| Thr122 \|  \| G \|  \| 4 \|  \| 8.12 \|  \| 1.93 \|  \| 3.20 \|  \| 6.17 \|  \| 38.0 \|  \| GGHT \|  \|  \| \| 10. \|  \| Lys128 \|  \| Ser131 \|  \| H \|  \| 4 \|  \| 4.79 \|  \| 1.17 \|  \| 4.28 \|  \| 4.98 \|  \| 41.4 \|  \| KERS \|  \|  \| \| 11. \|  \| Ser147 \|  \| Ser154 \|  \| H \|  \| 8 \|  \| 12.55 \|  \| 1.48 \|  \| 3.74 \|  \| 5.54 \|  \| 12.9 \|  \| SHFKELLS \|  \|  \| \| 12. \|  \| Pro164 \|  \| Ser171 \|  \| H \|  \| 8 \|  \| 12.88 \|  \| 1.54 \|  \| 3.62 \|  \| 5.59 \|  \| 17.4 \|  \| PSDKALLS \|  \|  \| \|  \| \| \| \| \| \| \| \| \| \| \| \| \| \| \| \| \| \| \| \| \| \| \| \| 13. \|  \| Phe175 \|  \| Ala184 \|  \| H \|  \| 10 \|  \| 15.76 \|  \| 1.53 \|  \| 3.59 \|  \| 5.50 \|  \| 2.6 \|  \| FRLLVEKYAA \|  \|  \| \| 14. \|  \| Glu186 \|  \| Ala191 \|  \| H \|  \| 6 \|  \| 9.67 \|  \| 1.53 \|  \| 3.50 \|  \| 5.33 \|  \| 4.7 \|  \| EDAFFA \|  \|  \| \|  \| \| \| \| \| \| \| \| \| \| \| \| \| \| \| \| \| \| \| \| \| \| \| |
|  |
|  |
| **f) Helix-helix interactions** |
|  |
| \|  \|  \|  \|  \| \|  \| **Helix** \| \|  \|  \|  \|  \|  \| **Interaction** \| \|  \| **No. interacting residues** \| \| \| \| \| \| --- \| --- \| --- \| --- \| --- \| --- \| --- \| --- \| --- \| --- \| --- \| --- \| --- \| --- \| --- \| --- \| --- \| --- \| --- \| --- \| --- \| \|  \|  \|  \|  \| \|  \|  \| \|  \|  \|  \|  \|  \|  \| \|  \|  \| \| \| \| \| \| **No.** \|  \|  \| **Helices** \| \|  \| **types** \| \|  \| **Distance (Å)** \|  \| **Angle (°)** \|  \| **type** \| \|  \| **Total** \|  \| **Helix 1** \|  \| **Helix 2** \| \| 1. \|  \|  \| 3 \| 5 \|  \| H \| H \|  \| 9.7 \|  \| 153.5 \|  \| c \| n \|  \| 3 \|  \| 1 \|  \| 3 \| \| 2. \|  \|  \| 5 \| 6 \|  \| H \| H \|  \| 10.9 \|  \| 108.8 \|  \| I \| I \|  \| 10 \|  \| 6 \|  \| 4 \| \| 3. \|  \|  \| 5 \| 10 \|  \| H \| H \|  \| 9.9 \|  \| -98.2 \|  \| N \| C \|  \| 2 \|  \| 2 \|  \| 1 \| \| 4. \|  \|  \| 7 \| 8 \|  \| H \| H \|  \| 9.9 \|  \| 107.9 \|  \| I \| I \|  \| 6 \|  \| 3 \|  \| 4 \| \| 5. \|  \|  \| 7 \| 12 \|  \| H \| H \|  \| 8.0 \|  \| -90.5 \|  \| N \| I \|  \| 5 \|  \| 2 \|  \| 4 \| \| 6. \|  \|  \| 7 \| 13 \|  \| H \| H \|  \| 12.0 \|  \| 86.2 \|  \| I \| N \|  \| 3 \|  \| 3 \|  \| 1 \| \| 7. \|  \|  \| 8 \| 9 \|  \| H \| G \|  \| 0.0 \|  \| -9.2 \|  \| C \| I \|  \| 11 \|  \| 5 \|  \| 4 \| \| 8. \|  \|  \| 8 \| 11 \|  \| H \| H \|  \| 8.4 \|  \| 93.5 \|  \| c \| n \|  \| 5 \|  \| 3 \|  \| 3 \| \| 9. \|  \|  \| 8 \| 12 \|  \| H \| H \|  \| 11.3 \|  \| -140.5 \|  \| I \| I \|  \| 4 \|  \| 3 \|  \| 2 \| \| 10. \|  \|  \| 8 \| 13 \|  \| H \| H \|  \| 9.5 \|  \| -49.3 \|  \| I \| I \|  \| 12 \|  \| 5 \|  \| 4 \| \| 11. \|  \|  \| 8 \| 14 \|  \| H \| H \|  \| 9.4 \|  \| -110.1 \|  \| I \| C \|  \| 3 \|  \| 3 \|  \| 1 \| \| 12. \|  \|  \| 9 \| 11 \|  \| G \| H \|  \| 6.9 \|  \| 95.5 \|  \| I \| N \|  \| 5 \|  \| 3 \|  \| 3 \| \| 13. \|  \|  \| 9 \| 12 \|  \| G \| H \|  \| 11.3 \|  \| -131.8 \|  \| N \| I \|  \| 2 \|  \| 1 \|  \| 2 \| \| 14. \|  \|  \| 9 \| 14 \|  \| G \| H \|  \| 10.4 \|  \| -117.2 \|  \| n \| c \|  \| 3 \|  \| 3 \|  \| 1 \| \| 15. \|  \|  \| 11 \| 12 \|  \| H \| H \|  \| 10.7 \|  \| -80.9 \|  \| I \| I \|  \| 8 \|  \| 4 \|  \| 4 \| \| 16. \|  \|  \| 11 \| 13 \|  \| H \| H \|  \| 7.6 \|  \| -118.8 \|  \| I \| I \|  \| 10 \|  \| 4 \|  \| 6 \| \| 17. \|  \|  \| 11 \| 14 \|  \| H \| H \|  \| 9.6 \|  \| 113.6 \|  \| N \| I \|  \| 1 \|  \| 1 \|  \| 1 \| \| 18. \|  \|  \| 12 \| 13 \|  \| H \| H \|  \| 4.3 \|  \| -99.5 \|  \| c \| n \|  \| 7 \|  \| 4 \|  \| 3 \| \| 19. \|  \|  \| 13 \| 14 \|  \| H \| H \|  \| 4.9 \|  \| -123.7 \|  \| c \| n \|  \| 5 \|  \| 3 \|  \| 3 \| |
|  |
|  |
| **g) Beta turns** |
|  |
| \|  \| \| \| \| \| \| \| \| \| \| \| \| \| \| \| \| \| \| \| \| \| \| \| \| --- \| --- \| --- \| --- \| --- \| --- \| --- \| --- \| --- \| --- \| --- \| --- \| --- \| --- \| --- \| --- \| --- \| --- \| --- \| --- \| --- \| --- \| --- \| \|  \|  \|  \|  \|  \|  \| **Turn** \|  \| **Residue i+1** \| \| \| \| \|  \| **Residue i+2** \| \| \| \| \|  \| **i to i+3** \|  \|  \| \|  \|  \|  \|  \|  \|  \|  \|  \|  \| \| \| \| \|  \|  \| \| \| \| \|  \|  \|  \|  \| \| **No.** \|  \| **Turn** \|  \| **Sequence*** \|  \| **type** \|  \| **Phi** \|  \| **Psi** \|  \| **Chi1** \|  \| **Phi** \|  \| **Psi** \|  \| **Chi1** \|  \| **CA-dist** \|  \| **H-bond** \| \|  \| \| \| \| \| \| \| \| \| \| \| \| \| \| \| \| \| \| \| \| \| \| \| \| *  1. \|  \| Asp6-Thr9 \|  \| DVST \|  \| IV \|  \| -50.2 \|  \| -51.4 \|  \| -171.0 \|  \| -49.6 \|  \| -57.4 \|  \| -47.0 \|  \| 6.0 \|  \| Yes \| \| *  2. \|  \| Val7-Lys10 \|  \| VSTK \|  \| I \|  \| -49.6 \|  \| -57.4 \|  \| -47.0 \|  \| -80.7 \|  \| -20.8 \|  \| -179.6 \|  \| 4.7 \|  \| Yes \| \| *  3. \|  \| Asn29-Leu32 \|  \| NNGL \|  \| II \|  \| -68.7 \|  \| 123.5 \|  \| -68.7 \|  \| 99.7 \|  \| -30.1 \|  \| - \|  \| 5.4 \|  \| No \| \| *  4. \|  \| Phe46-Leu49 \|  \| FPIL \|  \| IV \|  \| -60.7 \|  \| -29.1 \|  \| -28.4 \|  \| -60.9 \|  \| -46.2 \|  \| -57.6 \|  \| 5.8 \|  \| No \| \| *  5. \|  \| Asp91-Lys94 \|  \| DATK \|  \| I \|  \| -55.1 \|  \| -33.2 \|  \| - \|  \| -65.9 \|  \| -31.4 \|  \| 50.5 \|  \| 6.0 \|  \| No \| \|  \| \| \| \| \| \| \| \| \| \| \| \| \| \| \| \| \| \| \| \| \| \| \| \| 6. \|  \| Thr138-Pro141 \|  \| TSDP \|  \| IV \|  \| -54.9 \|  \| -29.3 \|  \| -175.7 \|  \| -115.1 \|  \| 79.3 \|  \| -67.7 \|  \| 5.7 \|  \| Yes \| \| 7. \|  \| Asp140-Ile143 \|  \| DPLI \|  \| I \|  \| -62.2 \|  \| -21.8 \|  \| -21.3 \|  \| -83.4 \|  \| -18.7 \|  \| -70.3 \|  \| 5.4 \|  \| No \| \| 8. \|  \| Lys157-Leu160 \|  \| KEGL \|  \| II \|  \| -61.8 \|  \| 136.1 \|  \| -170.4 \|  \| 98.7 \|  \| -25.4 \|  \| - \|  \| 5.8 \|  \| No \| \| 9. \|  \| Asp172-Phe175 \|  \| DPVF \|  \| IV \|  \| -61.5 \|  \| 131.8 \|  \| -28.6 \|  \| 101.6 \|  \| -79.1 \|  \| 176.4 \|  \| 5.4 \|  \| Yes \| \|  \| \| \| \| \| \| \| \| \| \| \| \| \| \| \| \| \| \| \| \| \| \| \| |
|  |
|  |
| **h) Gamma turns** |
|  |
| \|  \| \| \| \| \| \| \| \| \| \| \| \| \| \| \| \| \| \| \| \| --- \| --- \| --- \| --- \| --- \| --- \| --- \| --- \| --- \| --- \| --- \| --- \| --- \| --- \| --- \| --- \| --- \| --- \| --- \| \|  \|  \|  \|  \|  \|  \|  \|  \| **Turn** \|  \| **Residue i+1** \| \| \| \| \|  \| **i to i+2** \|  \|  \| \|  \|  \|  \|  \|  \|  \|  \|  \|  \|  \|  \| \| \| \| \|  \|  \|  \|  \| \| **No.** \|  \| **Start** \|  \| **End** \|  \| **Sequence*** \|  \| **type** \|  \| **Phi** \|  \| **Psi** \|  \| **Chi1** \|  \| **CA-dist** \|  \|  \| \|  \| \| \| \| \| \| \| \| \| \| \| \| \| \| \| \| \| \| \| \| 1. \|  \| Gly13 \|  \| Phe15 \|  \| G P F \|  \| INVERSE \|  \| -76.1 \|  \| 77.2 \|  \| 33.2 \|  \| 5.9 \|  \|  \| \|  \| \| \| \| \| \| \| \| \| \| \| \| \| \| \| \| \| \| \| |

**Detailed secondary structure in ICP 84023 showing a) Secondary structure summary b) beta sheet c) beta hairpins, d) beta strands, e) helices, f) helix-helix interaction, g) beta turns, h) gamma turns**

**a) Secondary structure summary**

|  | | | | | | | | |
| --- | --- | --- | --- | --- | --- | --- | --- | --- |
| **Strand** |  | **Alpha helix** |  | **3-10 helix** |  | **Other** |  | **Total residues** |
|  | | | | | | | | |
| **4 (2.0%)** |  | **96 (46.8%)** |  | **12 (5.9%)** |  | **93 (45.4%)** |  | **205** |

**b) Beta sheet**

| \| **Sheet** \|  \| **View** \|  \| **No. of strands** \|  \| **Type** \|  \| **Barrel** \|  \| **Topology** \| \| --- \| --- \| --- \| --- \| --- \| --- \| --- \| --- \| --- \| --- \| --- \| \|  \| \| \| \| \| \| \| \| \| \| \| \| A \|  \|  \|  \| 2 \|  \| Antiparallel \|  \| No \|  \| 1 \| \|  \| \| \| \| \| \| \| \| \| \| \| |
| --- | --- | --- | --- | --- | --- | --- | --- | --- | --- | --- | --- | --- | --- | --- | --- | --- | --- | --- | --- | --- | --- | --- | --- | --- | --- | --- | --- | --- | --- | --- | --- | --- | --- | --- | --- | --- | --- | --- | --- | --- | --- | --- | --- | --- |
|  |
|  |
| **c) Beta hairpins** |
|  |
| \|  \| \| \| \| \| \| \| \| \| \| \| \| \| \| \| \| --- \| --- \| --- \| --- \| --- \| --- \| --- \| --- \| --- \| --- \| --- \| --- \| --- \| --- \| --- \| \|  \|  \| **Strand 1** \| \| \| \| \|  \| **Strand 2** \| \| \| \| \|  \| **Hairpin** \| \|  \|  \|  \| \| \| \| \|  \|  \| \| \| \| \|  \|  \| \| **No.** \|  \| **Start** \|  \| **End** \|  \| **Length** \|  \| **Start** \|  \| **End** \|  \| **Length** \|  \| **class** \| \|  \| \| \| \| \| \| \| \| \| \| \| \| \| \| \| \| 1. \|  \| Ala125 \|  \| Ala126 \|  \| 2 \|  \| Gly135 \|  \| Pro136 \|  \| 2 \|  \| 8:8 \| \|  \| \| \| \| \| \| \| \| \| \| \| \| \| \| \| |
| **d) Beta strand** |
|  |
|  |
| \| **No.** \|  \| **Start** \|  \| **End** \|  \| **Sheet** \|  \| **No. resid** \|  \| **Edge** \|  \| **Sequence** \| \| --- \| --- \| --- \| --- \| --- \| --- \| --- \| --- \| --- \| --- \| --- \| --- \| --- \| \|  \| \| \| \| \| \| \| \| \| \| \| \| \| \| 1. \|  \| Ala125 \|  \| Ala126 \|  \| A \|  \| 2 \|  \| Yes \|  \| AA \| \| 2. \|  \| Gly135 \|  \| Pro136 \|  \| A \|  \| 2 \|  \| Yes \|  \| GP \| \|  \| \| \| \| \| \| \| \| \| \| \| \| \| |
|  |
|  |
|  |
| **e) Helices** |
|  |
| \|  \| \| \| \| \| \| \| \| \| \| \| \| \| \| \| \| \| \| \| \| \| \| \| \| --- \| --- \| --- \| --- \| --- \| --- \| --- \| --- \| --- \| --- \| --- \| --- \| --- \| --- \| --- \| --- \| --- \| --- \| --- \| --- \| --- \| --- \| --- \| \| **No.** \|  \| **Start** \|  \| **End** \|  \| **Type** \|  \| **No. resid** \|  \| **Length** \|  \| **Unit rise** \|  \| **Residues per turn** \|  \| **Pitch** \|  \| **Deviation from ideal** \|  \| **Sequence** \|  \|  \| \|  \| \| \| \| \| \| \| \| \| \| \| \| \| \| \| \| \| \| \| \| \| \| \| \| *1. \|  \| Ala2 \|  \| Thr4 \|  \| G \|  \| 3 \|  \| - \|  \| - \|  \| - \|  \| - \|  \| - \|  \| AGT \|  \|  \| \| *2. \|  \| Thr17 \|  \| Lys19 \|  \| G \|  \| 3 \|  \| - \|  \| - \|  \| - \|  \| - \|  \| - \|  \| TIK \|  \|  \| \| *3. \|  \| Pro21 \|  \| Ala25 \|  \| H \|  \| 5 \|  \| 8.15 \|  \| 1.50 \|  \| 3.60 \|  \| 5.40 \|  \| 21.2 \|  \| PAELA \|  \|  \| \| *4. \|  \| Gly27 \|  \| Asn29 \|  \| G \|  \| 3 \|  \| - \|  \| - \|  \| - \|  \| - \|  \| - \|  \| GAN \|  \|  \| \|  \| \| \| \| \| \| \| \| \| \| \| \| \| \| \| \| \| \| \| \| \| \| \| \| 5. \|  \| Leu32 \|  \| Glu45 \|  \| H \|  \| 14 \|  \| 22.64 \|  \| 1.59 \|  \| 3.95 \|  \| 6.27 \|  \| 30.2 \|  \| LDIAVRLLEPIKAE \|  \|  \| \| 6. \|  \| Tyr51 \|  \| Ile65 \|  \| H \|  \| 15 \|  \| 22.60 \|  \| 1.47 \|  \| 3.66 \|  \| 5.37 \|  \| 10.5 \|  \| YAYFYQLAGVVAVEI \|  \|  \| \| 7. \|  \| Ser96 \|  \| Phe103 \|  \| H \|  \| 8 \|  \| 11.94 \|  \| 1.45 \|  \| 3.76 \|  \| 5.45 \|  \| 14.1 \|  \| SDHLRDVF \|  \|  \| \| 8. \|  \| Asp111 \|  \| Ser118 \|  \| H \|  \| 8 \|  \| 12.86 \|  \| 1.55 \|  \| 3.62 \|  \| 5.61 \|  \| 12.7 \|  \| DQDIVALS \|  \|  \| \|  \| \| \| \| \| \| \| \| \| \| \| \| \| \| \| \| \| \| \| \| \| \| \| \| 9. \|  \| Gly119 \|  \| Thr122 \|  \| G \|  \| 4 \|  \| 8.24 \|  \| 1.96 \|  \| 3.26 \|  \| 6.40 \|  \| 38.4 \|  \| GGHT \|  \|  \| \| 10. \|  \| Lys128 \|  \| Ser131 \|  \| H \|  \| 4 \|  \| 4.78 \|  \| 1.15 \|  \| 4.26 \|  \| 4.88 \|  \| 41.3 \|  \| KERS \|  \|  \| \| 11. \|  \| Ser147 \|  \| Ser154 \|  \| H \|  \| 8 \|  \| 12.75 \|  \| 1.51 \|  \| 3.80 \|  \| 5.73 \|  \| 17.3 \|  \| SHIKELLS \|  \|  \| \| 12. \|  \| Pro164 \|  \| Ser171 \|  \| H \|  \| 8 \|  \| 12.84 \|  \| 1.55 \|  \| 3.64 \|  \| 5.63 \|  \| 17.6 \|  \| PSDKALLS \|  \|  \| \|  \| \| \| \| \| \| \| \| \| \| \| \| \| \| \| \| \| \| \| \| \| \| \| \| 13. \|  \| Phe175 \|  \| Ala184 \|  \| H \|  \| 10 \|  \| 15.62 \|  \| 1.50 \|  \| 3.59 \|  \| 5.40 \|  \| 3.1 \|  \| FRPLVEKYAA \|  \|  \| \| 14. \|  \| Glu186 \|  \| Ser201 \|  \| H \|  \| 16 \|  \| 24.17 \|  \| 1.47 \|  \| 3.71 \|  \| 5.45 \|  \| 12.3 \|  \| EDAIFADYAVAHHKLS \|  \|  \| \|  \| \| \| \| \| \| \| \| \| \| \| \| \| \| \| \| \| \| \| \| \| \| \| |
|  |
| **f) Helix- helix interactions**   \| \|  \|  \|  \|  \| \|  \| **Helix** \| \|  \|  \|  \|  \|  \| **Interaction** \| \|  \| **No. interacting residues** \| \| \| \| \| \| --- \| --- \| --- \| --- \| --- \| --- \| --- \| --- \| --- \| --- \| --- \| --- \| --- \| --- \| --- \| --- \| --- \| --- \| --- \| --- \| --- \| \|  \|  \|  \|  \| \|  \|  \| \|  \|  \|  \|  \|  \|  \| \|  \|  \| \| \| \| \| \| **No.** \|  \|  \| **Helices** \| \|  \| **types** \| \|  \| **Distance (Å)** \|  \| **Angle (°)** \|  \| **type** \| \|  \| **Total** \|  \| **Helix 1** \|  \| **Helix 2** \| \| 1. \|  \|  \| 3 \| 5 \|  \| H \| H \|  \| 9.7 \|  \| 153.1 \|  \| c \| n \|  \| 3 \|  \| 1 \|  \| 3 \| \| 2. \|  \|  \| 5 \| 6 \|  \| H \| H \|  \| 11.0 \|  \| 110.4 \|  \| I \| I \|  \| 10 \|  \| 5 \|  \| 5 \| \| 3. \|  \|  \| 5 \| 10 \|  \| H \| H \|  \| 9.9 \|  \| -96.7 \|  \| N \| I \|  \| 2 \|  \| 2 \|  \| 1 \| \| 4. \|  \|  \| 6 \| 14 \|  \| H \| H \|  \| 10.8 \|  \| 171.1 \|  \| N \| C \|  \| 8 \|  \| 4 \|  \| 4 \| \| 5. \|  \|  \| 7 \| 8 \|  \| H \| H \|  \| 10.0 \|  \| 107.3 \|  \| I \| I \|  \| 5 \|  \| 3 \|  \| 3 \| \| 6. \|  \|  \| 7 \| 12 \|  \| H \| H \|  \| 7.9 \|  \| -91.7 \|  \| N \| I \|  \| 5 \|  \| 2 \|  \| 4 \| \| 7. \|  \|  \| 7 \| 13 \|  \| H \| H \|  \| 12.3 \|  \| 84.5 \|  \| N \| N \|  \| 3 \|  \| 3 \|  \| 1 \| \| 8. \|  \|  \| 7 \| 14 \|  \| H \| H \|  \| 13.8 \|  \| 50.4 \|  \| C \| C \|  \| 1 \|  \| 1 \|  \| 1 \| \| 9. \|  \|  \| 8 \| 9 \|  \| H \| G \|  \| 0.2 \|  \| 9.5 \|  \| C \| I \|  \| 10 \|  \| 4 \|  \| 4 \| \| 10. \|  \|  \| 8 \| 11 \|  \| H \| H \|  \| 8.3 \|  \| 92.5 \|  \| c \| n \|  \| 2 \|  \| 2 \|  \| 2 \| \| 11. \|  \|  \| 8 \| 12 \|  \| H \| H \|  \| 11.3 \|  \| -140.1 \|  \| I \| I \|  \| 4 \|  \| 3 \|  \| 2 \| \| 12. \|  \|  \| 8 \| 13 \|  \| H \| H \|  \| 9.8 \|  \| -48.4 \|  \| I \| I \|  \| 12 \|  \| 5 \|  \| 5 \| \| 13. \|  \|  \| 8 \| 14 \|  \| H \| H \|  \| 8.3 \|  \| -102.0 \|  \| I \| I \|  \| 12 \|  \| 4 \|  \| 8 \| \| 14. \|  \|  \| 9 \| 11 \|  \| G \| H \|  \| 6.9 \|  \| 97.4 \|  \| I \| N \|  \| 6 \|  \| 3 \|  \| 3 \| \| 15. \|  \|  \| 9 \| 12 \|  \| G \| H \|  \| 11.2 \|  \| -132.6 \|  \| N \| I \|  \| 2 \|  \| 1 \|  \| 2 \| \| 16. \|  \|  \| 9 \| 14 \|  \| G \| H \|  \| 9.5 \|  \| -107.5 \|  \| N \| I \|  \| 3 \|  \| 2 \|  \| 2 \| \| 17. \|  \|  \| 11 \| 12 \|  \| H \| H \|  \| 10.5 \|  \| -82.4 \|  \| I \| I \|  \| 7 \|  \| 3 \|  \| 4 \| \| 18. \|  \|  \| 11 \| 13 \|  \| H \| H \|  \| 7.6 \|  \| -120.9 \|  \| I \| I \|  \| 10 \|  \| 4 \|  \| 5 \| \| 19. \|  \|  \| 11 \| 14 \|  \| H \| H \|  \| 9.6 \|  \| 110.9 \|  \| N \| I \|  \| 1 \|  \| 1 \|  \| 1 \| \| 20. \|  \|  \| 12 \| 13 \|  \| H \| H \|  \| 4.4 \|  \| -101.4 \|  \| c \| n \|  \| 7 \|  \| 4 \|  \| 3 \| \| 21. \|  \|  \| 13 \| 14 \|  \| H \| H \|  \| 4.9 \|  \| -118.5 \|  \| c \| n \|  \| 8 \|  \| 4 \|  \| 4 \| \| \| --- \| --- \| --- \| --- \| --- \| --- \| --- \| --- \| --- \| --- \| --- \| --- \| --- \| --- \| --- \| --- \| --- \| --- \| --- \| --- \| --- \| --- \| --- \| --- \| --- \| --- \| --- \| --- \| --- \| --- \| --- \| --- \| --- \| --- \| --- \| --- \| --- \| --- \| --- \| --- \| --- \| --- \| --- \| --- \| --- \| --- \| --- \| --- \| --- \| --- \| --- \| --- \| --- \| --- \| --- \| --- \| --- \| --- \| --- \| --- \| --- \| --- \| --- \| --- \| --- \| --- \| --- \| --- \| --- \| --- \| --- \| --- \| --- \| --- \| --- \| --- \| --- \| --- \| --- \| --- \| --- \| --- \| --- \| --- \| --- \| --- \| --- \| --- \| --- \| --- \| --- \| --- \| --- \| --- \| --- \| --- \| --- \| --- \| --- \| --- \| --- \| --- \| --- \| --- \| --- \| --- \| --- \| --- \| --- \| --- \| --- \| --- \| --- \| --- \| --- \| --- \| --- \| --- \| --- \| --- \| --- \| --- \| --- \| --- \| --- \| --- \| --- \| --- \| --- \| --- \| --- \| --- \| --- \| --- \| --- \| --- \| --- \| --- \| --- \| --- \| --- \| --- \| --- \| --- \| --- \| --- \| --- \| --- \| --- \| --- \| --- \| --- \| --- \| --- \| --- \| --- \| --- \| --- \| --- \| --- \| --- \| --- \| --- \| --- \| --- \| --- \| --- \| --- \| --- \| --- \| --- \| --- \| --- \| --- \| --- \| --- \| --- \| --- \| --- \| --- \| --- \| --- \| --- \| --- \| --- \| --- \| --- \| --- \| --- \| --- \| --- \| --- \| --- \| --- \| --- \| --- \| --- \| --- \| --- \| --- \| --- \| --- \| --- \| --- \| --- \| --- \| --- \| --- \| --- \| --- \| --- \| --- \| --- \| --- \| --- \| --- \| --- \| --- \| --- \| --- \| --- \| --- \| --- \| --- \| --- \| --- \| --- \| --- \| --- \| --- \| --- \| --- \| --- \| --- \| --- \| --- \| --- \| --- \| --- \| --- \| --- \| --- \| --- \| --- \| --- \| --- \| --- \| --- \| --- \| --- \| --- \| --- \| --- \| --- \| --- \| --- \| --- \| --- \| --- \| --- \| --- \| --- \| --- \| --- \| --- \| --- \| --- \| --- \| --- \| --- \| --- \| --- \| --- \| --- \| --- \| --- \| --- \| --- \| --- \| --- \| --- \| --- \| --- \| --- \| --- \| --- \| --- \| --- \| --- \| --- \| --- \| --- \| --- \| --- \| --- \| --- \| --- \| --- \| --- \| --- \| --- \| --- \| --- \| --- \| --- \| --- \| --- \| --- \| --- \| --- \| --- \| --- \| --- \| --- \| --- \| --- \| --- \| --- \| --- \| --- \| --- \| --- \| --- \| --- \| --- \| --- \| --- \| --- \| --- \| --- \| --- \| --- \| --- \| --- \| --- \| --- \| --- \| --- \| --- \| --- \| --- \| --- \| --- \| --- \| --- \| --- \| --- \| --- \| --- \| --- \| --- \| --- \| --- \| --- \| --- \| --- \| --- \| --- \| --- \| --- \| --- \| --- \| --- \| --- \| --- \| --- \| --- \| --- \| --- \| --- \| --- \| --- \| --- \| --- \| --- \| --- \| --- \| --- \| --- \| --- \| --- \| --- \| --- \| --- \| --- \| --- \| --- \| --- \| --- \| --- \| --- \| --- \| --- \| --- \| --- \| --- \| --- \| --- \| --- \| --- \| --- \| --- \| --- \| --- \| --- \| --- \| --- \| --- \| --- \| --- \| --- \| --- \| --- \| --- \| --- \| --- \| --- \| --- \| --- \| --- \| --- \| --- \| --- \| --- \| --- \| --- \| --- \| --- \| --- \| --- \| --- \| --- \| --- \| --- \| --- \| --- \| --- \| --- \| --- \| --- \| --- \| --- \| --- \| --- \| --- \| --- \| --- \| --- \| --- \| --- \| --- \| --- \| --- \| --- \| --- \| --- \| --- \| --- \| --- \| --- \| --- \| --- \| --- \| --- \| --- \| --- \| --- \| --- \| --- \| --- \| --- \| --- \| --- \| --- \| --- \| --- \| --- \| --- \| --- \| --- \| --- \| --- \| --- \| --- \| --- \| --- \| --- \| --- \| --- \| --- \| --- \| --- \| --- \| --- \| --- \| --- \| --- \| --- \| --- \| --- \| --- \| --- \| --- \| --- \| --- \| \|  \| \| \| **g) Beta turns** \| \| --- \| \|  \| \| \|  \| \| \| \| \| \| \| \| \| \| \| \| \| \| \| \| \| \| \| \| \| \| \| \| --- \| --- \| --- \| --- \| --- \| --- \| --- \| --- \| --- \| --- \| --- \| --- \| --- \| --- \| --- \| --- \| --- \| --- \| --- \| --- \| --- \| --- \| --- \| \|  \|  \|  \|  \|  \|  \| **Turn** \|  \| **Residue i+1** \| \| \| \| \|  \| **Residue i+2** \| \| \| \| \|  \| **i to i+3** \|  \|  \| \|  \|  \|  \|  \|  \|  \|  \|  \|  \| \| \| \| \|  \|  \| \| \| \| \|  \|  \|  \|  \| \| **No.** \|  \| **Turn** \|  \| **Sequence*** \|  \| **type** \|  \| **Phi** \|  \| **Psi** \|  \| **Chi1** \|  \| **Phi** \|  \| **Psi** \|  \| **Chi1** \|  \| **CA-dist** \|  \| **H-bond** \| \|  \| \| \| \| \| \| \| \| \| \| \| \| \| \| \| \| \| \| \| \| \| \| \| \| *  1. \|  \| Asp6-Thr9 \|  \| DVST \|  \| IV \|  \| -58.6 \|  \| -40.0 \|  \| -176.9 \|  \| -42.4 \|  \| -69.8 \|  \| 178.6 \|  \| 6.4 \|  \| Yes \| \| *  2. \|  \| Val7-Lys10 \|  \| VSTK \|  \| I \|  \| -42.4 \|  \| -69.8 \|  \| 178.6 \|  \| -93.7 \|  \| 2.7 \|  \| -175.5 \|  \| 4.7 \|  \| Yes \| \| *  3. \|  \| Asn29-Leu32 \|  \| NNGL \|  \| II \|  \| -67.0 \|  \| 127.2 \|  \| -72.2 \|  \| 94.5 \|  \| -26.1 \|  \| - \|  \| 5.4 \|  \| No \| \| *  4. \|  \| Phe46-Leu49 \|  \| FPIL \|  \| IV \|  \| -60.9 \|  \| -28.1 \|  \| -26.1 \|  \| -58.2 \|  \| -39.4 \|  \| -55.0 \|  \| 5.9 \|  \| No \| \| *  5. \|  \| Asp91-Lys94 \|  \| DATK \|  \| I \|  \| -54.4 \|  \| -35.5 \|  \| - \|  \| -63.6 \|  \| -34.3 \|  \| -43.1 \|  \| 5.6 \|  \| No \| \|  \| \| \| \| \| \| \| \| \| \| \| \| \| \| \| \| \| \| \| \| \| \| \| \| 6. \|  \| Thr138-Pro141 \|  \| TSDP \|  \| VIII \|  \| -56.2 \|  \| -31.4 \|  \| -51.0 \|  \| -119.8 \|  \| 85.6 \|  \| -81.8 \|  \| 5.7 \|  \| Yes \| \| 7. \|  \| Asp140-Ile143 \|  \| DPLI \|  \| I \|  \| -64.8 \|  \| -7.6 \|  \| 31.2 \|  \| -117.6 \|  \| 21.9 \|  \| -61.4 \|  \| 5.7 \|  \| No \| \| 8. \|  \| Lys157-Leu160 \|  \| KEGL \|  \| II \|  \| -61.0 \|  \| 135.2 \|  \| 70.7 \|  \| 97.9 \|  \| -29.4 \|  \| - \|  \| 5.8 \|  \| No \| \| 9. \|  \| Asp172-Phe175 \|  \| DTVF \|  \| IV \|  \| -50.6 \|  \| -44.0 \|  \| -38.5 \|  \| -82.5 \|  \| -40.0 \|  \| -56.8 \|  \| 5.3 \|  \| Yes \| \|  \| \| \| \| \| \| \| \| \| \| \| \| \| \| \| \| \| \| \| \| \| \| \| \| \| \|  \| |
| \| **h) gamma turns** \| \| --- \| \|  \| \| \|  \| \| \| \| \| \| \| \| \| \| \| \| \| \| \| \| \| \| \| \| --- \| --- \| --- \| --- \| --- \| --- \| --- \| --- \| --- \| --- \| --- \| --- \| --- \| --- \| --- \| --- \| --- \| --- \| --- \| \|  \|  \|  \|  \|  \|  \|  \|  \| **Turn** \|  \| **Residue i+1** \| \| \| \| \|  \| **i to i+2** \|  \|  \| \|  \|  \|  \|  \|  \|  \|  \|  \|  \|  \|  \| \| \| \| \|  \|  \|  \|  \| \| **No.** \|  \| **Start** \|  \| **End** \|  \| **Sequence*** \|  \| **type** \|  \| **Phi** \|  \| **Psi** \|  \| **Chi1** \|  \| **CA-dist** \|  \|  \| \|  \| \| \| \| \| \| \| \| \| \| \| \| \| \| \| \| \| \| \| \| 1. \|  \| Gly13 \|  \| Phe15 \|  \| G P F \|  \| INVERSE \|  \| -75.2 \|  \| 72.0 \|  \| 33.0 \|  \| 6.0 \|  \|  \| \|  \| \| \| \| \| \| \| \| \| \| \| \| \| \| \| \| \| \| \| \| |
